# Abstracts of scientific papers and sessions, CPA Congress 2010

**DOI:** 10.3138/physio.62.supp

**Published:** 2010-07

**Keywords:** Physio10, Canadian Physiotherapy Association Congress 2010, St. John's, Newfoundland, Canada, Abstracts, Scientific sessions, Health Promotion, public and patient education, injury prevention, determinants of health, role of physiotherapists in primary health care, mobility, population health services, exercise prescription, chronic disease prevention, cardio-respiratory, neurological, musculoskeletal, Leadership in Action, physiotherapy leadership development, innovative models of practice, public, private and independent practice management of physiotherapy services, evolving physiotherapist roles, health human resources management strategies, inter-professional collaboration and education strategies, innovative service delivery models, health system reform, Neuromusculoskeletal Practice, promotion of research, practice and patient/client care in the neuromusculoskeletal area, evidence based practice model, clinical experience, patient and client preference, clinical decision making, assessment and treatment of disability that arises from both spinal and peripheral pathologies, clinical skills and clinical reasoning modules in orthopaedic practice, advances in research related to neuromusculoskeletal practice, Merging Research and Practice, fundamental scientific research, clinical research and the reciprocal transfer of knowledge between clinical practice and research, active transfer strategies for informing clinical practice through information technologies, informing the development of clinical research questions, basic science or clinically focused research on targeted physiotherapy interventions, implementation and management strategies, benchmarking areas of practice, post-operative management of acute care patients

**Purpose/Objectives and Rationale:** Adults over the age of 65 are at risk of falls, with risk progressing with age. The presence of additional chronic conditions such as arthritis can further amplify this risk. The objective of this study was to evaluate the effect of aquatic exercise, a common exercise medium used for adults with arthritis, and education on fall risk factors in older adults with hip osteoarthritis (OA). **Relevance to the Physiotherapy Profession:** Determining the best type and delivery of exercise and education for higher fall risk populations assists clinicians in developing effective prevention programs. **Materials and Methods:** Seventy-nine adults, 65 years of age or older with hip OA and at least 1 fall risk factor, were randomly assigned to one of three groups: Aquatic-Education (AE, aquatic exercise 2/week with 1/week group education), Aquatic (A, 2/week aquatic exercise) and Control (C, usual activity). Balance (Berg Balance Scale), falls-efficacy (Activities Balance Confidence Scale), dual task function (Timed Up and Go cognitive), functional performance (chair stands), and walking performance (6 minute walk) were measured pre and post intervention or control period (11 weeks). **Analysis:** Between group differences in fall risk variables was examined using a general linear multivariate analysis (MANCOVA) using baseline values as co-variates. **Results:** There was a significant improvement in fall risk factors (*p *= 0.038) where AE improved in falls-efficacy compared to C and in functional performance compared to A and C. **Conclusions:** The combination of aquatic exercise and education was effective in improving fall risk factors in older adults with lower extremity arthritis.

