# Supplementary material for: Abstracts of scientific papers and sessions, CPA Congress 2010
Source: Physiother Can. 2010 Jul;62(Suppl 1):1–54. doi: 10.3138/physio.62.supp (PMC2890191; doi:10.3138/physio.62.supp)
Supplement: Related texts

# Abstract: A032 – Health, lifestyle and aging with Multiple Sclerosis: A qualitative study

**Published:** 2010-07

**Authors:** Michelle Ploughman, Mark Austin, Anne Kearney, Michelle Murdoch, Mark Stefanelli, Marshall Godwin

**Affiliations:** Post-doctoral Fellow Primary Healthcare Research Unit, Memorial University; Eastern Health; Community Health and Humanities, Faculty of Medicine, Memorial University; Independent Living Resource Centre, St. John's NL; Neurology, Faculty of Medicine, Memorial University; Primary Healthcare Research Unit, Faculty of Medicine, Memorial University

**Purpose/Objectives and Rationale:** MS symptoms and progression are highly variable. One of the most important questions MS patients ask is, ‘ What should I expect in the future and how will the disease progress?’.The aim of this study was to explore health and lifestyle factors that may influence aging well with MS from the patient's point of view. **Relevance to the Physiotherapy Profession:** Findings from this study will help physiotherapists and other health providers provide evidence-informed advice to people aging with MS. **Materials and Methods:** We recruited individuals over the age of 60 with MS for more than 20 years from an outpatient MS clinic. Health aging and lifestyle were explored using in-depth semi-structured interviews and data saturation was reached at the eighteenth participant. **Analysis:** Interview audiotapes were transcribed verbatim and themes derived using NVIVO8. Three investigators developed initial coding and codes were further validated and adapted in an iterative manner. Thematic analysis described themes and relationships among codes. **Results:** Participants' (14 F/4 M) mean age was 66.5 ± 6.7 yrs and time from first symptoms was 33.5 ± 8.22 yrs. The typical participant was female, married, living at home with help for personal care and using a wheelchair for mobility. Thematic analysis suggested that a positive outlook, personal resilience, support of the spouse, financial flexibility and the absence of cognitive deficits as fundamental to healthy aging. **Conclusions:** Our participants identified factors, outside of MS-related symptoms, that were important to maintaining quality of life as they aged. These findings suggest that MS health care teams must address the full spectrum of health when suggesting strategies to promote healthy aging.

# Abstract: A033 – The complexity of dual-tasking affects gait variability in people with mild cognitive impairment

**Published:** 2010-07

**Authors:** Susan W Muir, Mark Speechley, Michael Borrie, Manuel Montero-Odasso

**Affiliations:** 1 Department of Geriatric Medicine, Parkwood Hospital and Division of Geriatric Medicine, University of Western Ontario, London, ON, Canada; 2 Department of Epidemiology & Biostatistics, University of Western Ontario, London, ON, Canada

**Purpose/Objectives & Rationale:** Gait variability (G-var), stride time variation, depends on brain cortical control and is a marker for adverse events in older adults. The effect of different dual-task challenges on gait in older adults with Mild Cognitive Impairment (MCI) has not been evaluated. The objective was to assess if dual-task challenges with increasing complexity affect G-var more in people with MCI than normal controls. **Relevance to the Physiotherapy Profession:** People with MCI have an increased fall risk. Dual-task challenges may be a sensitive way to expose gait impairment facilitating early rehabilitation intervention. **Materials and Methods:** Cross-sectional study of 43 older adults with newly diagnosed MCI and 25 age-matched normal controls. Velocity and stride time variation were assessed under single (usual walking) and dual-tasks (naming animals and subtracting serials sevens), using the GAITRite® System. **Analysis:** Two-way repeated measures ANOVA. **Results:** A statistically significant difference was found between and within groups (*p *= 0.0165) of increasing G-var, consistent with a dose response relationship, and decreased gait velocity within groups with increasing dual-task complexity. The magnitude of increase on G-var across tasks was greater for the MCI group (2.2% to 10.1%) than the control group (1.9% to 3.7%). **Conclusions:** Dual-task load significantly increased stride time variation in the MCI group compared with controls. This effect was larger than changes seen on gait velocity. These findings underscore the importance of the cognitive component of gait in people with MCI and the effect of different dual-tasks. Complex dual-tasks may provide a clinical methodology to detect people at higher fall risk.

# Abstract: A052 – Nintendo Wii™ as a cardiovascular training mode for people post-stroke

**Published:** 2010-07

**Authors:** M MacKay-Lyons, G Creaser, A Macdonald, J McKenna, S Murphy

**Affiliations:** School of Physiotherapy, Dalhousie University, Halifax, NS, B3H 3J5, Canada

**Purpose/Objectives and Rationale:** People post-stroke are often extremely deconditioned despite evidence that aerobic training is safe and effective for this population. The purpose was to determine if a low-cost virtual reality system could elicit physiological responses adequate for cardiovascular training post-stroke. **Relevance to the Physiotherapy Profession:** Innovative, inexpensive strategies are needed for people post-stroke to engage in habitual exercise as part of a healthy lifestyle. **Materials and Methods:** Energy expenditure was measured in 25 people post-stroke (61.3+14.2 years; 21 males) while they played Wii Fit™ Jogging for 10–15 minutes, wearing a harness to prevent falls. Oxygen consumption (VO2) and heart rate (HR) were recorded continuously using portable metabolic monitoring system, and rating of perceived exertion (RPE0–10) was assessed at 2-minute intervals. **Analysis:** To determine the percentage of participants who exceeded the threshold for training, mean values during steady-state jogging were calculated in reference to the following training targets: (i) percent predicted maximal HR (%HRmaxpred) > 64%, (ii) percent predicted HR reserve (%HRRpred) > 40%, (iii) metabolic equivalents (METs) > 3, and (iv) RPE0–10 > 4. **Results:** Across participants, steady-state means for %HRmaxpred, %HRRpred, METs, and RPE0–10 were 43.6+19.7, 70.0+12.0, 3.5+0.9, and 4.8+1.7, respectively. 71% of participants exceeded targets for %HRmaxpred and METs, 67% for RPE0–10, and 52% for %HRRpred. 11 participants met all 4 targets and 3 met none. **Conclusions:** Nintendo Wii™ elicited physiological responses adequate for aerobic training, thus supporting the use of Nintendo Wii™ as a cardiovascular training mode for people post-stroke. This intervention has clinical appeal because it is an inexpensive and enjoyable alternative to more traditional training strategies.

# Abstract: A059 – More exercise—A health quandary for seniors

**Published:** 2010-07

**Authors:** VR O'Neill, T Nilsson, L Begley

**Affiliations:** Centre for Health and Aging, University of Prince Edward Island

**Purpose/Objectives and Rationale:** With growing evidence for the efficacy of moderate physical activity, the PEI Centre for Health and Ageing sought to gather information on exercise behaviour among seniors. **Relevance to the Physiotherapy Profession:** Physiotherapists have the practical knowledge and skills to provide encouragement, monitoring and reassurance to their seniors clients, attempting to increase activity levels. **Materials and Methods:** A telephone survey was conducted using a random telephone number selection system to obtain a representative sample. A pilot-tested, consultatively developed questionnaire was administered to 180 seniors: (males = 49, female = 131; urba*n *= 109, rural = 71), in the summer of 2000. **Analysis:** 74% of Island seniors surveyed reported some form of exercise, 40% exercised daily, and 36% did not exercise. Exercise levels were higher among men and among those who exercised by themselves. Those who exercised in groups tended to exercise a few times a week, while those who exercised by themselves, exercised daily. Rural seniors exercised less than urban seniors. **Results:** The perceived Chief Benefit of Exercise was ‘improved health’ (X2 = 44.65, df8,*n *= 262, required:26.12, *p *= 0.001). Yet the perceived Chief Barrier to MORE exercise was the fear of exercise-induced health problems (X2 = 5.39, df1, *n *= 141, required:5.41, *p *= 0.02). **Conclusions:** 58% of those surveyed wished to increase their physical activity levels. Although seniors who exercised reported ‘better health’ as their primary motivator for exercise, ‘health-related concerns’ emerged as the main barrier cited to increasing exercise levels. Physiotherapists have the ideal set of knowledge and skills to assist seniors, as they face this potentially perplexing health quandary. Although this data could benefit from updating, the results may be of interest to physiotherapists.

# Abstract: A062 – A systematic review of preoperative rehabilitation for patients undergoing total hip or knee arthroplasty

**Published:** 2010-07

**Authors:** Kathy M. Ikert, Kristin M. Long, Darren R. Bittner, Trevor B. Birmingham, Dianne M. Bryant

**Affiliations:** Strathroy Middlesex General Hospital, 395 Carrie Street, Strathroy, Ontario, Canada, N7G 3C9, Phone: 519–847–5295 (5543), Fax: 519–246–5928, Email: kathy.ikert@mha.tvh.ca; Eramosa Physiotherapy Associates, 380 Eramosa Rd. #28, Guelph, Ontario, Canada, N1E 6R2; Glenn W. Wark, 115 Bell Farm Rd. Unit 102, Barrie, Ontario, Canada, L4M 5G1; Box 41, Group 9, RR#2, Dugald, Manitoba, Canada, R0E 0K0; Elborn College, University of Western Ontario, London, Ontario, Canada, N6G 1H1; Elborn College, University of Western Ontario, London, Ontario, Canada, N6G 1H1

**Purpose/Objectives and Rationale:** To test the hypothesis that preoperative rehabilitation results in better postoperative outcomes for those undergoing total hip or knee arthroplasty (THA/TKA). **Relevance to the Physiotherapy Profession:** With an aging Canadian population, the incidence of osteoarthritis and resultant joint replacement is expected to increase. Preoperative rehabilitation is proposed to improve outcomes following THA/TKA, however, its effectiveness remains unclear. **Materials and Methods:** Following a literature search, two sets of reviewers collected articles meeting the following criteria: written in English, an RCT or systematic review, preoperative exercise and/or education intervention was implemented before undergoing THA and/or TKA and a postoperative outcome was measured. **Analysis:** Two teams of reviewers independently determined eligibility, extracted data and appraised study quality. We used a random effects model to pool data and our a priori hypothesis to address heterogeneity. **Results:** Twenty-four studies were included, eleven similar enough to combine in a meta-analysis. Three studies demonstrated that preoperative education was effective in reducing post-operative anxiety (SMD [standardized mean difference] = -0.33(95% CI [confidence interval]), -0.57 to -0.08) (*p *= 0.008). Exercise interventions for THA subjects resulted in significant improvements in Harris Hip Scores at 3 months (WMD [weighted mean difference] = 6.05, (95%CI), 1.29 to 10.81) (*p *= 0.01). There were no significant differences for the SF-36 (*p *= 0.15) or WOMAC (*p *= 0.14) when exercise was the intervention for TKA. **Conclusions:** Our results indicate that education can reduce postoperative anxiety and that exercise can improve outcomes in THA subjects. The studies regarding TKA subjects were limited in number and have considerable variability in interventions provided, limiting our ability to make any definitive conclusions in this group.

# Abstract: A063 – A Population Base approach to monitoring the physical function of persons with chronic disease in primary care

**Published:** 2010-07

**Authors:** Julie Richardson, Lori Letts, David Chan, Paul Stratford, Carri Hand, David Price, Linda Hilts, Liliana Coman, Mary Edwards, Sue Baptiste, Mary Law

**Affiliations:** 1 School of Rehabilitation, McMaster University, Hamilton, Ontario, Canada; 2 Department of Family Medicine, McMaster University, Hamilton, Ontario, Canada

**Purpose/Objectives and Rationale:** The purpose of this study was to determine whether: (1) Patients who receive the multi-component intervention show less functional decline than case matched controls, and (2) A multi-component intervention increases patient self-management and capacity building within a Family Health Team and increases the collaborative approach to Chronic Disease Management by the patient care provider. **Relevance to the Physiotherapy Profession:** It has been suggested that within primary care settings such as Family Health Teams population based physiotherapy services rather than a clinic based approach are more likely to be funded. **Materials and Methods:** A before-after design with case matched controls (*n *= 119) was used by physiotherapists and occupational therapists to deliver this intervention to patients: ≥44 years with at least one chronic disease and had at least 3 visits to the physician in the previous year and had some difficulty with physical functioning. The intervention included functional assessments, self management planning, a rehabilitation self management workshop and on-line monitoring of function. The intervention also included capacity building activities within the team: workshops, case reviews, small group problem based tutorials and the development of a flowsheet within the electronic medical record for patient care providers to monitor physical functioning. **Analysis:** One-way analysis of variance to test for between group differences. **Results:** There was a significant difference between initial and follow-up scores in levels of physical activity *p *= 0.0004, strength mesurements *p *= 0.0008 and in self-efficacy *p *= 0.02 in favour of the intervention group. **Conclusions:** This approach has demonstrated feasibility to managing physical functioning within chronic disease as a method of delivering rehabilitation services within primary care.

# Abstract: A067 – Reaching consensus on the feasibility and importance of recommendations for the physiotherapy management of obesity

**Published:** 2010-07

**Authors:** Elliot Alexander, Stephanie Rosenthal, Sara Guilcher, Cathy Evans

**Affiliations:** University of Toronto, ON; Dept. of Physical Therapy, University of Toronto, Toronto ON; Dept. of Physical Therapy, University of Toronto, Toronto ON; University of Toronto, Dept. of Physical Therapy, Toronto, ON

**Purpose/Objectives and Rationale:** To identify existing clinical practice guidelines for the management of obesity and reach consensus on feasibility and importance of individual recommendations for Canadian physiotherapy practice. **Relevance to the Physiotherapy Profession:** Nearly 60% of Canadians are overweight or obese. Physiotherapists treat patients with associated conditions but there is no clear understanding about how they should contribute to the management of obesity **Materials and Methods:** We identified recommendations for interventions from guidelines such as the “2006 Canadian clinical practice guidelines on the management and prevention of obesity in adults and children”. We used a modified Delphi approach to reach consensus on interventions for physiotherapy from clinical practice guidelines. Participants rated the importance and feasibility on a 9 point scale during two rounds of an e-survey and a conference call. **Analysis:** The mean and proportion of agreement were analyzed using descriptive statistics to determine inclusion in the recommendations. **Results:** Twenty-one physiotherapists were recruited using a snowball approach from diverse areas of practice. Seventeen (81.0 %) completed Survey 1. Ten (47.6%) were available for the conference call and completed Survey 2. Eight of 34 interventions received “High” mean ratings (7.00–9.00) in both importance and feasibility by at least 66.7% of participants. These interventions were primarily related to physical activity prescription. Lack of consensus was associated with differences in area of practice, available resources and clinical settings. **Conclusions:** There was consensus that physiotherapists should address physical activity with their adult clients who are overweight or obese. Other interventions were considered important but physiotherapists required further education and more time to make these practices feasible.

# Abstract: A072 – Functional electrical stimulation assisted cycling: Experiences of individuals with Spinal Cord Injury

**Published:** 2010-07

**Authors:** M Gattey, R Hydomako, B McLean, J O'Leary, K Shkopich, L Klassen

**Affiliations:** School of Physical Therapy, University of Saskatchewan, Saskatoon, SK, S7N 0W3, Canada

**Purpose/Objectives and Rationale:** To describe the experiences of individuals with spinal cord injuries who were prescribed functional electrical stimulation bicycles and to explore factors contributing to continued or discontinued bicycle use. **Relevance to the Physiotherapy Profession:** Individuals' experiences can inform the development of guidelines for bicycle prescription and optimization of bicycle use. **Materials and Methods:** Three individuals with paraplegia and two with quadriplegia participated in this mixed-method exploratory study. Participants each completed an equipment satisfaction questionnaire and a semi-structured interview. Interviews were recorded and transcribed verbatim. **Analysis:** Interview responses were coded, and then organized into themes using triangulation and member checking methods. Questionnaires were analyzed using descriptive statistical methods. Overlapping interview and questionnaire responses were compared for consistency. **Results:** All participants enjoyed the opportunity to exercise paralyzed extremities and to experience a ‘good physical workout’. All hoped the bike would restore pre-injury levels of physical activity and prevent secondary impairments and complications. Two participants specifically expressed the desire to maintain lower extremity muscle mass and bone density in the hopes that spinal cord research might make future ambulation possible. Two participants had discontinued bike use at the time of the study. The main barrier to continued use was dependence upon others for bike set up. All participants considered a strong support system important for facilitating ongoing bike use. **Conclusions:** Both those participants who had maintained bike use, and those who had not, experienced benefits and a sense of ‘normal’ physical activity from periods of use. Barriers and facilitators should be carefully weighed when considering prescription of functional electrical stimulation bicycles for home use.

# Abstract: A076 – The development of bone fit—A training program for health professionals on exercise and Osteoporosis

**Published:** 2010-07

**Authors:** Judi Laprade, Cathy Evans, Marla Roy

**Affiliations:** University of Toronto, Dept. of Anatomy; University of Toronto, Dept. of Physical Therapy; UHN, Toronto

**Purpose/Objectives and Rationale:** To identify patient and clinician information needs about osteoporosis, osteopenia and related exercise to inform the development of the Bone Fit certification program. **Relevance to the Physiotherapy Profession:** Osteoporosis affects one in four women and one in eight men over the age of 50. The cost of treating osteoporosis and resulting fractures is about $1.3 billion each year in Canada. Physiotherapists see many patients with fracture, deformity and pain secondary to osteoporosis. In 2005, The Ministry of Health and Long-Term Care announced Ontario's first Osteoporosis Strategy to reduce fractures, morbidity, mortality and costs through an integrated and comprehensive approach aimed at health promotion. One of the five focus areas is professional education. Osteoporosis Canada plans to implement a certification program for physiotherapists and exercise professionals called Bone Fit. **Materials and Methods:** We administered two surveys to inform the development of the program. The first elicited input from patients via the Osteoporosis Canada network about current needs for guidance in exercise and reasons for consulting professionals. The second was sent to physiotherapists, kinesiologists and certified exercise physiologists to gain perspective on their learning needs related to osteoporosis. **Analysis:** Descriptive statistics including mean and frequency distributions were calculated to determine items of importance to respondents. **Results:** 175 patients and 100 clinicians responded to the surveys. Areas important to patients and clinicians included: fracture risk assessment, exercise prescription, safe adaptations and alternative exercises, and comprehensive knowledge of the progression of osteoporosis. **Conclusions:** Information provided by patients and clinicians was used to customize the instructional content of the Bone Fit assessment and exercise prescription workshop.

# Abstract: A084 – The presence of upper extremity musculoskeletal impairments in patients with Hand-Arm Vibration Syndrome (HAVS)

**Published:** 2010-07

**Authors:** M Laing, L Di Nicolantonio, K Goncharova, M Pui, H Vas, R House, S Switzer-McIntyre

**Affiliations:** University of Toronto, Department of Physical Therapy

**Purpose/Objectives and Rationale:** To determine the presence of upper extremity musculoskeletal impairments in patients with Hand-Arm Vibration Syndrome and to describe the demographics of this population so as to enhance the assessment and improve patient care. **Relevance to the Physiotherapy Profession:** Recent research shows that these patients experience lower quality of life and have increased difficulty with activities of daily living. Determinng that these impairments are due to known and classifiable musculoskeletal impairments provides an opportunity for physiotherapists to assess, treat, and provide preventative education. **Materials and Methods:** We conducted a descriptive, retrospective chart review of all patients diagnosed with Hand-Arm Vibration Syndome at St. Michael's Hospital in the year 2007. Information was collected on patient demographics, nature of symptoms, musculoskeletal complaints, musculoskeletal diagnoses, co-existing conditions, and presence of risk factors. **Analysis:** Descriptve statistical analysis was conducted using SPSS version 16.0. **Results:** A total of 238 charts were reviewed. Greater than 50% of patients reported pain in the wrist, forearm, elbow, and shoulder. Tenderness in the upper extremity was experienced by 74% of patients, most commonly in the elbow (57%). Patients presented with various upper extremity musculoskeletal impairments, the most prevalent being shoulder/neck pathology (62%). Measurements of range of motion, stiffness, and strength impairments were not documented in 30% of the charts. **Conclusions:** Patients with Hand-Arm Vibration Syndrome experience pain, weakness, and range of motion restrictions throughout the upper extremity. Physiotherapists have a role in improving function and enhancing quality of life in this population. There is also a need for a standardized tool to improve assessment of musculoskeletal impairments in the upper extremity.

# Abstract: P005 – CanWell: A community partnership to deliver exercise programming for people with cancer

**Published:** 2010-07

**Authors:** O Cheifetz, F Serediuk, J Park Dorsay, G Hladysh, L Woodhouse

**Affiliations:** Hamilton Health Sciences, Oncology Program Henderson Campus, Ward F3, 711 Concession St., Hamilton, Ontario L8V 1C3, Canada; Hamilton Health Sciences, P.O. Box 2000, Sanatorium Road Southam Building, room 101, Chedoke Campus, Hamilton, ON L8N 3Z5, Canada; Hamilton Health Sciences, Rehabilitation Oncology—Ward 2, Henderson Campus, Hamilton, ON L8V 1C3, Canada; YMCA of Hamilton/Burlington/Brantford, Community Health Program Development, 356 Rymal Rd E., Hamilton, ON L9B 1C2, Canada; McMaster University, School of Rehabilitation Science Institute of Applied Health Sciences Rm. 442, 1400 Main St. West, Hamilton, ON L8S 1C7, Canada

**Learning Objectives and Session Content:**

1. Participants will gain an understanding of the process used, and key elements necessary, to establish a University-Hospital-YMCA partnership to successfully deliver safe and effective care for individuals with cancer in the community.
2. Participants will gain an understanding of the various roles and impact that physical therapists can have when they partner with an interprofessional team across agencies (from acute through to community-based care) to offer programs for individuals with cancer.
3. Participants will learn the importance of effective collaboration among administrators, kinesiologists, nurse practitioners, physical therapists and researchers when developing evidence-informed, community-based programs that involve inter-agency partnerships.
4. Participants will gain an understanding of the beneficial effects of individualized, supervised exercise training and education for individuals with cancer.
5. Participants will learn about the opportunities and challenges that exist when combining research programs with hospital/community programs.

This session will provide an overview of the process used and the key elements required for the formation of CanWell: A community-based exercise and education program for individuals with cancer. The presenters will discuss how to develop and implement evidence informed community-based, exercise program for people with cancer that are safe and effective. We will also present methods to evaluate the program strategies to maximize knowledge translation and health promotions into community programs. The importance of interprofessional and inter-agency partnering to deliver and evaluate this program will be discussed. Opportunities to expand the CanWell Program model of care to other communities allows people with cancer to engage in effective and safe exercise programs that are close to home.

**Relevance to the Physiotherapy Profession:** As survival continues to improve with advances in the medical management of individuals with cancer, physiotherapists are more likely to encounter cancer survivors within a variety of settings. Cancer has become a chronic disease, resulting in a shift towards managing the effects of cancer and it's treatment as a chronic disease. Collaborations between not-for-profit organizations (such as the YMCA), Universities, and physiotherapists provide opportunities to deliver much needed services in new frontiers. The expertise that physiotherapists have about acute care treatments, pathology and effects of exercise, positions us as natural leaders to develop collaborative, interprofessional teams to offer and support community-based programs. Collaboration with physiotherapists who are experts in rehabilitation research ensures that these programs are high quality, evidence-based, and continually evaluated to meet set standards. It is important that physiotherapists understand the value of collaborative programs that address the gaps in care and better serve the needs of cancer survivors, close to their home, as opposed to one-on-one individual treatments within traditional health care facilities. This model of community based health care represents a paradigm shift towards community care that is attractive to potential funders, demonstrates the professions' ability to work collaboratively in an inter-disciplinary setting, and better serves the complex needs of individuals living with cancer.

**Target Population:** This presentation will appeal to administrators, physical therapists who work in the public and private sector, physical therapy assistants, kinesiologists, researchers and those interested in program development and evaluation.

**Description of Supporting Evidence:** The evidence supporting increased physical activity with people who have cancer is overwhelming. The journal Seminars in Oncology Nursing was the first to dedicate a whole issue to this topic (Volume 23(4), 2007). In that issue Courneya and Friedenerich (2007) summarized the latest literature on the effects of physical activity and cancer control at all stages of cancer (pre-diagnosis, treatment phase, rehabilitation, and palliation). Other studies have demonstrated that increased physical activity can reduce the risk of breast and colon cancer (Friendenerich et al., 2002) and improve recovery following cancer treatment (Courneya et al., 2003 & 2005; Milne et al., 2007; Pinto et al,. 2005; Ohira et al., 2006). Increased physical activity level has been shown to be associated with improved cancer survival, and to prevent recurrence (Holmes et al., 2005; Meyerhardt et al., 2006). Although the supporting evidence is steadily growing on the benefits of exercise to offset the negative clinical sequelae of cancer and it's treatments, research on the value and safety of community-based exercise programs is limited and access to care providers with relevant expertise is scarce. The development of innovative, inter-agency, individualized, community-based, supervised exercise programs increases capacity and access to evidence-informed, safe, exercise programs for individuals with complex care needs. Physiotherapists with cancer related expertise should be active leaders in the development of community based exercise programs for people with cancer.

**Description of Session Format:** The session will consist of a series of speakers describing the process used and key elements that are essential to the development of CanWell: A community-based exercise and education program. Speakers will review the challenges and successes, understanding the impact of this type of program on the different partners, the importance of interprofessional and inter-agency partnering, evaluation of the program, and the value of consumer input in development of the program. A designated time for questions and answers will be at the end of the presentations, but ongoing dialog with the audience will be encouraged. In addition, video clips that provide and overview of the program and comments from the participants will be shown.

**Conclusions and Implications:** Partnerships between the YMCA, University, and an acute care hospital to develop a community based exercise programs, such as CanWell, demonstrates innovative thinking that can provide possible solutions to the ongoing financial challenges that continue to affect the health care system. It is important that physiotherapists understand their roles in developing and partnering with a variety agencies to provide sustainable exercise and education programs for individuals with cancer and their families.

# Abstract: P007 – Choices, benefits and risks related to physiotherapy treatment modalities for patients with past or present diagnosis of cancer

**Published:** 2010-07

**Authors:** MA Dalzell, S Shallwani

**Affiliations:** Jewish General Hospital—Segal Cancer Centre, 3755 Chemin de la Cote-Sainte-Catherine, Montreal, QC H3T 1E2, Canada

**Learning Objectives and Session Content:** The proposed session will provide a brief overview of cancer pathology, staging and treatment interventions with emphasis upon secondary neurological, musculoskeletal, and cardiovascular effects of the disease and its treatment. Chronic treatment-related fatigue, cancer cachexia, cardiac autonomic insufficiency, radiation fibrosis and bone metastasis will be reviewed. The benefits, risks, precautions, and contraindications to biophysical modalities, massage, traction, and exercise interventions will be discussed in interaction with the participants and reviewed in light of current research evidence.

Participants will be able to:

- identify disease-related and treatment-related musculoskeletal, neurological and cardio-respiratory changes related to the disease, chemotherapy and radiotherapy in patients with cancer
- screen for risks and determine the objectives of physiotherapy treatment interventions in this patient population
- determine the level of risk (precautions vs contraindications) associated with select treatment interventions ( thermal modalities, ultrasound, electrical stimulation, massage, exercise)
- choose appropriate treatment interventions dependant upon the stage of disease and treatment
- discuss the specific role of physiotherapy including exercise interventions in improving function for patients with cancer.

**Relevance to the Physiotherapy Profession:** As the rates of cancer incidence continue to increase, advancing medical treatments result in higher rates of cancer survivors suffering from chronic disability. Physiotherapists in a variety of clinical settings will encounter patients that present with musculoskeletal, neurological and/or cardio-respiratory dysfunction as a result of cancer disease or treatment. It is essential for physiotherapists to increase their knowledge on specific cancer-related issues and evidence-based practice in oncology in order to promote the improvement of function in this population. Through the performance of appropriate assessment techniques and skilled treatment interventions, physiotherapists have an important role to play in the management and rehabilitation of patients with cancer.

**Target Population:** This session will be particularly relevant for physiotherapists working in outpatient clinical settings. It will also be of interest to educators and clinical administrators responsible for risk management.

**Description of Supporting Evidence:** Many health-related quality of life and cross-sectional studies of functional status in different cancer populations have shown that patients are significantly impacted by short-term and long-term musculoskeletal, cardiovascular and neurological problems. Breast cancer patients continue to have chronic (greater than three years) residual upper limb disabilities, pulmonary fibrosis and dysfunction following surgery and radiotherapy. Gastrointestinal and lung cancer patients lose significant muscle mass (cachexia), have cardiac autonomic insufficiencies and experience long term fatigue. Head and neck cancer patients have postural problems and neck pain following radical and modified radical surgeries and radiotherapy to treat malignancies. Hematological cancer patients have chronic fatigue, as well as cardiovascular and neurological symptoms related to chemotherapy. These patients present with disease- and treatment-related issues, which could benefit from physiotherapy interventions. Randomized controlled trials have shown beneficial effects of specialized physiotherapy interventions on shoulder range of motion, lymphedema and upper extremity function in breast cancer patients. Non-invasive acupuncture, respiratory training, resistance and cardiovascular exercises on patients with lung cancer have also demonstrated significant improvement in performance status and symptoms of breathlessness and fatigue despite advancing disease. Systematic reviews and meta-analyses have discussed the benefits of exercise for patients with cancer for fatigue, physical performance, function and quality of life. These positive effects have been found across several types and stages of cancer, as well as across various phases of treatment. Pilot studies on the benefits of biophysical modalities and massage to reduce pain, nausea and radiation fibrosis in various cancer patient populations have recently been published. These findings reveal great potential for beneficial outcomes with physiotherapy interventions and exercise prescription in the management and rehabilitation of patients with cancer.

**Description of Session Format:** The session will include a 60 minute lecture presentation, followed by a 30 minute active engagement of participants in which 2 case studies will be discussed. Guidelines for the use of the different physiotherapy treatment modalities will be drafted by all participants in the last 30 minutes.

**Conclusions and Implications:** Cancer rehabilitation has not been integrated into the majority of university curricula despite recognition of the mounting needs of this patient population. Continuing education is essential to provide guidance to clinicians and ensure appropriate risk management strategies when planning treatment interventions. It is no longer adequate to assume that all treatment modalities are contraindicated and that the functional status of cancer patients can not be significantly improved with physiotherapy.

# Abstract: P011 – Evidence-informed exercise prescription

**Published:** 2010-07

**Authors:** RA Preuss

**Affiliations:** McGill University, School of Physical and Occupational Therapy, 3630 Promenade Sir-William-Osler, Montreal, QC, H3G 1Y5

**Learning Objectives and Session Content:** The session will include a review of current, evidence-informed guidelines for exercise prescription for cardiorespiratory (aerobic) exercise, resistance training (for muscle strength, power and endurance), and flexibility. The level of evidence supporting each recommendation will be reviewed, along with the basic science underlying the desired physiological adaptations. At the end of the session, the attendee will:

1. Be familiar with current evidence-informed guidelines for exercise prescription.
2. Be able to justify the use of these guidelines based on biomechanical and physiological principles, as well as evidence of clinical effectiveness.
3. Be able to design an evidence-informed exercise program to suit the specific needs of the individual client.

**Relevance to the Physiotherapy Profession:** Exercise prescription is one of the supporting pillars of physiotherapy practice, and as such it is essential that clinicians be informed and up to date on current evidence. This session will overlap two of the congress' themes—“Health Promotion” and “Merging Research and Practice”—by addressing the use of effective, evidence-informed exercise prescription, based on the most current research, to promote overall health and wellbeing and to optimize rehabilitation.

**Target Population:** The target audience for this session ranges from physiotherapy students to highly-experienced clinicians. For the student or new graduate, this session will provide a detailed overview of exercise prescription guidelines that can be adapted to most, if not all, adult patient populations. For the experienced clinician, this session will provide a review of fundamental concepts such as intensity thresholds, progressive overload, periodization, and training specificity, as well as a summary of the latest clinical evidence, allowing them to stay up-to-date on this ever evolving and rapidly expanding body of research.

**Description of Supporting Evidence:** This session will be based largely on published exercise guidelines, such as the position stands of the American College of Sports Medicine, and the evidence through which these guidelines have been derived. The primary focus will be on the effect of exercise and exercise recommendations in healthy adults, while making frequent reference to how these recommendations can be, and have been, adapted for specific populations such as older adults, or adults with hypertension, osteoarthritis, or chronic pain. Each recommendation will be accompanied by an “evidence category” ranging from “A” (consistent, supporting evidence from well designed, randomized controlled trials) to “D” (consensus agreement from a panel of recognized experts).

**Description of Session Format:** The session will be in lecture format, interspersed with interactive quizzes and the opportunity for discussion, with the intention of promoting active audience participation. The goal of the session will be to address two fundamental aspects of knowledge translation: synthesis and dissemination. The content of the session represents a synthesis of current evidence of exercise parameters designed to optimize effectiveness. The lecture format has been chosen as a means of disseminating this information as broadly as possible at a national gathering of physiotherapists.

**Conclusions and Implications:** Physiotherapists are expected to be experts in the design and prescription of exercises targeting cardiorespiratory and neuromusculoskeletal function. The current session is intended to provide a summary of current evidence to allow the attendees to remain at the forefront of evidence-informed exercise prescription, and to optimize the health and well-being of their clients.

# Abstract: P015 – Fibromyalgia: Movement for the trepidatious

**Published:** 2010-07

**Authors:** Debbie Patterson, Janice Ptak

**Affiliations:** PainSolutions Inc., 22 William Street, Sharon Ontario L0G 1V0; QEII Health Sciences Centre, Physiotherapy Department, Nova Scotia Rehabilitation Centre, 1341 Summer Street, Halifax, N.S., B3H 4K

**Learning Objectives and Session Content:** Upon completion of this symposium participants will:

1. Appraise emerging research on the pathophysiology of fibromyalgia.
2. Examine how these changes may affect the type and quantity of exercise prescribed.
3. Discuss methods for planning a personalized and holistic treatment protocol as well as exercise prescription guidelines.

**Session Content:** The session will provide an overview of recent research on the etiology of fibromyalgia. Emphasis will be on the clinical relevance and the clinical implications of these research findings. This should lead to best practice recommendations for physiotherapists working with patients who have fibromyalgia. An expanded paradigm for physiotherapy treatment will be presented, including not only exercise guidelines, but treatment strategies aimed at improving quality of life in this specific patient population. A case study will be presented, outlining assessment findings and treatment plan. The treatment plan will include education, exercise, and lifestyle changes. Outcome measures will also be presented.

**Relevance to the Physiotherapy Profession:** Fibromyalgia has long been misunderstood, and the confusion begins with nomenclature. There have been numerous name changes over time in attempts to recognize the causes and the etiology of the syndrome. Confusion and often skepticism accompanied this diagnosis as research proved or disproved various theories. Treatment was often inadequate, including physiotherapy. Research now indicates that fibromyalgia is a condition affected by both peripheral and central sensitization. These factors have profound effect on the treatment plan, and on the contribution of physiotherapy as a viable, conservative, evidence based treatment option. There is a significant amount of recent research in pain physiology and in the etiology of fibromyalgia which impacts the type of physiotherapy treatment, particularly exercises, prescribed for this population of people. Appropriate exercise, movement and activity could reduce pain and suffering in people with fibromyalgia, improve outcome measures and decrease health care and societal costs. Exercise programmes designed for other chronic pain condition have been perceived as less successful for fibromyalgia. Could this possibly be because they do not take important manifestations of this condition into consideration? Exercise prescription for fibromyalgia will not be successful unless it reflects the science that underlies its presentation, both in the musculoskeletal system, and in the central nervous system. Physiotherapists are often unaware of the tremendous influence that we can have on the central nervous system. Our treatment plans can affect both peripheral and central sensitization. It is well within the scope of physiotherapy practice to include in the treatment plan, strategies aimed at improving the quality of life. Physiotherapists are ideally suited to provide education about pain science and the condition of fibromyalgia. Sleep is a physiological behavior that physiotherapists can address, providing assistance.

**Target Population:** This presentation is appropriate for all physiotherapists, or physiotherapy support personnel in any clinical practice who have patients with fibromyalgia. It is also appropriate for physiotherapy students, or any health care professional with an interest in chronic pain conditions.

**Description of Supporting Evidence:** The Cochrane Database of Systematic Reviews 2007 Issue 4 published “Exercise for treating fibromyalgia syndrome:, Busch AJ, Barber KA, Overend Tj, Pelosa PMJ, Schachter CL. The author's conclusions are that there is “gold” level evidence that exercise programmes have a beneficial affect on fibromyalgia symptoms. Research on the long-term benefits of exercise for fibromyalgia is needed. There is increased evidence that abnormalities exist in skeletal muscles of patients with fibromyalgia (Le Goff et al, 2006, DeJong et al 2006). McIver et al 2005 demonstrated reduced nutritive flow response to aerobic exercise in fibromyalgia. There is mounting evidence to support the contribution of the Central Nervous System to the presentation of the signs and symptoms of fibromyalgia. For example, Cooke et al 2004 were able to use fMRI to show that more “neuromatrix” structures were activated in response to painful and non painful stimuli in fibromyalgia patients compared to healthy controls. McBeth et al 2005 described a dysregulation of the LHPA axis which drives the stress response. This is only some of the supporting evidence that demonstrates the complexities of fibromyalgia. It also points to the necessity to provide physiotherapy treatment that will take into account both peripheral and central sensitization. If physiotherapy is to be an evidence based treatment of choice for this condition, we must first be aware of the up to date science of fibromyalgia, and how that is clinically relevant in planning our treatment approach.

**Description of Session Format:** This symposium will be delivered by lecture supported by power point presentations. Questions, comments and information sharing will be encouraged.

**Conclusions and Implications:** In ensuring that physiotherapy becomes a first-line conservative and evidence based treatment choice for patients with this condition, it is important that physiotherapists continue to be informed of the most recent evidence in the field of fibromyalgia. This symposium will focus on translating research into clinically relevant information to improve both our treatment planning and treatment approach to patients with this persistent pain condition.

# Abstract: P016 – Implementing a community-based exercise program for people with neurological conditions: A step by step toolkit

**Published:** 2010-07

**Authors:** JA Howe, K Brunton, K Salisbury

**Affiliations:** Toronto Rehabilitation Institute, Patient Care—Education, Toronto, ON, M5G 2A2; University of Toronto, Dept of Physical Therapy, Toronto, ON M5G 1V7 Canada

**Learning Objectives and Session Content:**

1. To explore the partnership model used by a health care facility and a municipal recreation organization to implement a community-based exercise program.
2. To practise and learn the activities in the circuit program which are safe, do-able and functional exercises relevant to people with neurological conditions.
3. To consider and understand the factors necessary to maintain a safe program that is effective and valued by participants.

This session will describe the implementation of a community-based exercise program for people with neurological conditions and also present a Toolkit developed to assist physiotherapists to implement similar programs in their communities. The program is the outcome of a partnership between a municipal recreation organization and a health care facility. The strengths and assets of the two organizations were combined, for example, the exercise program is led by community centre fitness instructors but designed by the health care facility physiotherapists. A pilot program ran in the fall of 2007 which was evaluated for safety and participant outcome. At the time of writing, there have been 6 successful run-throughs of the program at two community centers from the fall of 2007 to the fall of 2009. The presentation will focus on three key elements of the Toolkit. The circuit training program with four levels of challenge at each exercise station and the rationale for the exercises will be presented. Second, the education and ongoing support for the fitness instructors will be covered. Third, with the program run off-site by fitness instructors, the multiple strategies for keeping the program safe and effective will be highlighted.

**Relevance to the Physiotherapy Profession:** People with neurological conditions such as stroke, acquired brain injury (ABI), and multiple sclerosis (MS) are at risk for inactivity and deconditioning after discharge from rehabilitation. To counteract the cycle of decline, ongoing access to physical activity and health promotion is essential. Transitioning to the community for these services would relieve pressure on the health care system and provide a sustainable solution for fitness maintenance. It is critical, however, that physiotherapists play a key role in the implementation of these community-based programs. Their expertise is necessary to develop the exercise program and provide education to the community centre fitness instructors about the movement issues of people with neurological conditions. Ongoing interaction and support of the fitness instructors by the physiotherapists is essential to the success of the model.

**Target Population:** This session will be of interest to a broad range of physiotherapy professionals including clinicians, managers, researchers, educators or policy-makers interested in health promotion for community-dwellers after formal rehabilitation is completed.

**Description of Supporting Evidence:** The disabling neurological impairments in persons with stroke, ABI and MS induce a sedentary lifestyle in these populations. Decreased amounts of physical activity may lead to deconditioning and further reductions in function. Evidence has shown that 40% of stroke patients experienced a decrease in mobility function within a year after discharge from rehabilitation (Paolucci, 2001). The circuit-training model is a form of group exercise that has been successfully used to improve several aspects of balance and mobility. In the circuit, clients rotate in round-robin fashion through stations of task-related exercises. Research on task-specific circuit training programs with the stroke population has demonstrated improvements in functional mobility, cardiorespiratory responses to exercise, strength in the paretic leg, walking endurance, balance, gait and stair climbing speed (Dean 2000, Eng 2003, Salbach 2004, Pang 2005, Marigold 2005, Sherrington 2008). Unfortunately the limited access to these programs after discharge from rehabilitation may reduce function and participation over time (Dean 2000). To slow or reverse the deconditioning cycle, partnering with community organizations (Rimmer 1999) could address the need for ongoing health promotion in a more cost-effective and sustainable manner.

**Description of Session Format:** The session will include lecture, small-group discussion, demonstration and practice of the circuit station exercises. The Toolkit is included in order to support ongoing learning for the attendees.

**Conclusions and Implications:** This program is the culmination of a process that began in the fall of 2005 with the recognition of a significant unmet need for exercise and health promotion in neurological populations after discharge from rehabilitation. The Toolkit will provide physiotherapists with the information and materials needed to implement a community-based exercise program to address this gap. The Toolkit describes a model of sustained partnership between health care and community recreation in order to ensure best practices in the program and most importantly to build capacity in fitness and exercise for our clients living in the community.

# Abstract: P022 – Physiotherapists role in the development of a women-only weight management program

**Published:** 2010-07

**Authors:** SK Sharma, DE Childerhose

**Affiliations:** Women's College Hospital, Women's Cardiovascular Health Initiative, Toronto, ON M5S 1B2, Canada

**Learning Objectives and Session Content:**

1. To discuss the unique role of a physiotherapist in a weight management program designed for women;
2. To discuss the theoretical framework behind the weight management program and how this program improves health attitudes, self-efficacy and intentions for positive behavioural changes; and
3. To review the various outcome measures and self-management principles used to evaluate the program.

**Relevance to the Physiotherapy Profession:** Healthy lifestyle behaviors are recommended for primary and secondary prevention of chronic diseases such as heart disease, diabetes and obesity. Physical activity is an essential component of a healthy lifestyle, contributing to both physical and mental health. It is important for those living with chronic diseases to learn how to integrate physical activity safely, considering their risks and barriers to exercise, some of which are unique to women. Physiotherapists play an important role in addressing physical challenges that may affect ones ability to become more active. Women specific issues such as incontinence, body image, and menopause can be addressed by a physiotherapist. The small group setting of our program allow women to discuss challenges and barriers with facilitators in a safe supportive environment. As we look more to self-management principles, it is important for physiotherapists to be able to integrate these principles and work to empower their patients to make long lasting behavior changes.

**Target Population:** This session will be of interest to clinicians and researchers who have a special interest in women's health and health promotion programs.

**Description of Supporting Evidence:** Behaviour change programs that are based on a theoretical model have been shown to improve health behaviours. The weight management program is theoretically grounded and guided by Bandura's Social Cognitive Theory(1986 & 1997). According to this theory, programs that are able to influence health attitudes and knowledge and increase the level of self-efficacy among participants will result in positive changes in their behavioural intentions and actions. Bandura suggests that having the knowledge concerning the benefits of healthy behaviours is not sufficient to elicit the behaviour, but that individuals must also and perhaps most importantly, believe in their own self-efficacy. In developing effective interventions to achieve a healthy weight Bandura identifies self-regulation to monitor an individual's behaviour. Effective self-regulation of health behaviours requires certain skills such as self-monitoring, setting realistic goals and evaluating progress. Physiotherapists are well positioned to help their clients learn these skills and to implement activities which are realistic and sustainable. The World Health Organizations Strategy on Diet, Physical Activity and Health recognizes the importance of improving diet and promoting physical activity to decrease deaths and disease burden. Strategies to follow this recommendation involve addressing risk factors for chronic disease, providing education to promote awareness of some of the modifiable risk factors, and implementing strategies for healthy lifestyle changes that are sustainable and comprehensive. Some of these risk factors and implementation strategies are unique to women. A small, women-only group setting provides a safe and supportive environment for members to share their experiences, successes and challenges for weight loss. As physiotherapists, we have the opportunity to guide these women in identifying strategies to overcome barriers and assist them in achieving their lifestyle goals.

**Description of Session Format:** This session will be a lecture format with the opportunity for an interactive discussion period regarding participants' experiences with women's health and weight management programs.

**Conclusions and Implications:** This session is aimed to illustrate the unique role for physiotherapy in women's health and in facilitated group programs with other health care professionals. It will illustrate how to use theoretical models to develop such programs and will review outcome measures used to track patient's progress.

# Abstract: P023 – Physiotherapy in a primary prevention program

**Published:** 2010-07

**Authors:** DE Childerhose, S Sharma

**Affiliations:** Women's College Hospital, Cardiology, Toronto, ON M5S 1B2, Canada

**Learning Objectives and Session Content:**

1. Discuss the role of physiotherapists in a primary prevention program designed for females living with high risk factors for developing cardiovascular disease (CVD).
2. Review the Women's Health Principles that guide management of co-morbidities in women, across the lifespan in a primary prevention setting.
3. Apply the Women's Health Principles to other areas of clinical practice by Physiotherapists.

**Relevance to the Physiotherapy Profession:** There is growing recognition of the significant burden of cardiovascular disease (CVD) in women. CVD risk factors are: cigarette smoking, abnormal ratio of lipid levels, high blood pressure, diabetes, abdominal obesity, stress, lack of daily consumption of fruit and vegetables, and lack of daily exercise. These represent 90% of the risk factors that can lead to a primary heart attack. Cardiac rehabilitation is an important intervention in the care of these patients with CVD. However, women with CVD still have significantly lower rates of participation and compliance in cardiac rehabilitation programs compared to their male counterparts. There are few primary prevention programs for cardiac health available in communities, whereas health care dollars are usually spent on secondary cardiac rehabilitation programs. Therefore, the development of primary prevention programs that cater to the needs of women are essential. Recent evidence suggests that rehabilitation programs modeled after the Women's Health Principles may better address the different needs and preferences of the high risk female population and improve outcomes. The multiple co-morbidities that exist with women attending a primary prevention program are a significant challenge to exercise prescription and compliance. The profession of physiotherapy is well suited to evaluate and treat these co-morbidities that this population faces. The Women's Health Principles are: 1) empowerment of women, 2) a broad definition of health, 3) collaborative planning, 4) accessibility, 5) high quality of care and 6) innovative and creative approaches to women's health care and research. Knowledge of Women's Health Principles, improves efficiency in attaining positive outcomes in female patients. Implementation of these principles from a physiotherapist's perspective, in a primary prevention program will be reviewed.

**Target Population:** This session will be of interest to a broad range of professionals including clinicians, managers and professional leaders of physiotherapy. In understanding that not all physiotherapists work in this unique setting, knowledge of the women's health principles and knowledge of how they can be implemented in other clinical settings will be outlined.

**Description of Supporting Evidence:** There is a shortage of primary prevention programs in health care across Canada. Health care dollars are spent in the tertiary care in Canada, yet there is worldwide knowledge, that through healthy living, diseases can be avoided or minimized. Many of these illnesses; ischaemic heart disease, hypertension and stroke, cancer, diabetes, obesity, and osteoporosis could be avoided or minimized if people were to adopt healthy lifestyles. Knowing this, it is crucial to move our focus towards lifestyle changes as a preventative strategy to avoid the onset of chronic conditions (Dean, 2008). The World Health Organization's global strategy on diet, physical activity and health focuses on increasing awareness and understanding of the influences of diet and physical activity on health and the positive impact of preventive interventions (WHO, 2009). However, this has proved to be difficult, and “effective measures are needed to increase people's opportunities and motivation to incorporate more physical activity into their daily lives” (Vuori, 2007). As physiotherapists, we have the opportunity to work with individuals to overcome physical and social barriers to increased activity with the goal of improved quality of life. Physiotherapists have basic knowledge with epidemiology and disease processes, that they are the ideal profession to work in a primary prevention program. Cardiovascular disease is the leading cause of death among Canadian women. However, women still identify as breast cancer as their major threat to health despite the fact that mortality rates for women from cardiovascular diseases is twice that from all cancers combined. Utilizing the Women's Health Principles has been shown to increase compliance and successful completion of a primary prevention program designed for women. Recent recommendations from the American Heart Association have focused on evidence-based guidelines for cardiovascular disease prevention in women. Using these guidelines, physiotherapists are well positioned in using their vast skill set to carry out primary prevention programs. With knowledge of exercise testing and prescription, musculo-skeletal and cardio respiratory assessment and treatment skills, and ability to empower patients, physiotherapists are the ideal health professional to lead primary prevention programs.

**Description of Session Format:** This session will be a lecture format, with the opportunity for leaded discussion through case study examples regarding experiences with women's health and cardiovascular disease.

**Conclusions and Implications:** This session is aimed to illustrate the unique role of physiotherapy in a primary prevention setting with a focus on cardiovascular disease and women's health. With the increase prevalence of CVD and associated risk factors, such as diabetes and obesity, physiotherapists are adequate positioned and knowledgeable to offer appropriate lifestyle interventions that will reduce disability and increase quality of life in women living with high risk factors for CVD.

# Abstract: P026 – Staying on your feet: Community based balance and education project aimed at fall prevention in older adults

**Published:** 2010-07

**Authors:** JA WalkerJohnston, JL Barnes

**Affiliations:** Community Services, Saskatoon City Hospital, Saskatoon Health Region, Saskatoon, Sask. S7L 0Z2

**Learning Objectives and Session Content:** A community based balance and education project aimed at fall prevention in older adults. This program was developed by the efforts of two individuals who came from different health professions, namely physiotherapy and recreational therapy to plan and implement a program aimed at fall reduction and increased awareness around falls.

**Learning Objectives:**

1. To discuss the need for fall prevention programs in the community and processes involved in fall screening and fall risk factor identification.
2. To discuss the benefits of partnerships in a community based setting along with incorporating best practise interventions.
3. To discuss the implementation and results of “Staying on your Feet”: programs.

**Session Content:** A four month community based balance and education project was piloted for older adults living in a senior's complex. The project consisted of thirty-one exercise classes and six education sessions. Exercise sessions were geared to improve the participants' overall balance by progressively working from individual muscle strengthening to balance work that incorporated multi-sensory activities provided in group or circuit work. Education sessions focused on fall risk factors. The objectives were to reduce falls in the facility by 10 percent, implement and evaluate a falls prevention balance and education program and to reduce the fear of falling among the participants. To evaluate the program, a variety of screening tools were utilized along with information gathering which will be discussed in terms of their effectiveness.

**Relevance to the Physiotherapy Profession:** There is strong evidence for multi-factorial population based approaches to fall prevention. Physical therapists but more importantly older adults are benefiting from partnerships between several health professions that focus on activities involved in health promotion, injury prevention and overall improvements in function and chronic disease management for many populations. The “Staying on your Feet” program piloted in Saskatoon, Saskatchewan was an example of partnering, collaboration and use of best practise interventions in fall prevention. The program was unique in that a community physical therapist worked along with a recreational therapist involved with a community older adult wellness program called Forever….in motion. Forever….in motion is a physical activity program that utilizes volunteers to lead community dwelling older adults in exercise sessions. These therapists worked together to develop the program including the evaluation. The physical therapist worked with a volunteer leader to offer the balance training portion to the program and the recreation therapist worked on the involvement of a variety of professions to assist with the education sessions around fall risk factors. Evaluation of the program proved that not only could falls could be reduced but balance confidence improved. By the completion of the program the number of fallers (the number of participants who had fallen previous to the program compared to the number of participants with falls at the end of the program) had been reduced by 27% and their balance confidence had improved on the average, by 9 %. The overall number of falls had also been reduced by 90% at the end of the program and 70% at the six month follow-up.

**Target Population:** This session will be of interest to a broad range of professionals as it is an example of not only a multidisciplinary but multi-factorial approach to fall prevention for the community dwelling older adult.

**Description of Supporting Evidence:** A comprehensive falls prevention model has been developed by the Canadian Falls Prevention Curriculum. This curriculum provides a format to provide consistent information on fall prevention activities, programs and research for healthcare providers. The Staying on Your Feet program was piloted with this curriculum in mind. Evidence exists that would support that not only community but population and multi-factorial based fall prevention programming.

**Description of Session Format:** This session will be a lecture format or special education session roughly one hour in length. It will be interactive in nature along with allowing for questions and discussion. Demonstrations and practise of tools will occur, as well as examples of balance exercises. Sharing of experiences related to other fall prevention initiatives will be encouraged.

**Conclusions and Implications:** The utilization of multi-factorial, community and population based approaches along with collaboration of community health care providers is a cost effective means, if not also a sign of the times in this environment of health care cuts, of promoting fall prevention and achieving behaviour change in older adults. The program exhibited overall success and set the stage for a proposal to request for funding to implement similar programs in existing older adults exercise programs called the Forever….in motion sites within the health region. The funding has been supported and work has begun in expanding the fall prevention programming throughout the health region to include not only the Forever….in motion exercise locations but a number of older adult interest groups that are open to learning more about fall prevention through a variety of agencies.

# Abstract: A011 – Physiotherapy models of service delivery, staffing, and caseloads: A profile of Level I Trauma Centres across Canada

**Published:** 2010-07

**Authors:** Megan E. Fisher, Martha N. Aristone, Katrina K. Young, Laurie E. Waechter, Michel D. Landry, Leslie A. Taylor, Nicole S. Cooper

**Affiliations:** an Assistant Professor in the Department of Physical Therapy at the University of Toronto; and an Adjunct Assistant Professor in the Gillings School of Global Public Health at the University of North Carolina at Chapel Hill (USA); the Professional Practice Leader at Sunnybrook Health Sciences Centre, and a lecturer at the University of Toronto

**Purpose/Objectives and Rationale:** To explore and describe physiotherapy models of service delivery, staffing, and caseloads in Level I trauma centres across Canada. **Relevance to the Physiotherapy Profession:** Current trends in trauma physiotherapy services are unknown, and the optimal practices cannot be ascertained until these trends are established. This study explored and described the physiotherapy services present in Canadian Level I trauma centres. **Materials and Methods:** A telephone questionnaire was created, tested, and administered to one experienced trauma physiotherapist at each of the 19 Level I trauma centres in Canada. **Analysis:** Quantitative data was analyzed descriptively for national trends using Statistical Package for the Social Sciences (SPSS) version 17.0.2 (SPSS Inc., Chicago, IL). **Results:** Data was collected from all 19 (100%) centres. Eighty-nine percent of the trauma centres provided physiotherapy services five days per week with priority weekend coverage. Physiotherapist Assistants (PTAs) were employed by 89% of centres and were utilized across the continuum of care. Those centres with PTAs appear to be more likely to provide patients with additional daily treatment. Departmental organizational structures were the most common (41%), and were associated with higher caseloads. Higher caseloads also appear to be linked with having less than ten years of experience as a physiotherapist. **Conclusions:** Variations exist between centres with regards to the delivery of physiotherapy services. These variations may result from differences in province-specific legislation, management of funding, as well as the lack of guidelines in the literature. Future research is needed to establish optimal models of physiotherapy services which are cost-effective and provide best patient care.

# Abstract: A013 – A standardized clinical coverage system for absences of physiotherapy staff in a program-managed environment

**Published:** 2010-07

**Authors:** S Wong

**Affiliations:** Physiotherapy, Baycrest, Toronto, ON Canada; University of Toronto, Toronto, ON Canada.

**Purpose/Objectives and Rationale:** To develop a system to manage clinical coverage during absences of physiotherapy staff in a program-managed environment. **Relevance to the Physiotherapy Profession:** De-centralized staffing in program management presents challenges for clinical coverage during absences of physiotherapy staff. A standardized system for clinical coverage communicating clear expectations and service provision to physiotherapists and managers facilitates planning and care. **Description:** Members of the Professional Practice Network of Ontario and Physiotherapy Academic Practice Leaders Group (University of Toronto) were surveyed. Input from responses (*n *= 19) informed the development of the clinical coverage system. Four components were developed: 1) time off request process involving staff, program managers and practice leader, 2) program-specific physiotherapy coverage identifying time frames and types of services to be provided, 3) physiotherapy cross-program support, incorporating skill mix, nature of practice and staffing levels in each program, and 4) standards for documentation during clinical coverage. **Observations:** Critical factors for success include: 1) uniform approach in application of new system/process across all programs, 2) timely access by staff to information related to absences via a network drive, 3) managers' understanding of physiotherapy scope of practice and services within program(s), and 4) consensus on expectations and services provided during staff absences. **Critical Assessment:** Using a standardized clinical coverage system to manage staff absences reduces conflicts and inconsistencies and facilitates the coordination of services especially during peak vacation time. **Conclusions:** Staff absences in a program-managed environment challenge adequate and efficient clinical coverage. A standardized coverage system communicates clear expectations on service provision to physiotherapists and managers, enhancing collaboration and understanding; and ensuring adequate coverage to patient care.

# Abstract: A014 – The optimization of the full extended physiotherapy scope of practice in the Canadian Forces: A success story

**Published:** 2010-07

**Authors:** Luc J. Hébert, Peter Rowe, Kelly Debouter

**Affiliations:** (1) CFHS GP HQ, D Med Pol, Physiotherapy, Ottawa (Ont); (2) Laval University, Quebec (Qc), Canada; (3) 1 Field Amb, CFB Edmonton (AB), Canada

**Purpose/Objectives and Rationale:** The Canadian Forces physiotherapy services are implementing a standardized model of physiotherapy practice that is embedded in the Canadian Forces Primary Care Renewal Initiative Model, which is a patient-centered model focusing on collaborative practice. **Relevance to the Physiotherapy Profession:** The implementation of such a collaborative practice model requires the use of an extended physiotherapy scope of practice and a specific training that must be clearly identified in terms of required physiotherapy knowledge, skills, and attitudes. **Description:** The unique characteristics of this new model include: a. national standards of care and practice; b. a practice focusing on prevention, early intervention, and direct access; c. an extensive clinical preceptorship/mentoring; and d. an active professional communication network. **Observations:** The implementation of this new model has posed several challenges such as the education of both patients and health care providers (HCPs), an extended period of mentoring for all physiotherapists, and the need for all to accept that shift of the physiotherapy paradigm from a curative to a wellness model of practice. **Critical Assessment:** This new practice confirms physiotherapy as a true primary care provider profession, which requires optimizing the physiotherapist's extended scope of practice. This model cannot be implemented using the same format at all clinics. When assessing the readiness of physiotherapists to use this model, a distinction had to be made between competency and proficiency, two variables that must not be confounded. **Conclusions:** The implementation of this innovative physiotherapy delivery model has set a new standard of excellence that is now requested by all HCPs and especially our physician's colleagues.

# Abstract: A017 – Physiotherapists and electronic documentation: The love-hate relationship

**Published:** 2010-07

**Authors:** Denise Lai

**Affiliations:** Physiotherapy Professional Practice Leader, Dept. of Rehabilitation, Mount Sinai Hospital, Toronto; Lecturer, Dept. of Physical Therapy, University of Toronto.

**Purpose/Objectives and Rationale:** 1) Identify challenges, enablers and recommendations for electronic documentation (EDOC) from physiotherapists; 2) Compare perceptions between regular and occasional EDOC users. **Relevance to the Physiotherapy Profession:** EDOC is becoming the new norm of practice for physiotherapists in Canadian hospitals. Staff feedback is critical in driving system changes. **Materials and Methods:** All physiotherapists at Mount Sinai Hospital were invited to participate. Four focus groups were conducted by an experienced and neutral facilitator. Sessions were audiotaped and transcribed verbatim. **Analysis:** Each transcript was analyzed using an open-coding technique to identify themes, into which data was then categorized. **Results:** Nineteen physiotherapists—12 regular and 7 infrequent users—participated. Four themes emerged: 1) System and Functionality; 2) Quality; 3) Perceptions, and 4) Recommendations. The main system enabler identified was easy and timely access to charts. The primary barrier was the continued existence of both paper and online charting, which contributes to confusion and inefficiency. Quality of electronic documentation was felt to be negatively impacted by repetition and fragmentation of information, lack of details and inconsistency in where staff input information. Interprofessional communication, however, was not felt to be compromised. Although EDOC was perceived as being less efficient than paper charting, staff was overall satisfied with it. Infrequent users felt there was more learning curve, but were more satisfied. The top recommendation was to have one documentation system for all hospital staff and services. **Conclusions:** There are challenges for staff to perform electronic documentation and a need to improve quality of EDOC. Information from this study will help identify areas of improvements to augment staff satisfaction in EDOC.

# Abstract: A022 – Factors affecting recruitment and retention of rehabilitation professionals in Northern Ontario

**Published:** 2010-07

**Authors:** CS Winn, BA Chisholm, JA Hummelbrunner

**Affiliations:** Northern Studies Stream and Rehabilitation Studies Program, Northern Ontario School of Medicine, 955 Oliver Road, Thunder Bay, ON, P7B 5E1

**Purpose/Objectives and Rationale:** An objective of the Rehabilitation Studies and Northern Studies Stream Programs is to facilitate the recruitment and retention of physiotherapists in Northern Ontario. The purpose of this study was to determine the factors that led to practicing rehabilitation providers moving to and subsequently choosing to stay in the region. **Relevance to the Physiotherapy Profession:** This study will assist health providers and funders to more effectively address recruitment and retention of physiotherapists to underserviced areas. **Materials and Methods:** 639 rehabilitation professionals currently working in Northern Ontario were invited to participate in this study. Participants voluntarily completed an electronic survey in which they answered several demographic questions and rated factors affecting their decision to move to and to stay in Northern Ontario. Data was collected through the Survey Monkey website with results exported for statistical analysis. **Analysis:** Descriptive statistics were used to analyze the demographics of the study sample. Correlational statistics were used to determine the strength of the relationship between demographic and self-identified factors and an individual's likelihood to move to and to stay in Northern Ontario to work as a rehabilitation professional. **Results:** The results of this survey clearly demonstrated the importance and positive influence of family ties, past personal experiences in the region, completion of an academic and/or clinical education experience and of continuing professional development opportunities, in choosing to move to and to stay in Northern Ontario. **Conclusions:** This research supports the need for professional and career development of rehabilitation professionals and for ongoing academic and clinical education in Northern Ontario as a recruitment and retention strategy.

# Abstract: A023 – Shoulder soft tissue injuries: a collaborative management model

**Published:** 2010-07

**Authors:** Pamela Wasson

**Affiliations:** WorkSafeNB

**Purpose/Objectives and Rationale:** To develop a care map for injured workers with Soft Tissue Injuries (STI's) of the Shoulder in order to reduce claim duration while maintaining return to work success. **Relevance to the Physiotherapy Profession:** Physiotherapists are recognized as experts for STI's. **Description:** Seven physiotherapists and four orthopaedic surgeons were involved in developing a care map and treatment protocol. The physiotherapist performs a comprehensive assessment, then determines a treatment stream; Rehabilitation Stream (likely no need for specialist intervention), Direct Referral Stream (need specialist, likely surgical), or Concurrent Stream (may benefit from rehabilitation, may also need injection or surgery). The physiotherapist determines the need for specialist intervention. The surgeon reviews the physiotherapy assessment and recommendation, refers for appropriate imaging, and schedules an expedited consultation. **Observations:** A recent review revealed that clients with shoulder STI's were successful in returning to their pre-accident work, however were off work for up to 24 months. A major delay was waiting for investigations and consultations without clear clinical indications. Physiotherapy practice varied among providers. Surgeons' practice with respect to surgery was also variable. Clients who had surgery with only soft surgical indications had poorer functional outcomes. **Critical Assessment:** We proposed to streamline service to ensure that clients who were likely to need orthopaedic intervention had timely access, and clients who did not, received evidence-based physiotherapy. Preliminary results show good compliance with the care map and positive feedback from the referral source and clinicians. **Conclusions:** This is a new model of collaborative management where physiotherapists provide triage for orthopaedic consultation and provide the rehabilitation of shoulder STI's.

# Abstract: A024 – System level evaluation of the ACPAC (Advanced Clinician Practitioner in Arthritis Care) trained practitioner in Ontario

**Published:** 2010-07

**Authors:** L Passalent, L Soever, C Kennedy, K Warmington, R Shupak, R Thomas, S Linekar, K Lundon

**Affiliations:** Mobility Program Clinical Research Unit, St Michael's Hospital, Toronto,ON M5B 1W8 and Department of Physical Therapy, University of Toronto, Toronto, ON M5G 1V7; Department of Rehabilitation, Mount Sinai Hospital, Joseph and Wolf Lebovic Health Complex, Toronto ON M5G 1X5 and Department of Physical Therapy, University of Toronto, Toronto ON M5G 1V7; Mobility Program Clinical Research Unit, St Michael's Hospital, Toronto, ON M5B 1W8; Mobility Program Clinical Research Unit, St Michael's Hospital, Toronto, ON M5B 1W8; The Martin Family Centre for Arthritis Care and Research, St Michael's Hospital, Toronto, ON M5B 1W8; Mobility Program Clinical Research Unit, St Michael's Hospital, Toronto, ON M5B 1W8; The Arthritis Society, Toronto, ON M5G 1E6; Schneider R; Division of Rheumatology, Hospital for Sick Children, Toronto, ON M5G 1X8; ACPAC Program, St Michael's Hospital, ON M5B 1W8

**Purpose/Objective and Rationale:** The purpose of this presentation is to describe the methodological framework chosen to evaluate the impact of the Advanced Clinician Practitioner in Arthritis Care (ACPAC) program graduates on the Ontario health care system. **Relevance to the Physiotherapy Profession:** The aim of the ACPAC program is to prepare experienced physical and occupational therapists for expanded scope roles and to develop innovative models of arthritis care. It is now critical to evaluate the extent to which the ACPAC program graduates have developed and integrated new models of arthritis care throughout the province of Ontario. **Description:** In Ontario, the Hospital Report: Rehabilitation Series has derived four quadrants for evaluation using a Balanced Scorecard approach. This adapted model will frame the evaluation and will include the following quadrants: Client and Stakeholder Perspectives; Clinical Utilization and Outcomes; System Integration and Change and Financial Performance and Condition. Each quadrant will use a variety of qualitative and quantitative primary data collection methods throughout 2 fiscal years (2009/2010 and 2010/2011). **Observations:** Results from the above evaluation will be used to guide continuous performance improvement of the ACPAC graduates with respect to their patient, organization and system level impact. **Critical Assessment:** This evaluation will inform ACPAC stakeholders about internal processes and external outcomes at the patient, organization and system levels to guide continuous performance improvement of the ACPAC graduates and their respective programs. **Conclusions:** Completion of this evaluation will determine the impact of the ACPAC program graduates on various levels of the Ontario healthcare system and ensure the sustainability of this new human health resource for the future.

# Abstract: A031 – Taking issue with community rehabilitation: Results of a provincial rehabilitation needs and gaps assessment

**Published:** 2010-07

**Authors:** Michelle Ploughman, Dale Morgan, Larry Kelly

**Affiliations:** Eastern Health, Post-Doctoral Fellow, Primary Health Research Unit; Rehabilitation Program, Eastern Health; Rehabilitation Program, Eastern Health; L.A. Miller Centre, 100 Forest Rd. St. John's NL A1A 1E5

**Purpose/Objectives and Rationale:** Eastern Health (serving 260,000) identified the need for rehabilitation services evaluation. The evaluation answered the question, “For adults living within the boundaries of Eastern Health, as well as within Newfoundland and Labrador, how well are rehabilitation needs being met across the continuum of care?” **Relevance to the Physiotherapy Profession:** An aging population, emphasis on chronic disease management and move toward community living for people with disabilities requires the re-evaluation of current adult rehabilitation services. Findings will be of interest to leaders, managers and policy-makers in physiotherapy and other rehabilitation disciplines. **Materials and Methods:** Guided by steering and advisory committees, we used multiple methods including evaluation of grey literature and satisfaction surveys, health indicators, electronic and postal surveys for rehabilitation providers (*n *= 204) and patients and families (*n *= 266) in Eastern Health, as well as focus groups (*n *= 12), and key informant interviews (*n *= 24) in all regions of the province. **Analysis:** Surveys were analysed descriptively using Excel. Focus group and interview text themes were coded and analysed using NVIVO8 followed by cohesion of themes. **Results:** The primary concern of both providers and patients was the lack of home/community supports and follow-up. Lack of these services impeded discharge from hospital and stimulated unnecessary emergency room visits. Furthermore, respondents identified gaps in rehabilitation services for people with chronic disease and special populations (bariatric, young adults). Focus groups and interviews revealed a strong rural-urban split in accessibility of rehabilitation services. **Conclusions:** Although policy makers often emphasize improvements within institutions, our results show that front-line providers, managers, patients and families perceive that rehabilitative care should be improved much closer to home.

# Abstract: A036 – Relative attractiveness of employment settings for physical therapists (1999–2007): Assessing ‘stickiness’ and ‘inflow’ across the care continuum in Ontario, Canada

**Published:** 2010-07

**Authors:** Michel D. Landry, Robyn Hastie, Raisa B. Deber, Molly C. Verrier

**Affiliations:** (Department of Physical Therapy and Graduate Department of Rehabilitation Science in the Faculty of Medicine, at the University of Toronto, Toronto, Ontario, Canada); (Department of Health Policy, Management and Evaluation (HPME) in the Faculty of Medicine, at the University of Toronto, Toronto, Ontario, Canada); (Department of Health Policy, Management and Evaluation (HPME) in the Faculty of Medicine, at the University of Toronto, Toronto, Ontario, Canada); (Department of Physical Therapy and Graduate Department of Rehabilitation Science in the Faculty of Medicine, at the University of Toronto, Toronto, Ontario, Canada) 4 Department of Health Policy, Management and Evaluation (HPME) in the Faculty of Medicine, at the University of Toronto, Toronto, Ontario, Canada)

**Purpose/Objectives and Rationale:** Health human resources ranks as a high priority across Canada. The objectives of this study were to assess changes in health human resources for physical therapists (PTs) in the Province of Ontario, and to examine the relative attractiveness of practice settings between 1999 and 2007. **Relevance to the Physiotherapy Profession:** Despite the growing dimensions of the rehabilitation workforce, shortages of PTs are consistently reported across most jurisdictions. As the health reforms continue to shift the focus of care from hospital to the community, it is critical to understand the extent to which traditional and emerging practice settings are able to retain PTs. **Materials and Methods:** A longitudinal dataset of registered PTs in Ontario (1999–2007) was created, and primary employment was categorized as hospital, community, long term care (LTC) or other sectors. **Analysis:** ‘Inflow’ was defined as the number of new PTs in each practice setting, and ‘stickiness’ was defined as the transitional probability that a PT will remain in the same setting year-to-year. Values were then calculated for each sector, and trends over time were analyzed. **Results:** The hospital sector had the highest stickiness indicating that it was an attractive sector, however due to relatively low inflow, it also had the lowest expansion rate. The community and LTC care sectors had the highest inflow, but the lowest stickiness indicating they were the least attractive employment sectors. **Conclusions:** Health reforms are incrementally shifting care settings. Our results highlight that these emerging sectors may be relatively unattractive from a workforce perspective.

# Abstract: A040 – Physiotherapists: Captains of the inpatient rehabilitation ship

**Published:** 2010-07

**Authors:** L O'Callaghan, E Alexander

**Affiliations:** St.Joseph's Health Centre—Toronto Rehabiliation Institute—Hillcrest Site, Toronto, ON, M6R 1B5

**Purpose/Objectives and Rationale:** To examine the key characteristics that makes physiotherapists excellent admission/service coordinators. **Relevance to the Physiotherapy Profession:** Physiotherapists possess the skills and knowledge to advance positions within inpatient rehabilitation facilities. **Description:** Service coordinators are the primary contacts for referral intake at many rehabilitation facilities. Service coordinators review applications and determine if the patient presents with attainable goals for the timeframe allowed by the given program. They are in constant communication with referring facilities and are the primary liaison between referring facilities and the in-patient rehab team. An informal survey of the service coordinators employed by Toronto Rehabilitation Institute (TRI); including discipline, work experience, years as a service coordinator, and top three skills required to succeed as a service coordinator; was undertaken. **Observations:** Over 80% of respondents were physiotherapists. Number of years employed as a service coordinator ranged from .25–16+ years. Work experience varied, however greater than 65% of respondents had acute care experience. Recurrent themes observed when skills required to success were examined include: communication/interpersonal skills (including mediation, relationship building and customer relations); background and work experience that assisted in determining rehab readiness and provided an understanding of the patient care continuum; critical thinking; and organization. **Critical Assessment:** Communication/ Interpersonal skills was identified as a success factor by all respondents. Interpersonal/communication skills, varied practical experience, and knowledge of rehab readiness are attributes physiotherapists develop through their educational and work experiences. **Conclusions:** Physiotherapists possess the necessary skills to advance their position within rehabilitation facilities and make excellent candidates to take the helm and be the captains of rehab ships.

# Abstract: A042 – Bringing accountability and passion to waitlist management

**Published:** 2010-07

**Authors:** D Titus, P MacDougall

**Affiliations:** Capital Health, Queen Elizabeth II Health Sciences Centre

**Purpose/Objectives and Rationale:** To present a comprehensive, multi-faceted approach to Outpatient Physiotherapy waitlist management lead by frontline physiotherapy staff. **Relevance to the Physiotherapy Profession:** Access to public outpatient physiotherapy services is limited. Increased referral rates and population needs for the service are not matched by the resources, requiring a critical look at service provision. **Description:** Frontline staff met together to come up with a plan to improve patient access, streamline interventions and provide patients with tools to self-manage in the community. The team included Physiotherapists, Physiotherapy Assistants and clerical; all were encouraged to participate in the process. Staff was empowered to make decisions with minimal management intervention. The team engaged Family Physicians to help support patient self-management. **Observations:** Staff's passion and commitment to provide timely, appropriate intervention reflective of current best practice resulted in improved access and services to patients on the outpatient physiotherapy waitlist. **Critical Assessment:** Objectively the following results were noted: -End of month waitlist decreased even though the number of new referrals continues to increase. -The average number of new patients seen per month has increased by 46 %. -The average wait-time is 50% less than a year ago. **Conclusions:** The empowerment of the team and the willingness to change practice were crucial to the success of the frontline waitlist management approach. The initiatives may be adapted to meet the demand and challenges of providing physiotherapy patient-centered care in the ever-evolving health care environment.

# Abstract: A045 – Creative Knowledge Translation Strategy: Partnership between the university, professional association and clinical practice to develop a shared knowledge broker position

**Published:** 2010-07

**Authors:** AM Hoens, SJ Garland, R Tunnacliffe

**Affiliations:** Department of Physical Therapy, University of British Columbia & Physiotherapy Assocation of BC; 212–2177 Wesbrook Mall, Vancouver, BC V6T 1Z3

**Purpose/Objectives and Rationale:** Although there is a well recognized need to enhance knowledge translation in PT, there are limited resources within the university, professional association and clinical practice domains. Thus, a creative solution, using shared funding to create a knowledge broker position was developed. **Relevance to the Physiotherapy Profession:** The literature is replete with evidence demonstrating deficiencies in effective knowledge translation in PT. Creating a funding partnership enables fiscal ability, access to complementary resources and synchronization of focussed objectives. **Description:** Sharing funding from the BC Rehabilitation Sciences Research Network (UBC Department of PT and local health organizations) and the Physiotherapy Association of BC created an appointment for 0.4 FTE for 1 year with 1 year renewable. Goals and deliverables that addressed the needs of each funding partner and selected shared objectives were pursued. **Observations:** An initial needs assessment was undertaken to inform the goals and deliverables. Evaluation via feedback from funding partners, faculty and project participants was performed. **Critical Assessment:** Evaluation of the role highlighted the following: Benefits: ability to respond/create/explore partnerships; sharing of resources, linkages and exchanges, dedicated time & resources; participation by spectrum of stakeholders, attractive to granting agencies & the opportunity to use leaning from implementation science. **Challenges:** No template, limited FTE, navigation of multiple organizations and overcoming preconceived barriers. **Conclusions:** Shared funding partnerships from academic, administrative, clinical and professional organization domains is an attractive model to achieve what none could do alone.

# Abstract: A046 – The Advanced Clinician Practitioner in Arthritis Care (ACPAC) Program: Impact on community practice (A pilot study)

**Published:** 2010-07

**Authors:** S Lineker, N Varatharasan, K Lundon, R Shupak, R Schneider, C MacKay

**Affiliations:** The Arthritis Society, Toronto,ON, M5G 1E6, Canada; St. Michael's Hospital, Toronto, ON, M5B 1W8, Canada; The Hospital for Sick Children, Toronto, ON, M5G 1X8, Canada; Arthritis Community Research and Evaluation Unit, Toronto, ON, M5T 2S8, Canada

**Purpose/Objectives and Rationale:** An advanced practice practitioner (APP) training program was developed for physiotherapists (PTs) and occupational therapists (OTs) in response to the need for an interdisciplinary approach to efficiently manage patients with arthritis. This pilot project compared practice between program graduates and non-APPs working in the community. **Relevance to the Physiotherapy Profession:** This project addresses the leadership in action stream and describes potential roles for APPs in the community and identifies variables important in the design of future studies. **Materials and Methods:** Charts of adult clients with suspected or confirmed inflammatory arthritis were randomly selected for review by trained reviewers using standardized data extraction forms (*n *= 60). **Analysis:** Independent t-tests were used to compare APP and non-APP client demographics, disease and referral characteristics, interventions and outcomes. **Results:** Compared to non-APPS clients (*n *= 29), more APP clients (*n *= 29) lived in rural communities, were referred specifically for assessments and had moderate disease (*p *≤ 0.04). APPs were more likely to see clients without a confirmed diagnosis and document comorbidities, morning stiffness and grip strength (*p *≤ 0.05). APPs were more likely to advocate with the client's family, physician or specialist and recommend or provide exercise (*p *≤ 0.04). Non-APPs saw more clients with severe disease and provided or recommended more education about community resources and assistive devices (*p *≤ 0.05). **Conclusions:** The population of clients seen by APPs was different than that seen by non-APPs working in the community. APPs may have an important role in providing care to clients in rural communities and in assessing and advocating for those with diagnostic uncertainty and comorbidities.

# Abstract: A049 – Kotter's model: A tool for clinical education

**Published:** 2010-07

**Authors:** SM Murphy

**Affiliations:** Department of Physical Therapy, Vancouver BC V6T 1Z3

**Purpose/Objectives and Rationale:** Kotter's model is cited in organizational development literature as an effective model of transformational change, however information describing its use in clinical education is sparse. This presentation describes use of the model in a physical therapy clinical education program. **Relevance to the Physiotherapy Profession:** Transformational change is needed in clinical education programmes due to the need for increased capacity for clinical learning opportunities and the need to adapt to changing health care models. **Description:** Kotter's model consists of 8 steps. The first three steps (establishing urgency, creating a guiding coalition, and creating a vision) were achieved by holding a summit symposium. The next steps (communicating the vision, empowering others, and creating short term “wins”) were achieved using strategies to facilitate stakeholder involvement. The final two steps (consolidating the improvements and institutionalizing the changes) are now complete. **Observations:** The UBC clinical education program has achieved significant revitalization and increased stakeholder involvement. Kotter's third step of creating a vision was key in engaging the clinical community and resulted in an increased number diversity of placements. Consolidating the improvements and institutionalizing the changes proved the most challenging steps to achieve. **Critical Assessment:** Survey evaluation of the clinical education program by students and clinical educators has shown a high level of satisfaction with the revised program. Enhanced engagement with the clinical community has been maintained through a provincial advisory committee, a newsletter, and an updated website. **Conclusions:** Kotter's model can be an effective tool when considering clinical education program renewal. It provides a guiding framework for both strategic planning and operationalisation of change.

# Abstract: A050 – Evaluation of an educational video on airway clearance techniques for patients with cystic fibrosis

**Published:** 2010-07

**Authors:** Kenneth Wu, Patricia Fox, Elizabeth Tullis, Anne L. Stephenson

**Affiliations:** (1) Adult Cystic Fibrosis Program, St. Michael's Hospital, Toronto, ON, Canada; (2) University of Toronto, Toronto, ON, Canada; (3) Mobility Program Clinical Research Unit, Keenan Research Centre, Li Ka Shing Knowledge Institute, St. Michael's Hospital, Toronto, ON, Canada; (4) Keenan Research Centre, Li Ka Shing Knowledge Institute, St. Michael's Hospital, Toronto, ON, Canada

**Purpose/Objectives and Rationale:** Chest physiotherapy (CPT) is vital in facilitating airway clearance in cystic fibrosis (CF). Nonadherence to CPT is well recognized in the literature. A video entitled “Breathe Easier: Airway Clearance Techniques (ACTs) for People with CF” was produced. This study evaluates the effectiveness of this video in enhancing patients' knowledge and intention towards performing regular CPT. **Relevance to the Physiotherapy Profession:** Education provided by physiotherapists in the clinic may help enhance patient's treatment adherence. The limited resources have restricted the number of patients seen and time spent per patient in the clinic. This video helps provide part of the education during patient's clinic visit. **Materials and Methods:** A prospective cohort study design was used. Sixty individuals attending the CF Clinic were recruited. The video was evaluated using the theory of planned behaviour. The primary outcome measure was behavioural intention. Secondary outcome measures included behavioural beliefs, attitude towards behaviour, subjective norm, perceived behavioural control, and other knowledge related variables. Patients were asked to fill out a questionnaire, view the video, and complete a post-video questionnaire. **Analysis:** Descriptive statistics were carried out. A paired t-test was used to compare responses before and after viewing the video. **Results:** Patient's intention to do regular CPT increased significantly after watching the video (*p *< .01). Ninety-three percent of patients reported hearing about and/or seeing examples of ACT for the first time from the video. Watching the video stimulated questions regarding CPT in 28% of patients. **Conclusions:** Patients had stronger intention to perform regular CPT after viewing the video. The educational value of the video was demonstrated in this study.

# Abstract: A051 – Physical therapists working in expanded roles in Orthopaedic Clinics: Impact on non-surgical patients

**Published:** 2010-07

**Authors:** C MacKay, AM Davis, N Mahomed, EM Badley

**Affiliations:** Toronto Western Research Institute, Toronto, Ontario, Canada and University of Toronto, Toronto, Ontario, Canada

**Purpose/Objectives and Rationale:** Advanced Practice physiotherapists (APPs), are increasingly working in orthopaedic clinics to expedite access to specialist care and support comprehensive disease management. The primary objective was to determine the short-term effects of APP consultation on exercise and self-efficacy in patients with hip or knee arthritis who weren't recommended surgery. **Relevance to the Physiotherapy Profession:** This model of care (MOC) has potential to improve outcomes in non-surgical patients. To date, research examining the impact of the APP MOC on non-surgical patients is limited. **Materials and Methods:** Patients with hip or knee arthritis seeing an APP at an orthopaedic clinic who were assessed as not yet needing surgery completed questionnaires at their clinic visit (demographics, adapted Stanford Exercise Behaviour Scale, Chronic Disease Self-efficacy Scale). A structured telephone interview was conducted after 6 weeks. **Analysis:** Descriptive statistics, paired t-tests and effect sizes were examined. **Results:** 73 patients participated in the study. Seventy one percent (52/73) of patients reported that the APP recommended exercise during their clinic visit. Of these, 83% (*n *= 43) reported using exercise to manage their arthritis since the visit. Almost 50% reported an increase in time spent stretching (32/65) and over 40% reported an increase in time spent walking (28/66) or doing strengthening exercises (26/64) at follow-up. Mean chronic disease self-efficacy scores significantly improved at follow-up (*p *< 0.001). Common barriers to arthritis management were time, cost and other health problems. **Conclusions:** The results suggest that an APP consultation increases exercise in some non-surgical patients and improves chronic disease self-efficacy. Further research is needed to understand long term outcomes.

# Abstract: A055 – Development of the physiotherapy role in pediatric eating disorders: Taking action in mental health with a Best Practice Approach

**Published:** 2010-07

**Authors:** Lisa Scott

**Affiliations:** McMaster Children's Hospital, Pediatric Eating Disorders Program, Hamilton, ON L8N 3Z5, Canada

**Purpose/Objectives and Rationale:** To develop and implement the role of the physiotherapist within the Pediatric Eating Disorders Program, McMaster Children's Hospital, Hamilton, Ontario. There is evidence that interventions within the clinical domain of physiotherapy can be beneficial. **Relevance to the Physiotherapy Profession:** The benefits of using exercise in mental health are well documented. However, the use of physiotherapists to promote, prescribe and manage exercise with children and youth with eating disorders is not widely practiced in North America. **Description:** A review of relevant literature using the following search terms; Eating Disorders (Anorexia Nervosa, Bulimia Nervosa, Binge Eating), Exercise (Physical Activity), Physiotherapy, Body Awareness and Yoga was done. Databases were searched up to October 2009. Position statements on exercise and physical activity from the World Confederation for Physical Therapy, the Canadian Physiotherapy Association, and the American Physical Therapy Association were utilized. Interventions were used with pediatric eating disorder inpatients, outpatients, females and males, aged 9–17 for eight months. **Observations:** Patients, families and multidisciplinary team members valued the role. Patients examined their relationship with exercise. Patients practiced and developed an understanding of healthy exercise and greater body awareness. Patients showed progress with goals; they moved from supervised exercise to practicing prescribed exercise independently. **Critical Assessment:** We have not gathered sufficient evidence to conclude that it optimizes patient health or outcomes. **Conclusions:** Based on our early observations, it appears there is value having the physiotherapist manage the eating disorder patients relationship with exercise. This informal result merits further investigation through a research study, in order to clarify the role of the physiotherapist in pediatric eating disorders.

# Abstract: A058 – The experiences and challenges of Canadian Forces Physiotherapy Officers deployed to Afghanistan

**Published:** 2010-07

**Authors:** Peter Rowe, Christine Carpenter, Luc J. Hébert

**Affiliations:** 1 CFHS GP HQ, D Med Pol, Physiotherapy, 1745 Alta Vista Drive, Ottawa (On); 2 Coventry University, United Kingdom; 3 Laval University, Quebec (Qc), Canada

**Purpose/Objectives and Rationale:** Military physiotherapists in the Canadian Forces meet the unique rehabilitation needs of military personnel. Recently, the physiotherapy officer role has evolved in response to the Canadian Forces' involvement in the combat-theatre of operations of Afghanistan, and this has created new and unique challenges and demands. The purpose of this study was to describe the experiences and challenges of military physiotherapists deployed to Afghanistan. Other literature defined and characterized the injury profiles of Canadian soldiers during the peacekeeping mission in Bosnia between 2000 and 2004 but did not explore the experiences and challenges faced by deployed physiotherapists. In addition, the focus of the Bosnia mission was significantly different than today's Afghanistan mission. **Relevance to the Physiotherapy Profession:** This type of systematic and comprehensive collection of information is needed to assist the Canadian Forces physiotherapy leadership in proactively preparing and supporting physiotherapists deployed on future missions. **Materials and Methods:** A qualitative research design was used within a framework of descriptive phenomenology that involved key informant recruitment and in-depth interviews as the data collection method. **Analysis:** Data analysis included verbatim transcriptions of interviews, thematic analysis, peer review and member checking. **Results:** Six military physiotherapists were interviewed. They described rewarding experiences that were stressful, yet highly career-satisfying. Main challenges revolved around heavy workloads, an expanded scope of practice as sole-charge practitioners, and the consequences and criticality of their clinical decisions. **Conclusions:** These findings suggest that enhanced pre-deployment training and the implementation of a stronger support network will improve the capabilities of our deployed military physiotherapists when deployed to difficult theatres of operations such as Afghanistan.

# Abstract: A068 – Considering the culture of customer service in the public health sector

**Published:** 2010-07

**Authors:** Rosemary R. Brander, Margo L. Paterson, Yolande E. Chan

**Affiliations:** Rehabilitation Therapy, Queen's University, Kingston, ON K7L 3N6 and Providence Care, Kingston, ON K7L 5A2; Rehabilitation Therapy, Queen's University; The Monieson Centre, Queen's School of Business, Ruffolo, Maurio, Providence Care, Kingston, ON

**Purpose/Objectives and Rationale:** To understand the culture of customer service in a public hospital. **Relevance to the Physiotherapy Profession:** The consideration of inter-professional meanings ascribed to customer service may assist in the development of new models of health care delivery. **Materials and Methods:** A mixed methodology including an ethnographic and quasi-experimental approach was used. Semi-structured interviews and/or focus groups were used to collect data prior to, immediately after and 5 months after the completion of 8 weekly education sessions related to Patient and Family Centered Care. A purposeful sample assigned volunteers to a control group of health professionals and an intervention group that included education with patients and/or family members. Method triangulation included field notes and an audit trail. Field observations and member-checking improved the credibility of the data. **Analysis:** A domain analysis was used to focus the thematic components related to culture. A componential analysis enabled a search for attributes within the cultural categories. Lastly a cultural inventory led to the determination of significant cultural themes. Significant themes were compared across control and intervention groups. **Results:** Customer service has different meanings and relevance to different inter-professional health providers. The value attributed to client relationships changed after the educational sessions. Little difference was apparent between control and intervention groups in the meaning they imparted to customer service. **Conclusions:** Educating health professionals together can impact change in meanings ascribed to the value of customer relationships. Including patients and families in inter-professional education needs further study.

# Abstract: A073 – Tele-Rehab: Feasibility of using telemedicine to deliver interprofessional stroke rehabilitation consultations in rural and remote communities

**Published:** 2010-07

**Authors:** KJE Reinikka, E French, M Huijbregts

**Affiliations:** Thunder Bay Regional Health Sciences Centre, Northwestern Ontario Regional Stroke Network; Northern Ontario School of Medicine, Thunder Bay, ON, P7B 5E1, Canada; Thunder Bay Regional Health Sciences Centre, Northwestern Ontario Regional Stroke Network, Thunder Bay, ON P7B 7C7, Canada; Baycrest, Quality, Risk & Patient Safety, Toronto, ON M6A 2E1, Canada

**Purpose/Objectives and Rationale:** Aboriginal people experience stroke at higher than national rates. Northern, rural, and remote populations have limited, if any, access to stroke rehabilitation. Innovative use of telemedicine can overcome geographic and human resource barriers restricting access to care. This study examined feasibility of using telemedicine for interprofessional stroke rehabilitation consultations (Tele-Rehab) in rural and remote communities. **Relevance to the Physiotherapy Profession:** Telemedicine is an innovative means by which physiotherapists can provide care to clients in underserviced and remote areas.

**Description:** Pre-post study using mixed methods. **Participants:** People with stroke (PwS) discharged from hospital, without access to rehabilitation in their community; consulting rehabilitation clinicians. Intervention: Interprofessional Tele-Rehab consultations (assessment and treatment by physiotherapists, occupational, and speech therapists, and social workers from an urban centre). Demographic, resource utilization, and perceived acceptability data was collected over a seven-month period. **Observations:** Seven PwS and four clinicians participated, with 10 consultations occurring. Clinicians successfully conducted client-centred consultations: an average of 7.22 recommendations and referrals were made per visit (range 4–10). Recommendations focused on mobility, education, resuming roles, and community participation. The Tele-Rehab experience was generally rated good-excellent: 95.23%, SD = 0.44, PwS reported poor-fair audio: 75%, SD = 0.82, however all participants would recommend Tele-Rehab to others. **Critical Assessment:** Healthcare, community, and telemedicine partnerships were critical in building community capacity and implementing Tele-Rehab. Results suggest that PwS experience continuing rehabilitation needs post-discharge. Tele-Rehab is a feasible means of providing ongoing stroke rehabilitation for this group of participants. **Conclusions:** Telemedicine and innovative models of care can extend the reach of rehabilitation professionals into communities where access to physiotherapy is limited.

# Abstract: A074 – Introducing rehabilitation services to a health care organization in rural Haiti

**Published:** 2010-07

**Authors:** SR Cleaver

**Affiliations:** Ho○pital Albert Schweitzer Haiti, Deschapelles, Haiti

**Purpose/Objectives and Rationale:** To improve health and reduce the burden of disability among people in the catchment area of Ho○pital Albert Schweitzer Haiti (HAS Haiti) through the establishment of rehabilitation services. **Relevance to the Physiotherapy Profession:** A large portion of the world's population has no access to rehabilitation. North American physiotherapists have the potential to improve the quantity and quality of rehabilitation care in various settings worldwide by stimulating local activity through partnership. **Description:** We, a group of North American physiotherapists with an interest in Haiti, committed to improving rehabilitation in the country and partnered with HAS Haiti. We conducted needs assessment that identified a large incidence of disabling conditions, a lower quality of life people with disabilities and few existing services. We thus participated in fundraising activities, increased awareness of rehabilitation, established a training program for rehabilitation technicians, created a system to integrate the technicians into HAS Haiti facilities and nurtured a local rehabilitation leadership structure. **Observations:** Rehabilitation services are now being offered by rehabilitation technician graduates supervised by a local physiotherapist. **Critical Assessment:** Ongoing efforts must address the following outstanding issues:

- The engagement of people with disabilities in program leadership and direction,
- The role of international involvement,
- The integration of society-level intervention such as disability prevention and the inclusion of the disabled,
- Increased consideration of local views, including those on traditional healing and priorities for intervention.

**Conclusions:** The establishment of rehabilitation services at Ho○pital Albert Schweitzer Haiti has led to increased local capacity to address disability and health issues.

# Abstract: A078 – Best Practice Guidelines for the interprofessional management of hypertonicity

**Published:** 2010-07

**Authors:** T Paulenko, D Hebert

**Affiliations:** Toronto Rehab, Toronto, ON M5G 2A2, Canada

**Purpose/Objectives and Rationale:** The purpose of this project is to develop and implement best practice guidelines for the interprofessional management of hypertonicity. **Relevance to the Physiotherapy Profession:** Hypertonicity manifests in the majority of persons living with an upper motor neuron lesion such as brain injury, multiple sclerosis and stroke. As this impairment interferes with functional movement, occupational performance and community re-integration, it is a key concern to neurorehabilitation professionals delivering patient-centred care. Management consists of a variety of approaches, often multidisciplinary in nature. Limited research guides intervention planning and rarely is an interprofessional approach used. **Description:** Across our programs, an interprofessional team of nurses, occupational therapists, pharmacists, physiatrists and physical therapists collaborated on guideline development. Methodologically, we used a systematic review of the literature, an environmental scan and consensus building. To date the group has completed the overall guideline, which includes recommendations for team assessment, identification of the type of hypertonicity, intervention pathways, outcome measurement, follow-up timeframes and documentation. A number of intervention modules have been developed including stretching/passive range of motion, splinting/casting, neurotherapeutics, electrotherapy and pharmacology (primarily botulinum toxin). **Observations:** Guideline development has been invaluable in fostering interprofessional collaboration. In light of limited research, interprofessional groups continue to strive for consensus on best practice for each therapeutic intervention. **Critical Assessment:** Identified needs include continuing interprofessional development about the guideline and interprofessional documentation revisions to encompass professional requirements and enhance our pilot clinic processes. **Conclusions:** Today's limited healthcare resources and the chronic, complex nature of hypertonicity require an interprofessional collaborative approach for which our guideline serves as a best practice foundation.

# Abstract: A083 – Leading by example—Inspiring future client-centered inter-professional teams

**Published:** 2010-07

**Authors:** GA Creaser, D MacKenzie, C Barkhouse-McKeen

**Affiliations:** School of Physiotherapy; School of Occupational Therapy; School of Nursing, Dalhousie University

**Purpose/Objective and Rationale:** To create a client-centered, inter-professional assignment within curricula of six schools in a Faculty of Health Professions. To examine students' perceptions of the assignment's value for building interdisciplinary relationships. **Relevance to the Physiotherapy Profession:** Creativity is required to design meaningful learning opportunities for inter-professional education in a large, diverse faculty. The challenges to inter-professional education range from large enrollments to varying entry-to-practice requirements to managing the different academic timetables. **Description:** Faculty from the Schools of Physiotherapy, Occupational Therapy, Nursing, Human Communication Disorders, Social Work and Pharmacy developed a novel assignment to promote collaboration among students in the context of a naturally-occurring team. Groups comprised of students from each school collaborated in a two-stage team meeting to develop a comprehensive client care-plan. At the first meeting students introduced themselves, met a simulated client through a video, and reviewed the client's health information. On completion of the second meeting, each group submitted their Interdisciplinary Care Plan. Evaluation was based on the assignment's objectives but each school assigned the value. **Observations:** Disparity in class sizes, content knowledge and clinical fieldwork were accommodated by the simulation's structure. Factors influencing the groups' composition and communication were viewed as challenges that real-life health teams routinely encounter. **Critical Assessment:** Groups worked independently and focused on the client as they discussed roles, shared profession-specific knowledge and negotiated detailed care-plans. Students provided useful feedback about the assignment's value, structure and enhancements for next year's curricula. **Conclusions:** The team-meeting, client-centered format enables students to appreciate the challenges of interdisciplinary relationships, while learning with, from, and about one another.

# Abstract: A090 – Performance and injury in relation to specific acrobatic circus disciplines

**Published:** 2010-07

**Authors:** Ian Shrier

**Purpose/Objectives and Rationale:** Human circus arts are gaining increasing popularity as a physical activity: over 500 companies and 200 schools world-wide. The only data that currently exist are a few case reports and one survey. Analysis of Cirque du Soleil's Injury data bank was performed in order to be able to describe injury patterns and injury rates amongst its artists between 2002–2006 **Relevance to the Physiotherapy Profession:** Injury rates of a specific group of acrobatic and artistic performers similar to that of university/professional athletic population. A description and discussion of several circus specific injury mechanisms are related to the general athletic population **Description:** Descriptive Epidemiology Study combined with case studies. **Observations:** Injury was defined as a work-related condition recorded in an electronic injury database. Analyses for treatments, missed performances and injury rates (per 1000 artist/performances) was included and related to descriptive acrobatic injury cases. **Critical Assessment:** 1376 artists sustained a total of the 18,336 show- or training-related injuries. Most injuries were minor: out of the 6701 injuries with exposure data, 80% required ≤7 treatments, and resulted in ≤1 completely missed performance. The overall show injury rate was 9.7 (published NCAA Women's gymnastics rate was 15.2 inj/1000 athlete-exposures). The rate for injuries resulting in more than 15 missed performances for acrobats (highest risk group) was 0.74, much lower than the corresponding estimated NCAA Women's gymnastics rate. **Conclusions:** Most injuries in circus performers are minor and rates of more serious injuries are lower than many NCAA sports. Injury mechanisms are specific to acrobatic requirements-many unusual factors need to be taken into consideration.

# Abstract: A098 – Digital ‘storytelling’ within a social network: A blended learning appraoch to clinical reasoning in paediatrics

**Published:** 2010-07

**Authors:** K Freeman, L Davidson, L Pelland

**Affiliations:** Queen's University, School of Rehabilitation Therapy, Kingston, ON K7L 3N6

**Purpose/Objectives and Rationale:** Providing sufficient clinical reasoning (CR) practice to students is challenging, particularly in paediatrics were fieldwork opportunities are limited. The aim of this study was to describe and evaluate the learning experience of students using digital storytelling of authentic cases within a social network (SN) to practice CR. **Relevance to the Physiotherapy Profession:** CR is a pillar of the scholarly practice of physiotherapy and one of the domains where new graduates often lack confidence. Therapists could find the mentoring in virtual communities of practice to support their independent practice, including in under-serviced sectors, such as paediatrics in rural regions. **Materials and Methods:** A modified ADDIE model was used to develop the educational module; expert review guided alignment and validation of content to CR evidence. The SN included students in paediatrics (*n *= 78) and an inter-professional (IP) group of clinicians (*n *= 8). Stories were posted on blogs, and feedback provided in discussion forums. Information on students' learning experience was obtained by focus group and questionnaire. **Analysis:** Content analysis and ethnographic summarization of responses, with independent review of identified meaning units and thematic tags by two researchers. **Results:** Educational benefits included: improved skill and confidence in finding information, articulating and justifying clinical decisions; recognition of the range of clients' experiences, ‘stories’ and lifelong meaning of paediatric conditions; and modeling of IP practice in CR. Negative experiences were related to the technology, and students' insecurity with creating their knowledge. **Conclusions:** SN can provide an authentic CR experience; strategies to decrease time commitment to learn technology and students' resistance to active learning approaches need to be addressed.

# Abstract: A100 – Draping education to promote patient dignity: canadian physiotherapy student and instructor perceptions

**Published:** 2010-07

**Authors:** Nicole Wilson, Diana Hopkins-Rosseel

**Affiliations:** School of Rehabilitation Therapy, Queen's University, Kingston, ON K7L 3N; School of Rehabilitation Therapy, Queen's University, Kingston, ON K7L 3N6

**Purpose/Objectives and Rationale:** Patient dignity is an important concept in healthcare. One element of dignity is physical exposure/draping. The purpose of this study was to compare current perceptions of draping education from physiotherapy students and instructors. **Relevance to the Physiotherapy Profession:** As physiotherapists provide care that may physically expose patients, they must be proficient in draping techniques to promote dignity. **Materials and Methods:** A mixed methods survey design. Surveys were distributed to instructors and first year students in Canadian physiotherapy programs in August, 2009. **Analysis:** Descriptive statistics and chi square analysis (*p *= < 0.05). **Results:** 127 students and 183 instructors responded. 99% of all respondents believed that it is important for students to learn draping techniques. More instructors than students agreed that draping has an impact on patient dignity (*p *= 0.007). Instructors felt students have more difficulty with draping than what students perceive (*p *= 0.012). Instructors felt comfortable teaching how to accommodate cultural modesty concerns, but students were not as comfortable with these accommodations (*p *= 0.011). Although 63.8% of instructors taught specific draping skills, 70% of students stated they had not learned draping for specific examinations and 61% of students believed additional draping educational time is warranted. Instructors identified draping as not included or poorly defined in the curriculum, other skills taking precedence, and lack of educational resources as barriers to teaching draping. **Conclusions:** Although most educators taught draping skills and how to accommodate patients with cultural modesty concerns, students did not feel they had learned appropriate draping techniques and that additional draping education should be provided.

# Abstract: P002 – Benefits and harms of international clinical placements in entry-level physiotherapist education

**Published:** 2010-07

**Authors:** M Fraser, SR Cleaver, G Wainwright

**Affiliations:** South City Physiotherapy, Guelph, ON, Canada; Ho○pital Albert Schweitzer Haiti, Deschapelles, Haiti; School of Physiotherapy, Dalhousie University, Halifax, NS, Canada

**Learning Objectives and Session Content:** Upon completion of this presentation, participants will:

1. Be aware of past, current and projected trends in international clinical placements (ICPs) associated with entry-level physiotherapist education.
2. Understand the potential benefits of ICPs for physiotherapy students, sending institutions, and host communities.
3. Understand the potential limitations and disadvantages of ICPs in physiotherapy.

**Relevance to the Physiotherapy Profession:** Global health educational initiatives have become more popular among students and faculty throughout physiotherapist education programs across Canada/North America. An increasing number of students in North America are engaging in international clinical placements (ICPs) as part of their academic training. Despite this growth, limited research has been done to explore the impacts of ICPs. These activities are potentially beneficial for the students, for the sending institutions and for the hosts. Often left unrecognized, however, are the potential negative effects as ICP activities place added demands upon students, faculty, and host communities. These activities often occur in resource-poor settings that are more sensitive to change, making the host communities even more vulnerable to potential harms. It is incumbent upon the physiotherapy profession to critically examine these partnerships for the benefit and protection of all stakeholders rather than spend resources organizing programs whose potential negative impacts have not been studied.

**Target Population:** This session will be of interest to a broad target audience that is interested in global health initiatives, including entry-level and graduate students, educators and clinical coordinators.

**Description of Supporting Evidence:** As students and universities are increasingly participating in ICPs, it is imperative that the benefits and challenges be examined. Recent evidence has shown the increasing trend towards global involvement among physiotherapy students in Canada and in the United States of America. Studies have demonstrated benefits of global activity for the students (increased cultural competency, improved interpersonal skills) and for the host institutions (improved reputation as the institution is seen to be concerned with global health issues). Some seminal research has identified potential harms for the students (related to security, related to potentially missed opportunity to learn the clinical skills required for practice in their home country) and for the sending institutions (resources allocated to projects that may not offer significant returns). However, there is a glaring absence of research in the literature that examines the perceived advantages and the potential harms for the host communities.

**Description of Session Format:** This 60 minute debate will address some of the complex issues involved in international clinical placements. The convener will provide a brief overview of the emerging trends in clinical placement activities between global north and south communities. Key concepts related to positive international involvement including cultural competency and reciprocity will be highlighted, and ethical considerations will be introduced. Panelists will constructively debate the potential positive and negative effects of ISL activities in entry-level physiotherapist education, considering the multiple stakeholders and focusing on the key concepts presented.

**Conclusions and Implications:** International clinical placements are becoming more common in entry-level physiotherapist education. These activities have potential positive and negative effects on multiple stakeholders. This educational session will highlight issues surrounding ICP activities and stimulate dialogue on the subject in the physiotherapy profession through its debate format. This educational session will provide students, clinicians and faculty with information that will provide insight to be drawn from when designing ICP programs.

# Abstract: P003 – Canada's physiotherapists and the New Veterans Affairs Canada Rehabilitation Program

**Published:** 2010-07

**Authors:** B MacCormack, C Basque Godin, VR O'Neill

**Affiliations:** National Rehabilitation Program, Veterans Affairs Canada

**Learning Objectives and Session Content:**

1. Participants will gain a practical understanding of the new Veterans Affairs Canada (VAC) Rehabilitation Program, focussing on physiotherapy practice.
2. Practical Application: Through case studies, participants will see the VAC Rehabilitation Program in action. This will allow participants to see how the program seeks to open the door for the provision of timely, needs-based, innovative rehabilitation services.
3. Research Results: Participants will become familiar with exciting new research developed by VAC, DND and the Canadian Institutes of Health Research.
4. Invitation to Discuss: Participants will have an opportunity to ask questions and to contribute to discussion on the role for physiotherapy and multi-disciplinary team approaches to the rehabilitation problems faced by Veterans and their families.

**Session Content:** The challenges around the rehabilitation of returning First and Second World Veterans played a pivotal role in the development of physiotherapy in Canada. With Canada's involvement in Afghanistan and numerous other overseas military operations, a new generation of Veterans now faces the challenge of rehabilitation and reintegration into civilian life. VAC's new Rehabilitation Program, now three years in operation, focuses on rehabilitation and reintegration into civilian life through the provision of needs-based services designed to promote “wellness”. At a glance the VAC Rehabilitation Program provides:

- Medical Rehabilitation to stabilize physical and psychiatric conditions and restore basic function;
- Psycho-social Services: To restore, to the extent possible, the capability and functioning of CF Veterans and their families; and to restore independence and support adjustment to disability; and
- Vocational Rehabilitation to identify and achieve vocational goals.

**Relevance to the Physiotherapy Profession:** Physiotherapists play an important role in the provision of health services to Veterans. They are an integral part of Veterans Affairs Canada's nation-wide network of registered service providers. Canadian Veterans are seen in substantial numbers by Physiotherapists daily. Physiotherapists have worked with Canada's aging traditional Veteran population for many years. Services were provided to clients under clearly defined Benefits for physio assessment, treatment and acupuncture. These services continue for eligible clients. Under the VAC Rehabilitation Program physiotherapists have an important role in identifying physical barriers to re-establishment and in designing and implementing individualized rehabilitation programs for Veterans or eligible family members. Implications for Practice: A review of relevant case studies will demonstrate the VAC Rehabilitation Program in action. We will examine the implications of such program features as a focus on restoring function at home and in community and through vocational rehabilitation, and a holistic, coordinated approach to client's rehabilitation needs.

**Target Population:** This session will be of interest to rehabilitation, orthopaedic and out-patient physiotherapists who may provide physiotherapy services to Canada's Veterans, and to all those interested in issues related to Veterans' health. Typical military service-related injuries seen by physiotherapists include musculoskeletal injuries, disc disease and spinal conditions, psychiatric conditions, fractures, nervous system disorders, accident /external violence, and gunshot wounds, as well as chronic pain disorders, mild to severe traumatic brain injury, amputations, and the problems of general de-conditioning following re-entry into civilian life.

**Description of Supporting Evidence:** Preliminary results from exciting new research by VAC, DND and the Canadian Institutes of Health Research will be shared. For example, initial findings on ‘How modern-day Veterans are doing after they transition to civilian life in terms of income, health, well-being and disability’. Information Sharing: Information on the VAC Rehabilitation Program will be made available to participants.

**Description of Session Format:** This session will be a lecture format, (30 minutes) with opportunity for questions and for participant discussion regarding possible enhanced physiotherapy services to Veterans and their families. (30 minutes.)

**Conclusions and Implications:** Meeting the Challenge: Ensuring that innovative physiotherapy programs promoting independence and wellness are available to clients in a timely fashion and in the client's home community is our challenge. This has often been termed: “The right care, at the right time, in the right place”. Important Implications for the Physiotherapy Community: It is our hope that this program will encourage rehabilitation programming that supports early intervention, evolves with the client's changing stage of transition and is tailored to individual client needs. We welcome your interest in these programs and encourage physiotherapists to work with local VAC case managers and health professionals to ensure that client's physical rehabilitation needs are being appropriately and adequately met. Finally we would like to encourage you to consider identifying opportunities for future research in the important emerging area of Veterans' Health.

# Abstract: P008 – Collegial conversations—A framework for those ‘hard to talk through’ moments

**Published:** 2010-07

**Authors:** B Mori, C Evans, BE Gibson

**Affiliations:** Department of Physical Therapy, University of Toronto, ON M5G 1V7, Canada

**Learning Objectives and Session Content:** The focus of this interactive session will be to define what constitutes professional behaviours and equip participants with a framework to manage a difficult conversation. This framework is based on the Collegial Conversation framework (Branigan & Ginsburg, 2007) as a method to teach and learn professionalism with medical students. Upon completion of this session, attendees will be able to:

1. Define professionalism and recognize professional and unprofessional behaviour.
2. Identify common responses to difficult conversations.
3. Discuss and employ a process to manage collegial conversations.

**Relevance to the Physiotherapy Profession:** As health professionals, we are guided by principles of behaviour, codes of conduct, laws and our own personal integrity. Our Provincial Regulators and the Canadian Physiotherapy Association provide documents which specify the practitioner's commitment to the patient, profession, employers and society. As registrants, we are bound to this code of ethics and occasionally find ourselves in a position where we should have a conversation with another individual regarding their professionalism or ethical practice. This is often a difficult conversation and frequently does not occur even when we know there would be significant benefits to confronting an issue. By providing this workshop, we hope to enable attendees with the knowledge, skill, attitude and practice to have collegial conversations and uphold our professional commitment which helps instill confidence in the discipline, and serves as a concrete reminder of the high standards of professional conduct that have come to be synonymous with the practice of physiotherapy.

**Target Population:** This workshop would be applicable to a broad range of attendees: those working in the public and private sector, individuals in leadership positions or individuals who work and interact with others taking active roles in interprofessional teams and education. Our small group breakout sessions will provide case scenarios for collegial conversations with: colleagues, patients/clients, and learners/mentees.

**Description of Supporting Evidence:** Professional growth and development are associated with the conditions, forces and interactions that take place in the individual's environment (Dewey, 1934). The interactions that take place between colleagues may be in the form of observations, or conversations and discussions. Professionals readily use their peers to seek advice on cases and patient issues (Rappolt, 2002); but colleagues are often reluctant to raise matters such as professional behaviors that are more delicate and potentially uncomfortable to address. When we review professional behaviours with students, we find unprofessional behaviours in medical students are associated with subsequent disciplinary action as registered professionals (Papadakis et al, 2004; Stern et al, 2005). Hayes et al found that while most unacceptable behaviours in physical therapy students were related to poor communication and unprofessional behaviour, these behaviours were not addressed. The reasons behind the reluctance to discuss professional behaviors with a colleague or learner are complex. The literature suggests that the barriers in addressing problematic behaviours may result from personal discomfort, belief that the behaviour or attitude is not changeable, lack of experience or skill in having difficult conversations, or feeling sorry for the recipient (Burack et al, 1999). Even when these behaviors are addressed, it is often with non-verbal hints such as a glance or with humor. While this indirect non-confrontational feedback helps preserve the individual's self esteem, it may require too much inference on the part of the learner/peer and therefore may not reliably provide them with the information necessary for accurate self assessment or their ability to modify their behaviour (Burack et al, 1999). Learning how to become mindful of relationship dynamics and a desire for a more collaborative environment, may evoke behavior change and enhanced collegial discussion (Suchman et al, 2004).

**Description of Session Format:** This learning session will integrate didactic and interactive activities and include small group and case based learning approaches. It will conceptually combine elements of research, education and practice. Participants will draw upon their own practice to identify applications in their settings. The format of the workshop will be as follows: Introduction (5 minutes); Background on difficult conversations and professionalism—Didactic presentation with slides (15 minutes); Group Exercise with three cases and debrief—Large group exercise (30 minutes); Presentation of Collegial Conversation framework—Didactic presentation slides (20 minutes); Case demonstration and discussion (15 minutes); Small group breakout session with 3 cases (20 minutes); Debrief small group role play—Large group discussion (10 minutes); Independent reflections & Evaluations (5 minutes).

**Conclusions and Implications:** By providing this learning opportunity, we hope to enhance the knowledge, skills, attitude and practice of participants to promote collegial conversations within and beyond the physiotherapy profession.

# Abstract: P017 – Interdisciplinarity: Strength through diversity

**Published:** 2010-07

**Authors:** M Thornton, T Dutton

**Affiliations:** Dalhousie University, Halifax, Nova Scotia; Nova Scotia Community College

**Learning Objectives and Session Content:**

1. To better understand the many dimensions and benefits of interdisciplinarity.
2. To apply the principles and new understanding to the participants' specific situations in work and professional life.
3. To work together to propose a plan for physiotherapy to continue to excel in the interdisciplinary environment, positioning ourselves as a profession that is flexible and strong.

**Relevance to the Physiotherapy Profession:** Physiotherapists work within a changing environment and not only have they survived… but they have been willing to evolve and adapt practice. In fact they have thrived, leading change in many different environments. It has been through interdisciplinarity, working with others effectively and showing strength of knowledge and character that the profession is well recognized and regarded in health care.

**Target Population:** This workshop will be valuable to physiotherapists working in all areas of the profession. It will interest those who work in settings where interdisciplinary teams are common. It will also be relevant to those who work in clinics that employ mostly physiotherapists and to those in academia.

**Description of Supporting Evidence:** In this workshop we will talk about interdisciplinarity as ‘strength through diversity’. It is also referred to in the literature as collaborative research, team teaching, borrowing across disciplines, a more holistic approach, and permeability of boundaries (Thompson Klein, 1990). Interdisciplinary teamwork allows the answering of more broad and complex issues than ever addressed before, and solving problems beyond the scope of one discipline. It has been described as a state where people feel nostalgia for the loss of wholeness while at the same time being a new stage in evolution of science. The term nondisciplinary has been used indicating expanding boundaries (Lattuca, 2001). Fewster-Thuente (2008) describes communication as being very important to interdisciplinary collaboration. They cite a lack of good communication and collaboration as possibly being responsible for as much as 70% of the adverse events currently reported in healthcare. Factors such as gender, age, culture, and level of education may impact the perceived collaboration. A recent phenomenon is transfer to electronic health care records and McGurkin et al (2006) discuss the value of interdisciplinary collaboration in developing electronic records. While students on healthcare teams found the teamwork benefited the patient and was a productive use of time, senior residents were inclined to be less positive (Leipzig, 2002). A weakness in the system was realising that there has to be someone who has the authority for decision making. Yet the opportunities for leadership are there and Anonson and colleagues 2005 describe a campaign to ‘expect a leader’. While the literature is not prevalent with physiotherapy specific examples, but one recent example is that the College of Physiotherapy of Ontario recently was awarded silver at the at the e-leadership healthcare awards held at the Healthcare internet conference 2009, Los Vegas.

**Description of Session Format:** This session will be in the format of a panel discussion with opportunity for small group discussion as well as questions and answers of the panellists.

**Conclusions and Implications:** This session will explore the theme of interdisciplinarity from the point of view of education and different work environments. The discussion should incorporate strengths of this direction of healthcare that allows for creative solutions and strength through diversity. A future direction is to understand the implications of technology, working with other disciplines and the ongoing benefits and risks of interdisciplinarity.

# Abstract: P020 – Patient safety: Putting safety into practice

**Published:** 2010-07

**Authors:** J King

**Affiliations:** University of Ottawa, Ottawa, ON, Anderson, CM., London Health Sciences Centre, London ON

**Learning Objectives and Session Content:**

1. Participants will learn about patient safety concepts including root cause analysis.
2. Participants will be able to use root cause analysis in a variety of clinical settings from an individual, department and facility perspective.
3. Participants will be able to identify specific strategies for putting patient safety factors into their practice.

This interactive session will introduce participants to key concepts in patient safety and how they relate to physiotherapy including a culture of patient safety, close calls and adverse events.

Using the Canadian Root Cause Framework from the Canadian Patient Safety Institute clinical cases will be analyzed that are commonly found in different practice settings including hospital, clinic and community. Participants will be asked to consider the analysis from the individual physiotherapist, physiotherapy department/program and the clinical /facility's perspective. The session will include a discussion of how physiotherapists can build patient safety into our day-to-day practice. This will include topics such as changing the culture, getting away from the blame game, and simple tips to incorporate safety as a role for all practitioners.

**Relevance to the Physiotherapy Profession:** As recognized in the CPA's position statement for patient safety, regardless of role or setting, patient safety is a concern for all physiotherapists. Physiotherapists and physiotherapy support workers, working in clinical settings, need to be able to provide safe effective patient care. Physiotherapists working in administrative or policy roles in health care facilities need to be knowledgeable about patient safety in planning and evaluating programs. Physiotherapists working in educational roles need to include some basic elements of root cause analysis in their curricula. Physiotherapists working with regulatory organizations need to be aware of the current safety issues and concerns, have an understanding of root cause analysis and have knowledge of safety strategies for practitioners in order to better protect the public.

**Target Population:** Physiotherapists working in clinical, administrative, educational roles and regulatory organizations.

**Description of Supporting Evidence:** Over the last few years, patient safety has become a key health care concern throughout the world. This has resulted in the patient safety literature growing exponentially, with strong evidence to support interventions including root cause analysis. A list of key patient safety references will be provided to the participants.

**Description of Session Format:** This 1-hour interactive session will include an introduction to patient safety including root cause analysis, and the session will conclude by an interactive group analysis of clinical situations and identification of patient safety strategies for day-to-day practice.

**Conclusions and Implications:** At the end of this interactive session the participants will have a greater understanding of patient safety, as well as having gained knowledge of root cause analysis how to incorporate this knowledge into practice. It is hoped that their understanding of patient safety concepts will help them grow as professionals by providing them with knowledge and skills that could be incorporated in future health care interactions for the benefit of patients.

# Abstract: P027 – Struggling with using outcome measurement? Current strategies underway in BC to win the outcome measurement battle

**Published:** 2010-07

**Authors:** PJ Lawrence, M Duggan, A Hoens

**Affiliations:** Vancouver Acute, Physiotherapy, Vancouver, V5Z 1M9, Canada; University of British Columbia, Department of Physiotherapy, Vancouver, V6T 1Z3, Canada

**Learning Objectives and Session Content:** This session will build on the Congress 2009 Outcome Measurement (OM) session presented by Deb Kennedy, Dr. Paul Stratford and Greg Alcock by sharing strategies underway in BC to address the adoption of OM in Total Joint Arthroplasty (TJA). The objectives of the session include:

1. To describe the current state of OM for TJA in BC in terms of:
  - Which outcome measurements are being used
  - How they are being used
  - Why they are not being used optimally.
2. To describe the desired state of OM for TJA in BC
3. To provide strategies to foster movement to the desired state.

*Description of Current State:* Although there is a general acceptance of the importance of incorporating OM in practice, evidence suggests that its current utilization is variable. The common assumption (prior to this project) was that administrators typically utilize this type of information for program evaluation only and rarely do clinicians use it to guide individual patient treatment planning.

In recognition of the need to determine the actual utilization patterns for OM, and to assist clinicians in the adoption of OM in the TJA population, four unique and complementary initiatives, were undertaken in BC. First, in order to establish a baseline for current utilization, a team comprised of a practice leader and clinicians across practice settings undertook a formalized program evaluation to determine how OM is currently being utilized in TJA. Second, also to inform the baseline in terms of determining which outcome measures are currently being and subsequently should be used, the Provincial Rehabilitation Advisory Group struck a subcommittee responsible for the development of an inventory of outcome measurement tools that met established criteria. The inventory was augmented with a questionnaire to survey current practice utilizing these measures. Third, a MRSc project, utilizing a qualitative study design, was undertaken to determine why clinicians do or do not use OM (barriers and facilitators) in TJA. And fourth, an educational package, produced by a PT Knowledge Broker and PTs who pursued PhDs in this area, was developed to educate practitioners in easy and effective use of OM.

*Desired state:* The collective information for the above synergistic initiatives was used to design an implementation plan to assist clinicians and decision makers in using the preferable outcome measures, in the optimal manner, to inform both clinical interventions and program decisions.

*How to make it work:* The presenters for this session (a Practice Leader, a Clinician and Physical Therapy Knowledge Broker) have designed this education session to share findings and creative strategies to enhance use of OM.

**Relevance to the Physiotherapy Profession:** Total joint replacement is an increasingly common surgery throughout Canada. There are approximately 62,000 TJA surgeries each year, with 10,700 performed in BC in 2008 (CIHI, 2008). This number is expected to grow as the population ages. Most of these clients receive physiotherapy services at some point during their journey through the health care system. Despite the appreciation that appropriate use of outcome measures can enhance care, improve quality of life, may shorten recovery time (thereby reducing health care costs) and help to predict which clients will require more services, the utilization of OM to guide practice across the continuum is currently suboptimal. This session will build on discussions started at Congress 2009 by profiling strategies to increase OM utilization in the TJA population. Further, it will provide a template for participants interested in increasing use of OM in their respective patient populations.

**Target Population:** Clinicians, Practice Leaders and Operational Managers.

**Description of Supporting Evidence:** The number of THA and TKA surgeries in Canada is on the rise. This is in part due to the aging of the population, increasing prevalence of obesity and growing number of adults with hip and knee osteoarthritis (Badley 1995, Birrel 1999). Coupled with technical and procedural advances in arthroplasty surgery and patient expectations, one can expect the demand for THA and TKA to continue to grow. While large international joint replacement registries have been successful in collecting short-term peri-operative data and long-term surgical outcomes, few record patient outcomes in a prospective fashion to guide individualized patient care. Turner-Stokes et al, (1997) and Stokes et al, (2008) reported that recognition of the importance and use of outcome measures has increased in the last decade. However, measurements are often taken primarily at admission and discharge, sometimes at admission only and even if the scores are analyzed and collated they are not (consistently) used to guide day-to-day practice. Huijbregt et al (2002) further stated that PTs are confused about the selection, administration, and interpretation of outcome measurement tools. To date, there is no agreement on what patient outcomes and measures provide the most meaningful information to guide clinicians in treatment planning, decision-making and program evaluation. Consequently, OM is in limited use in clinical settings (Westby 2009) and inconsistent/highly variable in the published research (Riddle 2008). As a result, it is difficult for clinicians to integrate the research evidence into clinical practice and compare rehabilitation interventions across settings, patient demographics and throughout the continuum of care. For outcome measurement to guide clinical decision making and be meaningful to both patients and rehabilitation providers, tools need to meet both standard psychometric criteria and clinical utility properties. As well, clinicians need to be informed on how best to administer and utilize the information from OM to inform practice. (Alcock, Stratford, Kennedy 2009). Effective strategies to enhance adoption of OM must include attention to sustaining change. These strategies need to address the following components: PTs are aware of appropriate OM utilization, agree with the proposed changes in practice, are willing to try out the proposed changes and to integrate changes into day to day practice (Pathman et al. 1996). A failure to address these components typically enables competing demands to nullify initial changes in practice. Creative strategies are required to embed OM use into routine (Virani et al, 2009).

**Description of Session Format:**

1. Presentations:
  - A summary of the resources, processes and outcomes (description of current state) of each of the sub projects.
  - A description of the desired state for OM.
2. Expert Panel (including two of speakers from 2009 presentation) discussion regarding strategies for successful implementation of an OM strategy including reference to the change management literature.
3. Break out groups re: implementation of strategies in participants' area of practice (participants will work in small groups with colleagues from similar practice areas). A panel member will facilitate each group. The objective will be to incorporate and build on the principles provided in the presentations and the panel discussion.

**Conclusions and Implications:** This session will provide participants with a foundation of theoretical and practical ‘how-to’ strategies to enhance the utilization of outcome measurement in their own practice settings. The inclusion of an array of perspectives provided by a panel incorporating a researcher, a knowledge broker, a clinician and practice leaders ensures that the potential spectrum of participants needs will be competently addressed.

# Abstract: P030 – The annual performance appraisal: Addressing more than just performance

**Published:** 2010-07

**Authors:** E Lynne Geddes, Caroline Gill

**Affiliations:** Senior Manager Therapy Health Care Inc. Burlington ON, and, Assistant Dean MSc(PT) Program and Clinical Professor School of Rehabilitation Science, McMaster University, Hamilton ON; Senior Manager Therapy Health Care Inc. Burlington ON

**Learning Objectives and Session Content:** During this presentation, participants will:

- discover the clinical relevance of the annual performance appraisal as a professional and health human resources management tool
- explore with other participants their opinions and experiences with giving and receiving a performance appraisal
- learn about the annual professional review process used by our company, including a description of the components, a presentation of the documents used, and a discussion of the strengths and barriers.

**Session Content:** This interactive session will discuss the role of the annual performance appraisal in meeting internal quality assurance standards and patient safety expectations as well as external factors such as third party contractual and regulatory body obligations. Participants will be asked to share their attitudes and experiences in giving and receiving a performance appraisal, including the benefits, barriers and challenges they have encountered. Building on the practical experiences of all participants, implementation of performance appraisals in different practice contexts, such as hospital, clinic and home care, will be compared and contrasted. In particular, this session will consider the similarities and differences in an inter-professional home care setting where therapists function as fee for service independent practitioners. After discussing the principles and features of a performance appraisal, the presenters will present the professional review process used at their company and will provide practical examples of the various components. Their company is an inter-professional private community rehabilitation company whose therapists are independent contractors. The professional review process, initiated in 2000, is competency-based and occurs in three parts. Part 1 includes chart audits and joint visits. This is usually completed by the professional practice leader with the therapist. Central to this part of the review is clinical observation and mutual dialogue related to the provision of direct client care. Part 2 involves professional self-reflection by the therapist. The therapist summarizes his/her lifelong learning events of the past year, describes how his/her professional goals were achieved over the year and sets goals for the coming year. This portion of the professional review is harmonized with expectations of the regulatory body. Part 3 includes an interview and development of an action plan, completed collaboratively between the therapist and the manager. Central to this part of the review is 2-way feedback (therapist ↔ company), client feedback, comparison of individual to team performance standards and discussion of how the therapist's practice aligns with the company's mission, vision and values. Between July 2007 and June 2009, 33 annual professional reviews were completed within our company involving 26 therapists from four professional groups (occupational therapists, physiotherapists, social workers, speech language pathologists). Quantitative and qualitative results from 13 randomly chosen professional reviews will be shared with participants. Therapists most frequently commented (7/13) on the value of and their appreciation for this professional review process. Completion of annual professional reviews provides documentation and current knowledge of a therapist's practice and enhances team building. They also help to inform the company's human resources practices and decisions. However, there are also challenges related to scheduling the reviews, the time commitment required by the therapists, the cost to the company, the stigma or apprehension experienced by both the therapist and the assessor, and the level of confidence held by the professional practice leader in their own skills and practice to be an assessor. The session will conclude with describing three new initiatives under development for the professional review—personalized job description review, peer-led joint visits, and mechanisms to include feedback from colleagues into the therapist's review—and will summarize the key points of the presentation.

**Relevance to the Physiotherapy Profession:** Health care providers, including physiotherapists, are expected to provide high quality, safe, and cost-effective care, and to demonstrate this by evaluating service delivery and clinical expertise at the individual and team level. The performance appraisal is one tool for evaluating the performance and clinical competence of the health care professional. It also helps inform team function and corporate professional development needs. As such, the performance appraisal is an important component of health human resources management and leadership development initiatives. Performance appraisals are clinically relevant to the physiotherapists whether involved at the point of care or within the management structure and whether organization is structured by department or program management.

**Target Population:** This topic will be of interest to physiotherapist at the point of care and those in managerial roles. The session will reflect the inter-professional nature of our professional review process with an emphasis on physiotherapy application. As such, it will be of interest to managers of physiotherapists and of other health care professionals. Given the dearth of research on performance appraisals in physiotherapy, it is also hoped that this presentation will provide impetus for research in this area.

**Description of Supporting Evidence:** Various methods for assessing performance are described in the literature including portfolios, critical incidents, reflection and performance appraisals. In a recent critical review of the evidence, the performance appraisal was the most frequently described and widely used assessment method. Recognized as vital to the progress of an organization, the performance appraisal process systematically assesses and monitors performance and provides feedback to the individual and to the company by comparing actual practice to established standards. The literature reports variability in what is included in a performance appraisal but it usually incorporates more than one format, such as self-assessment, measurement of indicators or questionnaires. No one best system has been identified. Nosse et al recommend the appraisal be based on a common transparent set of expectations, an accurate and equitable way to measure performance, and the information needed to measure and improve performance. While beneficial, many challenges have been identified in implementing performance appraisals, including time requirements, costs, and perceptions of employees and managers. The professional review used by our company seeks to achieve best practices by fulfilling Nosse et al's tripartite recommendations, incorporating multiple formats and sources of information, comparing actual practice to established standards, and providing feedback to the individual and to the organization.

**Description of Session Format:** This session will actively engage participants using a variety of formats including:

- large group discussion and presentation
- a ‘mini-quiz’ for participants
- small group activity and experiential sharing by participants
- ‘show and tell’ of each component of the professional review used by our company
- question and answer time.

**Conclusions and Implications:** Annual therapist performance appraisals are an essential part of health human resources management. While there are barriers and challenges to implementing them, there are significant benefits. At the individual level, performance appraisals encourage self-reflection on and provide affirmation of the therapist's practice. At the corporate level, performance appraisals inform health human resources decision making, assist in leadership development, support team building and assist in meeting accreditation standards. The process and new initiatives underway for our annual professional reviews meet the standards of Accreditation Canada and Commission on Accreditation of Rehabilitation Facilities.

# Abstract: P031 – The emerging role of physiotherapist support personnel in home care settings: Implications for physiotherapy practice and education

**Published:** 2010-07

**Authors:** Dianna Fong-Lee, Jane Lindsay, Stacey McPhail, Karen McIntosh, Suri L. Marken, Tanya Dutton

**Affiliations:** School of Health Sciences and Community Services, Conestoga College Institute of Technology and Advanced Learning, 299 Doon Valley Drive, Kitchener, Ontario, N2G 4M4, Canada; School of Health Sciences, Humber Institute of Technology and Advanced Learning, Toronto, ON, M9W 5L7, Canada; School of Health and Public Safety, SAIT Polytechnic, 1301–16 Ave NW, Calgary, Alberta, T2M 0L4, Canada; Health and Social Development Dept., 1000 KLO Road, Kelowna, British Columbia, V1Y 4X8, Canada; Nova Scotia Community College, Halifax, Nova Scotia

**Learning Objectives and Session Content:** The role and value of physiotherapist support personnel (PSP) in community based or home care settings, is often unrecognized given their unregulated status. This presentation will highlight the significant role of support personnel in achieving health, facilitating independence and community integration for individuals in their own homes; and their contribution towards the sustainability of physiotherapy service delivery in Canada now and in the future. At the end of this session, participants will be able to:

1. Identify the role of PSP in home care settings relative to other unregulated health care providers such as personal support workers.
2. Determine adequate levels of supervision, type of supports or standards that physiotherapists should provide to PSP for the safe delivery of physiotherapy services in home care settings.
3. Discuss the educational needs of PSP and entry-level physiotherapists to foster inter-professional and collaborative partnerships in home care settings.

**Relevance to the Physiotherapy Profession:** The changing nature of funding, growth in the elderly population, shorter hospital stays and early discharge rates have contributed to the need for increased community based services as alternatives to institutional or hospital-based care. To keep up with this demand, the physiotherapy profession has adapted and frequently finds it necessary to delegate or assign mobility and exercise training to unregulated health professionals such as PSP as well as personal support workers who are already in the home performing personal care and/or homemaking duties. This practice needs to be re-examined, given the proliferation of formally trained PSP who are now combined trained as occupational therapist assistants/physiotherapist assistants in addition to the expanded scope of practice for physiotherapists. More importantly, the growing role or expansion of the current scope of practice for personal support workers who are largely supervised by the nursing profession, suggests that the physiotherapy profession needs to advocate for role clarity and redefine current supervision standards and practice protocols to enhance the role of PSP in home care settings. Continued changes and developments in the delivery of physiotherapy services in home care settings reinforce the need to review the educational preparation of PSP, as well as entry-level physiotherapists; to enhance best practices for safe, collaborative and effective delivery of physiotherapy in home care settings.

**Target Population:** This session will be of interest to clinicians from a wide field of physiotherapy practice environments, physiotherapy support personnel as well as managers who are involved in the allocation and delivery of physiotherapy services.

**Description of Supporting Evidence:** The current use of PSP in home care settings varies greatly across Canada depending on legislation and funding support with some provinces only recently receiving funding and legislative support for the use of PSP in home care settings (Ontario Long-Term Care Act, 2009). On review of Health Canada's website, personal support workers are listed as part of the homecare labour force but PSP do not appear on this list. Lack of funding or legislative support contributes to the unrecognized role of PSP; however there continues to be considerable variation in the provincial utilization of PSP by physiotherapists across the country (Canadian Alliance of Physiotherapy Regulators, 2004). In a report prepared by the National Physiotherapy Advisory Group for the Canadian Physiotherapy Association, it was recommended that as more physiotherapists become aware of the scope of practice of PSP relative to other unregulated health care providers, “the profession will need to be proactive in developing the scope, supervision and practice domains of PSP…..and physiotherapists will need to review the implications and responsibilities of acting as supervisors of PSP and develop effective communication systems to ensure that tasks/responsibilities are clearly delegated and effectively understood” (Ashe & Mathur, 2004, p. 9). These recommendations are especially relevant with the shift from hospital based care to home care settings and the concomitant changes to the curricula of PSP as well as entry-level physiotherapists.

**Description of Session Format:** This session will be a podium presentation, with opportunity for participant discussion regarding experiences within their organizations.

**Conclusions and Implications:** The physiotherapy profession has a responsibility to appropriately utilize and supervise PSP and given the shift towards non-institutional care, the profession needs to carefully define, influence and promote collaborative partnerships with PSP and other unregulated health care providers. This presentation will encourage participants to critically review and share existing or newly developed protocols and standards for supervision and education of PSP in home care settings across the country.

# Abstract: P035 – Why is the security of your practice so critical? Practical knowledge and approaches to avoiding security breaches

**Published:** 2010-07

**Authors:** DH Hopkins-Rosseel, P Kasdan

**Affiliations:** School of Rehabilitation Therapy, 31 George Street, Queen's University, Kingston, Ontario, K7L 3N6; IBL Clinic Server, 238 Metcalf Street, Saint John, New Brunswick, E2K 1K6

**Learning Objectives and Session Content:** Participants will:

1. Understand the key laws and regulations that any private practitioner must comply with.
2. Be able to recognize and prevent the top 10 security risks for any private practice.
3. Learn how to set up systems to ensure they, their colleagues and staff are in compliance with the legislation.
4. Design a security audit of their own business' practices.
5. Understand the role of technology and information technology (IT) consultants in security.

**Relevance to the Physiotherapy Profession:** The physiotherapy profession is continually evolving with many new practice trends putting physiotherapists, their clients and their businesses at risk of security breaches. Practices handle very confidential client personal health information and client and business financial information. It is both an ethical and a legal imperative that this information be protected.

**Target Population:** This session will be of primary interest to any clinician working in the private sector in Canada. As autonomous practitioners, clinic owners and employers should be working together to know, develop, implement, and track the security practices where they work.

**Description of Supporting Evidence:** Many recent trends in physiotherapy practice have put our client's personal health information and our business' financial information at risk. As noted in the Canadian Alliance of Physiotherapy Regulator's ‘Scan of the Health Care Environment’ (CAPTR, 2000), there is a shift from hospitals to community practice. This, in turn, has led to an increasing business sector. Along with this move comes the need for the necessary skills to operate a business, including any legal and ethical responsibilities (Ashe & Mathur, 2004). Inter-professional practice patterns are also emerging and with them the development of expectations of the accrediting colleges, including in the area of essential competencies. (Verma et al., 2006). Not only must practitioners maintain their clinical practice skills but they must ensure their communications and record keeping meet all minimum requirements (CAPTR Analysis of Practice, 2008). Most importantly, technological advances in our environment have provided both practitioners and consumers with rapidly advancing methods of communicating and doing business. Along with these advances come the multiple challenges of balancing the benefits and the risks of actually using these technologies in practice. In the end, the practitioner must put in place systems within their practice to assist them in protecting client and business information and avoiding security breaches.

**Description of Session Format:** The session will start with a short, anonymous information gathering period, using clicker technology, to determine the security knowledge and practices of the participants. Then the presenters will review current key pieces of legislation and compliance requirements. This didactic component will be followed by a countdown of ‘The Top 10 Security Risks’ of any private practice. The session will finish with an interactive component where the participants will work together with the presenters to develop a checklist for a self-audit of their security systems and policies and procedures. The goal of the session will be to provide the participant with concrete information and skills to take back and successfully implement in their practices.

**Conclusions and Implications:** It is not only unethical to leave our practices open to breaches in security but it is in the profession's best interests to be both aware of, and skilled at, avoiding these breaches. Our clients, our referral sources, the payors of our services and our regulatory bodies need to be comfortable in the knowledge that we can be trusted to keep their information protected and secure. Once systems have been implemented in a practice, keeping track of developing information in the field and of their own security practices will become much less complicated and demanding.

# Abstract: P036 – Working collaboratively with physiotherapist support personnel

**Published:** 2010-07

**Authors:** D. Fong-Lee, Jane Lindsay, Siri Marken, Karen McIntosh, Stacey McPhail

**Affiliations:** Conestoga College, Occupational Therapist Assistant and Physiotherapist Assistant Program, Kitchener, ON, Canada; Conestoga College, Occupational Therapist Assistant and Physiotherapist Assistant Program, Kitchener, ON, Canada; Okanagan College, Kelowna, BC, Canada; Southern Alberta Institute of Technology, Calgary, AB, Canada; Humber College, Toronto, ON, Canada

**Learning Objectives and Session Content:** This 90 minute educational session will support the Leadership in Action stream, focusing on inter-professional collaboration and the evolution of the provision of physiotherapy services. By the end of the session, participants will be able to:

1. identify different levels of expertise, skill, and training of physiotherapist support personnel,
2. identify the range of interventions that can be assigned to physiotherapist support personnel related to the health care setting and
3. discuss supervision strategies that enhance collaborative, client-centered care in a fiscally responsible health care setting.

**Relevance to the Physiotherapy Profession:** Providing physical therapy services to a variable and aging population during a period of fiscal restraint and health care cutbacks provides significant challenges to the profession. The effective use of skills, knowledge and resources available to the physical therapist can support quality of client care. A key resource that can be used to support fiscal responsibility as well as quality client care is physiotherapist support personnel. A knowledgeable physiotherapist can assign activities that are within physiotherapist support personnel's scope of practice and skill level. Through effective supervision, the physiotherapist can enable physiotherapist support personnel to become effective members of a collaborative health care team.

**Target Population:** This session will be of interest to clinicians from a wide field of physical therapy practice environments, physiotherapy support personnel, and managers interested in enhancing quality client care while being fiscally responsible.

**Description of Supporting Evidence:** Evidence supports the use of physiotherapist support personnel in many settings, from rural communities to intensive care units. Models of supervision have been investigated. As the role of the physiotherapist continues to evolve, the need for leadership and mentorship skills in the supervision of support personnel is increasing. The educational curriculum of physiotherapists and physiotherapist assistants supports interdisciplinary partnership, through collaborative fieldwork placements and interprofessional classes.

**Description of Session Format:** Background information will be presented on Physiotherapist Support Personnel in the workplace including the expertise expected of novice and advanced students, new graduates, on-the job trained and experienced personnel. Case scenarios and small group discussions will provide the opportunity to identify strategies for supervision and for the assignment of tasks.

**Conclusions and Implications:** This presentation will provide Physiotherapists with strategies to supervise and work collaboratively with Physiotherapist Support Personnel of varying expertise levels in different health care environments. To ensure sustainability of physiotherapy services in Canada, it is crucial for the physiotherapist to have an understanding of the range of interventions that can be safely and efficiently provided by physiotherapist support personnel.

# Abstract: A002 – Spine motion on the elliptical trainer: Should we be concerned?

**Published:** 2010-07

**Authors:** JM Moreside, SM McGill

**Affiliations:** University of Waterloo, Waterloo, Ontario, N2L 3G1, Canada

**Purpose/Objectives and Rationale:** To quantify lumbar motion when using the elliptical trainer, while varying hand position, speed and stride length. **Relevance to the Physiotherapy Profession:** The elliptical trainer is often recommended as a low impact alternative to running. Lumbar motion associated with its use may be important, as some low back pain patients report relief, while others report pain exacerbation. **Materials and Methods:** 43 males (19–30 yrs) exercised on the elliptical trainer at 2 speeds: self-selected and 30% faster. Stride length was varied (18" or 26"), as was hand position (holding the handles, holding a central stationary bar, or freehand). 3D lumbar angles were calculated, using data captured with the Vicon system. **Analysis:** Repeated measures analysis of variance was performed, with speed, stride and hand position as independent variables. Bonferroni corrections were done. This was repeated for each axis of motion. **Results:** There were significant main effects (*p *< 0.0166) for hand position, speed and stride length associated with lumbar flexion and rotation. Only stride length demonstrated a main effect in side bending. In most instances, motion increased with speed and stride length, but the effect of hand position varied between conditions. **Conclusions:** The way in which an elliptical trainer is used will impact lumbar spine motion. The average range of total lumbar rotation in a 26 inch/fast/handles position was twice that of a slower 18 inch/bar orientation (28 vs.14 degrees). Side bending was greatest with 26 inch/fast/freehand (12.8 degrees), whereas lumbar flexion peaked with 26 inch/fast/bar (10.3 degrees).

# Abstract: A006 – Conservative management for osteoarthritis: Does a Hip and Knee Arthritis Assessment Centre visit make a difference?

**Published:** 2010-07

**Authors:** S Juma, JL Winter Di Cola, DM Kennedy, P Dickson, S Denis, S Robarts, J Gollish

**Affiliations:** Holland Orthopaedic and Arthritic Centre of Sunnybrook Health Sciences Centre, Toronto, ON, M4Y 1H1; Departments of Physical Therapy and Surgery, University of Toronto, Toronto, ON; School of Rehabilitation Sciences, Institute of Applied Health Sciences, McMaster University, Hamilton ON, L8S 1C7

**Purpose/Rationale and Objectives:** To understand if a Hip/Knee Arthritis Assessment Centre (AC) visit contributes to patients' self-management and ability to access community resources. In this innovative model, a comprehensive assessment is conducted to determine optimal management. Non-surgical candidates are directed to community resources and provided with a “prescription” for conservative management that follows OARSI guidelines. **Relevance to the Physiotherapy Profession:** Given the historical lack of conservative arthritis management, it is imperative to understand if the AC, which utilizes an Advanced Practice role for Physiotherapists/Occupational Therapists, effectively directs patient care. **Materials and Methods:** Purposive samples of non-surgical patients were contacted 3–10 months after their AC visit. To attain saturation of themes, three focus groups (*n *= 20) and 20 semi-structured telephone interviews were conducted by an independent researcher. **Analysis:** Transcripts were systematically coded and analyzed using a grounded theory approach. Eighty-six codes were grouped into 20 categories from which overarching themes were developed. Nvivo qualitative data management software was used for data organization. **Results:** Participants generally reported that the AC visit improved self-management, primarily through providing increased understanding of their condition and of appropriate exercise and treatment options. Participants described both individual-level barriers to self-management, such as motivation, and systemic barriers to accessing needed resources. Systemic barriers included: coordination of care by GPs, program availability, and access to high-quality physiotherapy. **Conclusions:** The AC visit contributes to patients' self-management and helps them access resources, such as physiotherapy and arthritis self-management programs, where systemic barriers do not exist. Because effectiveness is limited by individual-level and systemic barriers, a mechanism for patient follow-up may improve the efficacy of this model.

# Abstract: A008 – From continuing education to personal digital assistants: What do physical therapists need to support evidence-based practice in stroke management?

**Published:** 2010-07

**Authors:** NM Salbach, P Veinot, SB Jaglal, M Bayley, D Rolfe

**Affiliations:** Authors NMS, PV, SBJ, DR: Department of Physical Therapy, University of Toronto, 160–500 University Ave., Toronto, Ontario, M5G 1V7, Canada; Author MB: Toronto Rehabilitation Institute, 550 University Ave., Toronto, Ontario, M5G 2A2, Canada

**Purpose/Objectives and Rationale:** The objective of this study was to explore physical therapists' preferences for strategies to facilitate application of the stroke research literature in clinical practice given the lack of in-depth information on this topic. **Relevance to the Physiotherapy Profession:** Findings will inform development of interventions to facilitate provision of evidence-based physical therapy post-stroke. **Materials and Methods:** In-depth, qualitative telephone interviews were conducted with 23 physical therapists who treat people with stroke in Ontario, Canada. **Analysis:** Data were analyzed using a constant comparative approach to identify emergent themes. **Results:** Participants preferred online access to research summaries or systematic reviews to save time to filter and critique research articles. Strategies to increase understanding and appraisal of research included use of non-technical language, glossaries of research terms, and quality ratings of studies. Detailed descriptions or videos of treatment interventions were expected to facilitate their clinical implementation. An acceptable computer-to-staff ratio, permission to access websites, and protected work time were organizational supports considered necessary to access online resources. Tele- or video-conferencing was preferred to overcome geographical barriers to accessing education events. Participants considered personal digital assistants as a tool to enable quick access to online resources but questioned the feasibility of using them in daily clinical practice. To achieve behaviour change in clinical practice, therapists preferred multiple, interactive, face-to-face education sessions in a group format, with case-based learning and opportunities to practice new skills. **Conclusions:** Technology enables access to online resources and education. Therapists prefer attending multi-session, interactive, expert-facilitated education sessions incorporating opportunities for case-based learning and practice of new skills to change clinical practice behaviour.

# Abstract: A009 – The use of the P4 Pain Intensity Measure in patients with hip and knee osteoarthritis

**Published:** 2010-07

**Authors:** M Dogra, DM Kennedy, LJ Woodhouse, GF Spadoni, PW Stratford

**Affiliations:** Sunnybrook Holland Orthopaedic & Arthritic Centre, Department of Rehabilitation, Toronto, ON M4Y 1H1; McMaster University, School of Rehabilitation Science, Hamilton, ON L8S 1C7; University of Toronto, Department of Physical Therapy, Toronto, ON M5G 1V7, Canada

**Purpose/Objectives and Rationale:** To investigate the validity of a 4-item pain intensity scale (P4) in measuring pain from hip and knee osteoarthritis (OA). **Relevance to the Physiotherapy Profession:** Physiotherapists frequently assess and treat patients with advanced hip and knee osteoarthritis. Pain is a core concern that should be measured using valid tools that are feasible in busy practice settings. **Materials and Methods:** Using a construct validation design, 117 (hip* *= 54; knee = 63) arthroplasty candidates with a mean age of 65.6 (SD 11.2) years completed the P4 and Western Ontario and McMaster Universities Osteoarthritis Index (WOMAC) preoperatively. **Analysis:** Confirmatory factor analysis was applied to examine the structure of the P4. Internal consistency was estimated using Cronbach's Alpha. To evaluate construct validity, the correlation between the P4 and WOMAC pain subscales and the ability of the P4 to differentiate between patients awaiting hip and knee replacement were examined. **Results:** The confirmatory factor analysis demonstrated that the P4 assesses a single concept, pain intensity. Internal consistency (Cronbach's Alpha) of the 4 items was 0.93. The correlation between the WOMAC pain subcale and the P4 was 0.67 (95% CI: 0.56–0.76). Although the point estimate of pain was 2.1 P4 pain points greater for patients awaiting total hip replacement, this difference was not statistically different. **Conclusions:** Although establishing validity of a measure is an ongoing process, the results of this study provide evidence that the P4 can be used to reliably measure pain intensity in patients with OA awaiting joint replacement. Easy to complete, the P4 offers a standardized approach for busy clinicians.

# Abstract: A010 – Outcome measures used in non-drug, non-surgical arthritis trials: What is guiding our Clinical Practice Guidelines?

**Published:** 2010-07

**Authors:** P Fox, J Sessford, D Beaton, E Harniman, C Kennedy, T Inrig

**Affiliations:** Mobility Program Clinical Research Unit, Keenan Research Centre, Li Ka Shing Knowledge Institute, St Michael's Hospital, Toronto, ON M5B 1W8, Canada

**Purpose/Objectives and Rationale:** To examine outcomes used in non-drug, non-surgical interventions in arthritis trials. Relevance to the Physiotherapy: Clinical practice guidelines reflect the outcomes used in clinical trials but these trials may not focus on outcomes most relevant to patients with arthritis. **Materials and Methods:** We searched the Cochrane Database of Systematic Reviews for reviews of non-drug, non-surgical arthritis trials. Author and publication year, arthritis type, intervention type, and outcomes were extracted. We classified outcomes using the International Classification of Function, Disability and Health (ICF). **Analysis:** Frequencies were generated and trends over time were examined. **Results:** We found 27 reviews (from 2000 to 2008) and 196 trials were included. 55% of trials included patients with inflammatory arthritis (IA); 32% osteoarthritis (OA); and 13% both IA and OA. The interventions were exercises (29%), patient education (32%), physical modalities (24%), and splint/orthotics (16%). The percentage of trials reporting at least one measure in the following outcome categories were 65% for pain, 45% for disease specific health related quality of life, 41% for tender joint count, 40% for range of motion, and 21% for walking. Only 2 trials reported work status. 93% of trials fielded at least one outcome in ICF Body Functions and Body Structures; 52% in Activities and Participation; and 32% in Environmental Factors. For IA, the relative frequency of exercise trials increased over time. **Conclusions:** Most outcomes used in arthritis trials measure impairment. If more emphasis was placed on quality of life measures, clinical practice guidelines would focus on outcomes most relevant to patients.

# Abstract: A012 – Attentional focus and functional performance in individuals with Parkinson's Disease

**Published:** 2010-07

**Authors:** K Baxter, A Portanova, L Smuck, DM Connelly, J Shaw, A Adkin, M Jog

**Affiliations:** University of Western Ontario, School of Physical Therapy, London ON

**Purpose/Objectives and Rationale:** Those living with Parkinson's disease (PD) have an increased risk of falling secondary to balance and mobility impairments. Functional performance of individuals with PD may be influenced through use of attentional focus strategies. However, it is unclear whether an internal or external focus of attention is more beneficial. The current study compares the effect of varying attentional focus instructions on the performance of individuals with PD using clinically applicable tasks that simulate common causes of falls in individuals with PD. **Relevance to the Physiotherapy Profession:** If the use of focus instructions enhances functional task performance in the PD population it could facilitate a new approach towards fall prevention. **Materials and Methods:** Three clinical measures; Functional Reach Test (FRT), Timed Up & Go (TUG) and a 360 Turn; were completed in a randomized order by twenty-six subjects (mea*n *= 67 ± 7.5 years, 16 men). Researchers measured performance of each test under three attentional focus conditions; internal, external, or no instruction (control). Participants completed nine trials of each test, with randomized three trial blocks of each condition. **Analysis:** Mean values from each of the three conditions were calculated and compared to analyze performance within each task using one-way ANOVAs. **Results:** No statistically significant differences were found for performance under control, internal or external attentional focus for the FRT (*p *= 0.722), TUG (*p *= 0.833) or 360 Turn (*p *= 0.749). **Conclusions:** Attentional focus instructions did not influence functional performance in this study population. Further investigation is required using more challenging tasks.

# Abstract: A015 – Progressive exercise for balance impaired older adults referred for home care physiotherapy: Is it beneficial to target vestibular control and lower limb muscle strength?

**Published:** 2010-07

**Authors:** D Hollway, M Earl, A Murphy, M MacKay-Lyons, E MacDonald

**Affiliations:** MacDonald E: Dalhousie University, Faculty of Medicine, Halifax, NS B3H 4H7.; Murphy M, and MacDonald E: Saint Joseph's Hospital, Saint John, NB E2L 3L6.; * Dalhousie University, School of Physiotherapy, Halifax, NS B3H 3J5.

**Purpose/Objectives and Rationale:** To evaluate the effect of progressive vestibular exercise on the control of standing balance in older, balance impaired adults referred for public home-care physiotherapy. **Relevance to the Physiotherapy Profession:** Balance limitations can threaten the independence of seniors who are eligible for public home-care physiotherapy. Evidence is needed about how exercise can mitigate vestibular impairments and help protect or restore balance. **Materials and Methods:** Seven participants (age 75–89 years), referred to home-care physiotherapy for balance problems, volunteered. They were randomly assigned to a supervised, individual home exercise program (3 sessions per week for 8 weeks). The control group completed progressive resistive exercise (RE) of lower limb muscles. The experimental group completed the RE, plus progressive balance exercise targeting vestibular control of balance (RBE). Vestibular control of standing balance was assessed using the Clinical Test of Sensory Interaction and Balance (CTSIB) at baseline (PreEx) and after the program (PostEx). **Analysis:** The null hypothesis, that the Change in CTSIB-Test5 (PostEx score minus PreEx score) of the RE Group equalled that of the RBE Group, was tested using the Mann Whitney U-test (α = 0.05). results: The median PostEx CTSIB-Test5 scores of the RE and RBE groups were 7.8 and 30.0 seconds, respectively. The Change in CTSIB-Test5 of the RBE Group (median 23.3 s) was greater than that of the RE Group (median 0.6 s) (W = 18.0, *p *< 0.05). **Conclusions:** Control of standing balance was modified by combined progressive vestibular training and resistance exercise, but not by resistance exercise alone.

# Abstract: A019 – A survey of adult patient suctioning practice in Ontario

**Published:** 2010-07

**Authors:** TJ Overend, D Brooks, CM Anderson, L Cicutto, M Keim, D McAuslan, M Nonoyama

**Affiliations:** School of Physical Therapy, The University of Western Ontario, London, ON N6G 1H1; Department of Physical Therapy, University of Toronto, Toronto, ON M5G 1V7; London Health Sciences Centre, London, ON N6A 5W9; National Jewish Centre for Medicine and Research, Denver, CO 80206; St Joseph's Health Care, London, ON N6A 4V2; London Health Sciences Centre, London, ON N6A 4G5; Toronto Rehabilitation Institute, Toronto, ON M5G 2A2

**Purpose/Objectives and Rationale:** Our purpose was to survey Ontario physiotherapists (PTs) on their adult patient suctioning practices to 1) identify changes in practice since an earlier survey in 1997, and 2) provide background information for a new clinical practice guideline on suctioning. **Relevance to the Physiotherapy Profession:** Suctioning is a controlled act in Ontario. It is invasive and not without risk. Physiotherapists suction patients in various care settings ranging from intensive care units to home care. A survey of the suctioning practices of PTs in Ontario was last conducted in 1997; the last clinical practice guideline was published in 2001. **Materials and Methods:** A multi-disciplinary research team developed and pilot tested the survey before it was mailed to a sample of 150 randomly selected PTs who indicated on their college registration that they performed the controlled act of suctioning. Two reminders were provided. **Analysis:** Responses were analyzed using descriptive statistics. **Results:** Sixty-three surveys were returned for a final return rate of 42%. Nearly half (48%) of respondents performed suctioning during more than one shift per week. Seventy-one percent of the respondents had ≥6 years of experience in suctioning. A total of 88% either ‘never’ (18%) or only ‘sometimes’ (70%) wore a droplet protection mask when suctioning non-intubated patients. Five percent of PTs never hyperoxygenated, 10% routinely hyperinflated and 24% routinely instilled saline with the suctioning of intubated adult patients. **Conclusions:** The results show significant variation and changes in practice since the survey in 1997. The existing suctioning guidelines are not being followed in a number of areas.

# Abstract: A020 – Characteristics of children's total body movement during virtual reality video game play

**Published:** 2010-07

**Authors:** D. Levac, M. Pierrynowski

**Affiliations:** Rehabilitation Science, Institute for Applied Health Science Room 402, McMaster University, 1400 Main Street West, Hamilton ON, L8S 1C7; Rehabilitation Science, IAHS Rm 441, McMaster University, Hamilton ON, L8S 1C7

**Purpose/Objective and Rationale:** There is increasing interest in the use of commercially-available virtual reality (VR) gaming systems within rehabilitation, yet little evidence exists to substantiate the ‘active ingredients’ of this new technology. These systems interest physiotherapists because of their requirement for total body movement. Understanding the movement characteristics of typically developing children during game play may support the development of research questions exploring the potential of this technology to improve movement skills in children with motor impairments. To this effect, this study describes quantity and quality of total body movement during Nintendo Wii/WiiFit™ game play, explores differences in movement characteristics between games and between novice and experienced players, and investigates whether motivation impacts movement characteristics. **Relevance to the Physiotherapy Profession:** Quantifying the ‘active ingredients’ of new interventions is essential to physiotherapy practice and research. **Materials and Methods:** Thirty-eight children with and without previous game experience played Wii (boxing, tennis) and WiiFit (ski slalom, soccer heading) games. Force plate data provided centre of pressure path length excursion (quantity) and processed pelvis motion indicated smoothness and economy of movement (quality). Children rated their motivation to succeed at each game. **Analysis:** Analysis of variance (ANOVA), repeated-measures ANOVA, and analysis of covariance. **Results:** Movement quantity and quality differed between games (*p *< .001). Children with previous experience playing WiiFit games demonstrated greater movement quantity during WiiFit game play (*p *< .001); quality of movement did not differ between groups. Motivation did not influence the relationship between experience and outcomes. **Conclusions:** Defining total body movement characteristics during game play in typically developing children enhances clinical understanding of this technology and informs further research.

# Abstract: A025 – A land of opportunity: SWOT analysis of physiotherapy in Kuwait

**Published:** 2010-07

**Authors:** M MacPherson, L Glassman, P Jadan, L MacArthur, M Landry

**Affiliations:** University of Toronto, Department of Physical Therapy

**Purpose/Objectives and Rationale:** Since the liberation of Kuwait after the Gulf War, Kuwait has developed a highly socialized health care system which is funded completely from oil reserves. However, this rapid growth and socioeconomic development has had a negative impact on the health of its people, with the prevalence of non-communicable diseases at alarmingly high rates. The purpose of this study is to explore the current state of physiotherapy in Kuwait and provide recommendations for future development and growth. **Relevance to the Physiotherapy Profession:** The profession of physiotherapy is in a unique position to address the preventable health conditions amongst the general population in Kuwait by providing health behaviour interventions and promoting healthy lifestyles. **Materials and Methods:** Interviews of 17 key-informants (clinicians, administration, and other key-stakeholders) were completed in Kuwait, with subsequent transcription and coding of emerging themes in Toronto. **Analysis:** The SWOT framework (strengths, weaknesses, opportunities, and threats) was applied to categorize the emerging themes and provide a strategic direction for the profession. **Results:** Informants reported that strengths included funding for services and motivation of the professionals. Weaknesses included education and professional resources, professional advocacy, lack of standardization and regulation of practice. Opportunities were untapped demand for physiotherapy services, ongoing development of the physiotherapy association, and multidisciplinary collaboration. Threats included public awareness of physiotherapy, interprofessional communication, and cultural perspectives. **Conclusions:** Many unique opportunities exist for physiotherapists in Kuwait. Specifically, further development of the professional association could help with advocacy efforts for the profession, help to initiate standards of practice, and provide opportunities for professional collaboration.

# Abstract: A027 – In Vivo And In Vitro models to study the role of leukocytes in muscle regrowth from atrophy

**Published:** 2010-07

**Authors:** J Frenette, N Dumont

**Affiliations:** Département de Réadaptation Pavillon Ferdinand-Vandry Université Laval, Québec, G1K 7P4, Canada

**Purpose/Objectives and Rationale:** Inflammatory cells may positively or negatively influence muscle recovery. The purpose of this study is to determine the role of neutrophils and macrophages in muscle regrowth from atrophy. **Relevance to the Physiotherapy Profession:** The timing of intervention and the understanding of all biological and cellular events are of crucial importance to initiate rapid muscle recovery during rehabilitation. **Materials and Methods:** In vivo and in vitro models were used. Contractile properties of soleus muscles were measured in mice submitted to a hindlimb unloading and reloading protocol and depleted in neutrophils (anti-Ly6G, 1 mg/kg) or macrophages (etoposide, 15 mg/kg). Ambulatory mice were used as control. Large C2C12 myotubes were also incubated in vitro for 2 days in low serum medium and co-cultured with macrophages that contained apoptotic neutrophils (anti-inflammatory macrophages). Myotube diameter was measured in light microscopy and protein content was quantified with BCA assay. **Analysis:** The data were analyzed by one-way ANOVA followed by a Tukey a posteriori test when a significant F ratio was obtained. **Results:** While neutrophil depletion had no effect on the loss in muscle force or the time of recovery from atrophy, soleus muscle force decreased by 30% in mice depleted in macrophages compared to matched placebo at 7 and 14 days post-reloading. Alternatively, in vitro experiments showed that the presence of anti-inflammatory macrophages completely prevented myotube atrophy. **Conclusions:** These results show that leukocyte infiltration during modified mechanical loading is highly regulated and that macrophages with anti-inflammatory phenotype are important to prevent muscle atrophy while promoting muscle recovery.

# Abstract: A029 – Physiotherapy needs of individuals following stroke stratified by Functional Independence Measure Score

**Published:** 2010-07

**Authors:** JD Moreland, VG DePaul, AL DeHueck

**Affiliations:** St. Joseph's Healthcare, Hamilton, Ontario L8N 4A6; Joseph Brant Memorial Hospital, Burlington, Ontario L7S 1W7

**Purpose/Objectives and Rationale:** To determine the physiotherapy needs of individuals following stroke over a one-year period. **Relevance to the Physiotherapy Profession:** It is essential that physiotherapists anticipate needs that will occur in patients following discharge from hospital to prepare patients for their transition home and to inform health care planners. **Materials and Methods:** This was a prospective cohort study. Participants were eligible if they had been admitted to hospital due to stroke. The Functional Independence Measure was used to stratify participants into three groups: scores of < 41, 41–80 and > 80. Participants completed an interview and a quantitative survey at discharge, six months and one year. **Analysis:** Frequencies of interview responses and mean magnitude of survey responses were calculated. **Results:** There were 209 participants. Needs were greater in those with lower acute Functional Independence Measure scores, although they did not always decrease over time. The interview identified seven main concerns: motor control (16.4%–83.3%), ambulation (7.7%–37.6%), falls (7.7%–22.4%), stair climbing (7.7%–14.1%), fatigue (4.6%–15.4%), fitness (0%–9.2%) and balance (0%–5.9%). The survey identified many more needs with 10 needs corresponding to activities and participation (6%–78%), eight needs related to body structures and function (27%–80%) and four needs related to services (11%–64%). **Conclusion:** There are a large and varied number of physiotherapy-related needs experienced by individuals with stroke following discharge. Preparation for discharge should consider all components of mobility and physiotherapists need to advocate for more comprehensive community services.

# Abstract: A034 – Introducing the Spinal Cord Injury Functional Ambulation Profile (SCI-FAP): Sensitivity to change

**Published:** 2010-07

**Authors:** KE Musselman, K Brunton, JF Yang

**Affiliations:** Department of Physical Therapy, Faculty of Rehabilitation Medicine, University of Alberta, AB T6G 2G4, Canada

**Purpose/Objectives and Rationale:** To assess the sensitivity to change of a new measure of walking for incomplete spinal cord injury (iSCI)—the Spinal Cord Injury Functional Ambulation Profile (SCI-FAP). **Relevance to the Physiotherapy Profession:** The 6-minute walk test and the 10-meter walk test are commonly used to assess walking after iSCI. These measures do not assess functional walking (e.g., the ability to negotiate curbs, doors and ramps). To address the need for a measure of functional walking, we developed the SCI-FAP. The SCI-FAP involves the timed performance of 8 walking tasks frequently encountered by able-bodied individuals. It is a valid and reliable measure for individuals with iSCI. **Materials and Methods:** Eleven participants with chronic iSCI were followed as they completed 2 walking training programs—precision training and endurance training. Precision training involved stepping over obstacles and targets on the ground. Endurance training involved repetitive walking on a treadmill or over-ground. For both programs, training occurred 5 days/week for 2 months. Participants completed one program (random assignment) followed by a 2-month rest period before participating in the second program. **Analysis:** Standardized response mean (SRM) was used to assess sensitivity to change. **Results:** The SCI-FAP was the only measure sensitive to change in walking ability with precision training (SRM = -0.53). It was also sensitive to change following endurance training (SRM = -0.50), however, the 10-meter walk test was the most sensitive (SRM = 0.66). **Conclusions:** The SCI-FAP is a sensitive measure of functional walking for individuals with iSCI.

# Abstract: A035 – In-home telerehabilitation for post-knee arthroplasty: A randomized trial

**Published:** 2010-07

**Authors:** M Tousignant, H Moffet, P Boissy, H Corriveau, F Cabana, F Marquis

**Affiliations:** Research Centre on Aging, Faculty of Medicine and Health Sciences, University of Sherbrooke, 1036, Belvedere sud, Sherbrooke Quebec, J1H 4C4, Canada

**Purpose/Objectives and Rationale:** The purpose of this study was to investigate the efficacy of in-home telerehabilitation as an alternative to conventional rehabilitation services following knee arthroplasty. **Relevance to the Physiotherapy Profession:** Telerehabilitation seems to be a practical alternative to home visits by a physiotherapist for improving accessibility of rehabilitation services. **Materials and Methods:** Forty community-living elders who had knee arthroplasty were recruited prior to discharge from acute care hospitals. A randomized trial was used for this study. Telerehabilitation sessions (16 sessions) were conducted by trained physiotherapists 1) from a service center to the patient's home using H264 videoconference; and 2) by usual care homecare/outpatient clinic. Disability and function were measured in face-to-face evaluations prior to and at the end of the treatments by a neutral evaluator. **Analysis:** Descriptive statistics are presented as usual. For the effcet of the interventions, intra-Group Difference was tested with the Wilcoxon test for 2 related samples. Moreover, we tested the inter-group difference (experimental VS control) with the Mann-Whitney test for 2 independent samples. **Results:** Three participants from the experimental group were lost during follow-up as opposed to three in the control group. Clinical outcomes improved for all subjects of both groups and these improvements were sustained two months post-discharge from in-home telerehabilitation. **Conclusions:** Given that telerehabilitation is a compromise compared to face-to-face intervention, we may argue that this compromise is at least equally efficacy and has the potential to increase access to services in urban and rural areas with high speed Internet services in that way.

# Abstract: A037 – Inpatient pulmonary rehabilitation: The relationship between anxiety, depression and clinical outcome measures in people with COPD

**Published:** 2010-07

**Authors:** Megan Towns, Lauren Barrett, Catherine Darling, Michelle Lee, Isabel Aganon, Kylie Hill, Dina Brooks

**Affiliations:** (1) Department of Physical Therapy, University of Toronto, Toronto, ON; (2) Department of Respiratory Medicine, West Park Healthcare Centre, Toronto, ON

**Purpose/Objectives and Rationale:** To describe the prevalence of feelings of anxiety and depression in people with chronic obstructive pulmonary disease (COPD) admitted to inpatient pulmonary rehabilitation, using the Hospital Anxiety and Depression Scale (HADS). To explore associations between these feelings and clinical outcomes. To examine whether the Chronic Respiratory Questionnaire (CRQ) could screen for feelings of anxiety and depression. **Relevance to the Physiotherapy Profession:** Relationships between anxiety and depression and the clinical outcomes are poorly understood. A better understanding might explain why clinical outcomes correlate poorly with measures of disease severity and may guide clinicians to focus on the management of anxiety and depression as a strategy to minimize the disability characteristic of COPD. **Materials and Methods:** A chart review (*n *= 188) of people with COPD admitted to a rehabilitation hospital in Toronto, Ontario over 2 years. Data collected included the HADS, CRQ and outcomes of disease severity, dyspnea, fatigue, HRQoL and functional exercise capacity. **Analysis:** Pearson's correlation, Spearman's correlation, independent sample t-test, chi-square test. **Results:** The HADS identified feelings of anxiety (35%) and depression (37%). HADS scores were significantly correlated with the CRQ domains and total scores (range: *r* = -0.341 to -0.610, *p *< 0.01), and with measures of dyspnea (range: r = 0.263–0.277, *p *< 0.01). **Conclusions:** Feelings of anxiety and depression are common in this population. HADS subscales showed moderate to weak correlations with the CRQ. Therefore, the CRQ may not be useful in screening for feelings of anxiety and depression. The clinical application of these findings may increase the awareness of the need for screening and managing anxiety and depression in inpatient pulmonary rehabilitation programs.

# Abstract: A038 – Longitudinal recovery after an anterior cruciate ligament rupture and reconstruction: Examining a cohort and a case study

**Published:** 2010-07

**Authors:** Shawn Robbins, Greg Alcock, Monica Maly, Paul Stratford

**Affiliations:** 1 Wolf Orthopaedic Biomechanics Laboratory and School of Physical Therapy, University of Western Ontario, London, ON, Canada; 2 Fowler Kennedy Sports Medicine Clinic, London, ON, Canada; 3 School of Rehabilitation Science, McMaster University, Hamilton, ON, Canada

**Purpose/Objectives and Rationale:** To examine functional recovery in a cohort following anterior cruciate ligament (ACL) reconstructive surgery. Also, a case study will examine changes in strength and gait after ACL-rupture and reconstruction with pre-injury baseline data. **Relevance to the Physiotherapy Profession:** Deficits in function, strength and gait exist following ACL-rupture and reconstruction and the recovery pattern requires further exploration. **Materials and Methods:** Participants (*n *= 45) completed the Lower Extremity Functional Scale (LEFS), a measure of physical function, from 0 to 16 weeks following ACL-reconstruction in this longitudinal study. Pre-injury strength and gait data from a 23-year old woman was available and she was followed after ACL-rupture and reconstruction. We measured concentric knee strength and gait parameters using an isokinetic dynamometer and 3-dimensional motion analysis respectively. **Analysis:** We created a nonlinear model of change which related the LEFS scores to the weeks post-surgery for the cohort. The case study was examined graphically and using descriptive statistics (means and standard deviations). **Results:** In the cohort, the model demonstrated a rapid increase in self-report function in the first 8 weeks followed by a gradual tapering of improvement. The case demonstrated decreases in knee extensor strength 4 months post-rupture (118.0 Nm) and 13 months post-reconstruction (124.0 Nm) compared to pre-injury (142.7 Nm). Kinematic and kinetic gait abnormalities also existed following ACL-rupture. The model accurately predicted the LEFS scores from the case study. **Conclusions:** The LEFS model of change provides typical recovery data and assists in developing rehabilitation goals. Strength and gait changes following ACL-rupture might predispose patients to osteoarthritis.

# Abstract: A039 – Examining predictors of physical activity in knee osteoarthritis

**Published:** 2010-07

**Authors:** Shawn Robbins, Gareth Jones, Trevor Birmingham, Monica Maly

**Affiliations:** 1 Wolf Orthopaedic Biomechanics Laboratory and School of Physical Therapy, University of Western Ontario, London, ON, Canada; 2 Human Kinetics, University of British Columbia Okanagan, Kelowna, BC, Canada; 3 School of Rehabilitation Science, McMaster University, Hamilton, ON, Canada

**Purpose/Objectives and Rationale:** To quantify physical activity levels in people with knee osteoarthritis using an accelerometer and to explore its predictors. **Relevance to the Physiotherapy Profession:** Most people with knee osteoarthritis do not meet recommended physical activity levels. Reasons for this require further exploration. **Materials and Methods:** We measured the physical activity levels of 27 participants with knee osteoarthritis (mean age = 52.4 years, standard deviation* *= 5.8 years) over one week using an accelerometer (ActiGraph) and explored its predictors in this observational study. The dependent variable included daily activity counts which captured the intensity and frequency of physical activity. Independent variables included symptom subscale of the Knee Osteoarthritis and Outcome Score, pain self-efficacy subscale of the Arthritis Self-Efficacy scale, concentric quadriceps strength, age and average minimal outdoor temperature over the week. **Analysis:** We calculated mean daily activity counts and performed a step wise multiple regression analysis between the dependent variable and independent variables. Independent variables were included in the analysis if *p *< 0.10 and were removed if *p *> 0.15. **Results:** Mean daily physical activity was 278400 (standard deviatio*n *= 91916) activity counts. The symptom subscale, pain self-efficacy subscale and minimal temperature were the only variables that significantly contributed to explained variance [*R*^2^ = 0.44, F(3,23) = 6.13, *p *< 0.05]. **Conclusions:** These findings suggest that people with knee osteoarthritis with decreased symptoms are less physically active. They perform more physical activity if they have greater confidence in their pain management abilities and during warmer weather. These factors should be considered when designing programs to increase physical activity. Additional work is required in a larger sample.

# Abstract: A041 – Further development of the Challenge Module: Measurement of efficiency of performance

**Published:** 2010-07

**Authors:** Cheryl M. Glazebrook, F. Virginia Wright

**Affiliations:** Faculty of Physical Education and Health, University of Toronto, 55 Harbord Street, Toronto, ON M5S 2W6; Bloorview Research Institute, Bloorview Kids Rehab, 150 Kilgour Road, Toronto, ON M4G 1R8

**Purpose/Objectives and Rationale:** Presently no tools are available that identify the abilities and limitations of high functioning children with Cerebral Palsy (CP). Pilot testing of a new Challenge Module with high functioning children with CP (*n *= 6) identified the need to quantify movement efficiency. The primary goal of this study was to establish dual-criterion scoring based on successful item completion and time to complete speed-based items. **Relevance to the Physiotherapy Profession:** Many high functioning children with CP experience difficulties with advanced motor skills necessary for participation. The development of a sensitive outcome measure will help physiotherapists establish and monitor rehabilitation goals. **Materials and Methods:** Thirty-four typically-developing 4.5 to 10 year-olds completed a revised version of the Challenge Module. Its 25 items included a variety of running, skipping, jumping, ball skills and balance tasks. **Analysis:** For each item median and lower quartile times were calculated for the whole sample, older (7.5–10, *n *= 16), and younger children (4.5– <7.5, *n *= 18). These values were used as cut-points for the revised response scales. Total Challenge Module scores were determined for each group using the newly established cut-points. **Results:** The overall mean score of the younger group was 48.2 (/94) while the older group's mean score was 82.6 (/94). While clear developmental progressions were noted, the Challenge Module did not capture the high-level control many older children exhibited. **Conclusions:** To accurately capture skill development it is necessary to specify movement efficiency along with movement quality. The revised Challenge Module should be piloted with high functioning children with CP prior to large-scale validation.

# Abstract: A048 – MScPT Summer School Online

**Published:** 2010-07

**Authors:** MBA Martin, B Norton

**Affiliations:** University of Alberta, Faculty of Rehabilitation Medicine, Department of Physical Therapy, Edmonton, AB T6G 2G4

**Purpose/Objectives and Rationale:** 1) To develop and deliver three MScPT entry-level summer term academic courses using a distance format. 2) To develop faculty expertise in distance education. 3) To incorporate asynchronous and synchronous learning tools for online courses with large enrollments. **Relevance to the Physiotherapy Profession:** Access to online courses allows learning to be flexible. Clinicians and entry-level students are beginning to embrace the potential of online education. Entry-level PT programs typcially follow fulltime, yearround and intensive schedules. Allowing some course work to be completed away from campus can be rejuvenating. Alternative formats can accommodate new ways of teaching and learning. Lessons learned with PT students will inform use of online formats for professional development. **Description:** Four instructors worked collaboratively to develop three online courses for the same cohort of 77 PT students. Development began one year in advance and included instructional design, completing an online course as a student, technical design and logistical planning. The courses went live in August 2009. **Observations:** A variety of synchronous (webinar) and asynchronous (discussion rooms) type learning formats were used. Assessments were varied and included webquests, case presentations, quizzes, individual and group assignments. Courses were monitored for attendance, postings and technical issues. **Critical Assessment:** Formats used allowed instructors to observe in detail how students worked and learned together. Students reported satisfaction with the courses and appreciated option to complete courses by distance. **Conclusions:** Three MScPT courses were successfully delivered online to 77 students. Student and Instructor learnings were significant. The experience was positive for all involved.

# Abstract: A053 – Examining the accuracy of predictive equations used in stroke rehabilitation

**Published:** 2010-07

**Authors:** KD Ramsaran, ME Street, SN Syed, MT Dang, R Barclay-Goddard, P Stratford, P Miller

**Affiliations:** (1) School of Rehabilitation Science, McMaster University, Hamilton, ON; (2) School of Medical Rehabilitation, University of Manitoba, Winnipeg, MB

**Purpose/Objectives and Rationale:** To examine the predictive accuracy and clinical usefulness of the Chedoke-McMaster Stroke Assessment (CMSA) predictive equations. **Relevance to the Physiotherapy Profession:** Research regarding accuracy and clinical usefulness of the predictive equations for the CMSA is currently lacking. Information obtained from predictions can provide rehabilitation professionals with information to guide intervention and discharge planning. **Materials and Methods:** A retrospective database of 162 patients admitted for rehabilitation following stroke was used. Data was included from patients assessed at admission and discharge who had experienced a unilateral stroke. Predictive outcomes were generated using the published equations. **Analysis:** Prediction bands with 95% accuracy and shrinkage were calculated. Shrinkage was calculated by comparing reported correlation coefficients to those obtained from the current dataset. Shrinkage values are recommended to be less than 0.10 to be considered clinically useful. **Results:** Complete data was available for 74 patients, with a mean age of 65 (12) years and a mean length of stay of 45 (24) days. The shrinkage values for the six Impairment Inventory (II) dimensions ranged from -0.05 to 0.09. Shrinkage values for the Gross Motor Function, Walking Indices and the total Activity Inventory (AI) scores were 0.19, 0.24 and 0.27 points respectively. The error associated with predictive values was greater than ±1.5 stages for the II dimensions, and greater than 11 points for both AI indices. **Conclusions:** The error associated with the predictive equations limits their clinical usefulness. The important role of the CMSA as a discriminative and evaluative measure for persons with stroke remains unchanged.

# Abstract: A057 – Do the manual and manipulative therapy continuing education courses meet the learning needs of Canadian physiotherapists?

**Published:** 2010-07

**Authors:** J. Karam, M. Zettel, K. Green, D. Carter, F. Lam, C. Evans, E. Yeung

**Affiliations:** (1) M.Sc.PT; (2) University of Toronto, Faculty of Medicine, Department of Physical Therapy, 160–500 University Ave, Toronto, ON, M5G 1V7, Canada

**Purpose/Objectives and Rationale:** To describe the perspectives of physiotherapists registered in the Orthopaedic Division of the Canadian Physiotherapy Association manual and manipulative therapy continuing education courses on the mode of delivery of the course, accessibility, structure, and quality of instruction. **Relevance to the Physiotherapy Profession:** Manual and manipulative therapy continuing education courses are offered to over 4000 members of the Orthopaedic Division each year across Canada. Considerable time and financial resources are invested on the part of learners. Their perspectives are valuable in shaping new ways to deliver course content. **Materials and Methods:** Subjects included physiotherapists enrolled in manual and manipulative therapy continuing education couses across Canada between January and June 2009. A survey was administered via electronic mail to 240 course participants. **Analysis:** Descriptive statistics were calculated for responses relating to course structure and accessibility, mode of course delivery and quality of instruction. **Results:** A response rate of 37% was achieved. The most desired change (52%) was to supplement the delivery of theoretical content with electronic learning modules such as compact or digital video disc. More than 65% of participants disagreed with the use of alternative modes, such as e-learning, for the delivery of the practical content. Participants were satisfied with the current course schedule and quality of instruction. **Conclusions:** The current manual and manipulative therapy curriculum appears to be meeting the learning needs of physiotherapists in Canada. Electronic learning tools and video demonstrations were identified as potentially valuable supplements. Further research is needed to explore the perspectives of physiotherapists in geographically remote areas to determine other viable delivery options for these courses.

# Abstract: A061 – Reliability of the Wheelchair Skills Test (WST) Version 4.1 for manual wheelchair users

**Published:** 2010-07

**Authors:** NJ Lindquist, TF Magis, JE Rispin, PE Walton, RL Kirby, PJ Manns

**Affiliations:** Department of Physical Therapy, Faculty of Rehabilitation Medicine, University of Alberta, Edmonton, AB (Lindquist, Magis, Rispin, Walton, Manns) and Division of Physical Medicine and Rehabilitation, Dalhousie University, Halifax, NS (Kirby).

**Purpose/Objectives and Rationale:** The purpose of this study was to evaluate the inter-rater, intra-rater, and test-retest reliability of the Wheelchair Skills Test (WST) version 4.1. Relevance to the Physiotherapy Professison: The WST can be set up in most clinical settings and is a tool that can help to appropriately target interventions designed to improve wheelchair skills. **Materials and Methods:** Participants were videotaped as they completed WST 4.1 (30 skills) on two separate occasions.

**Analysis:** Raters scored the WSTs from the video-recordings, and each participant received a total score on performance and safety. Using those scores, inter-rater, intra-rater, and test-retest reliability were determined using intra-class correlation coefficients (ICCs). Percent agreement between raters for the individual skills was also calculated. **Results:** Eleven individuals (aged 17–66 years) who used manual wheelchairs for community locomotion, participated. The ICCs for the inter-rater, intra-rater, and test-retest reliability of the performance component were 0.855, 0.950, and 0.901 (*p *< 0.001). The safety component ICC scores were 0.061 (*p *= 0.243), 0.228 (*p *= 0.048), and 0.254 (*p *= 0.041). Percent agreement between raters of each test item ranged from 68–100%. For the performance component, 25 (83%) of the skills had rater agreement above 85%. Twenty-seven (90%) of skills for the safety component had rater agreement above 85%. **Conclusions:** Reliability of the performance component of the WST 4.1 was excellent, while the ICCs for the safety component indicate only slight to fair agreement, probably due to a ceiling effect. Additional study is needed to further evaluate the reliability of the safety component with a larger and more diverse sample group.

# Abstract: A066 – A local best practice guideline for inpatient physiotherapy treatment of patients with acute and stable chronic obstructive pulmonary disease

**Published:** 2010-07

**Authors:** MK Haller, K Marler, S Lore, B Sottana, A Crowley, V Beaudin, C Sloan

**Purpose/Objectives and Rationale:** To create a local clinical practice guideline for the physiotherapy treatment of hospitalized patients with acute exacerbations of chronic obstructive pulmonary disease (AECOPD)(Part A), and stable chronic obstructive pulmonary disease (COPD)(Part B). **Relevance to the Physiotherapy Profession:** Physiotherapy referrals are frequently received for medical patients who are diagnosed with AECOPD or stable COPD. A review of the literature did not reveal a best/clinical practice guideline. **Materials and Methods:** MEDLINE and CINAHL were searched from 2003–2008 using the key words manual physical/physiotherapy and AECOPD for Part A and from 1998–2008 using the key words chest physiotherapy, stable COPD, exercise prescription and education for Part B. Only meta-analyses, systematic reviews or clinical practice guidelines in English were selected. **Analysis:** Articles were reviewed using the CASP or AGREE tool by two separate evaluators who had to reach a consensus for article acceptance. **Results:** Quality of the literature for AECOPD physiotherapy treatment is limited. We do not recommend manual techniques for AECOPD, unless there is a problem with secretion retention. Forced expiratory techniques (FET), deep breathing and coughing are recommended. For stable COPD patient treatment, strength training yields better results than endurance training. Interval training, and high and low intensity aerobic exercise all yield positive results. **Conclusion:** FET, deep breathing and coughing along with relaxation and positioning, are the first choices for physiotherapy treatment of AECOPD. Rehabilitation of stable COPD patients needs to incorporate strength training as well as endurance training. Early rehabilitation can begin in the inpatient setting, with appropriate referrals to outpatient respiratory programs.

# Abstract: A069 – Standing in an unstable MBT shoe increases activity of selected muscles crossing the ankle joint complex

**Published:** 2010-07

**Authors:** SC Landry, BM Nigg, KE Tecante

**Affiliations:** University of Calgary, Human Performance Laboratory, Faculty of Kinesiology, Calgary, AB T2N 1N4, Canada

**Purpose/Objectives and Rationale:** To determine if standing in an unstable MBT shoe increases center of pressure excursions and muscle activity levels of small muscles crossing the ankle joint complex. **Relevance to the Physiotherapy Profession:** Shoes with stability and support features can overprotect the foot and result in inactivity of the small ankle muscles, thereby contributing to strength and functional deficits and potential injury. **Materials and Methods:** 28 healthy subjects wore an unstable MBT shoe for 6 weeks. Before and after the 6 weeks, a custom electromyographic (EMG) linear array measured muscle activity of muscles crossing the ankle while standing barefoot, in an unstable shoe and in a stable shoe. Balance was also assessed using a force plate that recorded center of pressure excursions. **Analysis:** A repeated measures analysis of variance and Bonferroni pair-wise comparisons determined if differences existed i) between shoe conditions and ii) between visits for the unstable shoe. **Results:** Standing in the unstable shoe increased activity of the flexor digitorum longus, peroneal muscles and anterior compartment muscles crossing the ankle. No activity differences for the larger soleus were identified between the stable and unstable shoe conditions. Center of pressure excursions were also greater standing in the unstable shoe compared to barefoot and the stable shoe. **Conclusions:** These findings suggest that the unstable MBT shoe is effective in activating the small muscles at the ankle and could have implications for strengthening and conditioning these muscles. Balance also improved with MBT usage over time and this could have a role in reducing injury.

# Abstract: A070 – Evaluation of a Balance Program to decrease fall risk in older adults attending a Community Day Program

**Published:** 2010-07

**Authors:** Pamela Albers, Heidi Devon, Lacey Nairn, Betsy Olenick, Stephanie Roach, Cathy Arnold, Cathy Watts

**Affiliations:** School of Physical Therapy, University of Saskatchewan, 1121 College Drive, Saskatoon, SK, S7N 0W3

**Purpose/Objectives and Rationale:** To investigate whether a balance program implemented at a Community Day Program would improve balance and balance confidence in older adults with cognitive impairment. **Relevance to the Physiotherapy Profession:** It has been shown that exercise with a balance component can improve balance and balance confidence in healthy older adults; however it is not clear whether this type of program if delivered to older adults with cognitive impairment would have a similar effect. **Materials and Methods:** Participants were recruited from a Community Day Program (*n *= 13). Balance and balance confidence were measured at three intervals, each five weeks apart using the Berg Balance Scale and The Activities-Specific Balance Confidence Scale. Other measurement tools included the Mini Mental State Exam and a demographic questionnaire completed prior to baseline testing. The intervention, added after the first testing interval, involved changing the standard exercises in the exercise class at the Community Day Program by adding exercises specifically targeted towards improving balance. **Analysis:** Two one-way repeated measures analysis of variance, with Mini Mental State Exam scores as a co-variate in the analysis of balance confidence change. **Results:** Two point change in the mean scores on the Berg Balance Scale between post-control and post-intervention, which is a clinically relevant change. A statistically significant difference in Activities-Specific Balance Confidence Scale scores between baseline and post-intervention (*p *= 0.046). **Conclusions:** Adding balance specific exercises to an already existing exercise class may have an impact on balance and balance confidence in a short time span of five weeks, in a community dwelling older adult population attending a day program.

# Abstract: A071 – Physical activity in Parkinson Disease

**Published:** 2010-07

**Authors:** M Wieler, CA Jones, J Allen, R Haennel

**Affiliations:** (1) Dept of Medicine; (2) Dept of Physical Therapy, University of Alberta, Edmonton, AB T5G 0B7, Canada

**Purpose/Objectives and Rationale:** To examine daily physical activity (PA) levels in Parkinson disease (PD) obtained from both self report and measurements from an activity monitor. **Relevance to the Physiotherapy Profession:** The degree of physical activity in PD has not been clearly delineated. By exploring and understanding activity levels in people with PD, we may further optimize physiotherapy interventions. **Materials and Methods:** Persons with mild to moderate PD wore a monitor on the arm which measured physical activity and energy expenditure (Sense Wear™ Pro Armband (SWA: BodyMedia, Pittsburgh, PA)) over 48 hrs. During this time, participants also completed a survey regarding demographic information, clinical symptoms, health status (Health Utilities Index Mark 2 and 3), and activity level (The Short Questionnaire to Access Health Enhancing Physical Activity (SQUASH)). **Analysis:** Descriptive analysis of armband data and SQUASH in relation to clinical symptoms and health status were completed. PA and health status were compared to normative data. **Results:** Seven community residing subjects (48–71 years of age; 3 females) with PD (Hoehn & Yahr 1–2) participated. All ambulated independently. All reported spending the majority of PA time in ‘light’ to ‘moderate’ activities confirmed by armband data (2–4.9 METS). Three of 4 participants who reported vigorous activities ( > 5METS) spent less than 15% of their PA at this level. The armband recorded vigorous activities in 5 participants; however, 3 spent a minimal amount ( < 5%) of PA at this intensity level. **Conclusions:** Activity level in a small sample of persons with mild to moderate PD was severely restricted despite having limited disease involvement.

# Abstract: A075 – Mixed methods research and best evidence for physiotherapy

**Published:** 2010-07

**Authors:** JA Shaw, DM Connelly, A Zecevic

**Purpose/Objectives and Rationale:** A variety of perspectives exist on what constitutes “best evidence” to support physiotherapy practice. This paper advocates for the perspective that best evidence will be based on best practice in physiotherapy, thus providing research evidence that takes a prioritized focus on the workings of physiotherapy clinicians in the context of improved patient outcomes. **Relevance to the Physiotherapy Profession:** The physiotherapy field is in need of “practice-based evidence” that more adequately addresses the concerns of clinicians. **Description:** This project involved a literature review of best practice and expertise in physiotherapy to identify how research can be conducted to optimally inform practice. The concept of research paradigms is discussed and the relationship between these and practice paradigms is highlighted for its importance in determining which types of evidence are used in practice. **Observations:** Mixed methods research integrates elements of qualitative and quantitative research approaches to provide evidence that addresses relevant biomedical/physical issues while considering the context and lifestyle issues that directly impact clinical outcomes. In so doing, mixed methods research provides evidence that addresses clinicians' practice concerns more adequately than either qualitative or quantitative approaches in isolation. **Critical Assessment:** Challenges to performing mixed methods research include the time and expertise required to conduct mixed methods research and the dominance of the biomedical hierarchy of research design. **Conclusions:** Mixed methods research provides an important option for developing “practice-based evidence” that more adequately reflects best practice for physiotherapists. Integrating mixed methods research into the physiotherapy evidence base is very important for developing comprehensive support for evidence-based practice.

# Abstract: A077 – Peer evaluation: What do physical therapy students think?

**Published:** 2010-07

**Authors:** V Dal Bello-Haas, L Harrision, R Kanthan

**Affiliations:** School of Physical Therapy, University of Saskatchewan, Saskatoon SK S7N 0W3, Canada; School of Physical Therapy, University of Saskatchewan, Saskatoon SK S7N 0W3, Canada; Department of Pathology and Laboratory Medicine, College of Medicine, University of Saskatchewan, Saskatoon SK, S7N 0W8, Canada

**Purpose/Objectives and Rationale:** We examined the perceptions of physical therapy students regarding peer evaluation and the influence of type of evaluation rubric on how students evaluate their peers. **Relevance to the Physiotherapy Profession:** Very little is known about peer evaluation, despite its substantial use in physical therapy programs. **Materials and Methods:** Students completed a questionnaire comprised of open-ended questions and rating scales related to perceptions of peer evaluation, and were randomly assigned to use either a descriptor or grade peer evaluation rubric in two separate courses. **Analysis:** Data were analyzed using thematic analysis. A Mann-Whitney U test was used to examine if the type of peer evaluation rubric used resulted in differences in scoring. **Results:** Thirty-eight first year Master of Physical Therapy students (mean age = 23.5 yrs; SD = 1.7) participated. Students primarily used the top scores for both the descriptor (e.g. Superior, Excellent) and grade (e.g. 90–100, 80–89) rubrics. There was no significant difference in peer evaluation scores between groups. Positive aspects of peer evaluation included: receiving feedback from a colleague, decreased anxiety, and being evaluated by someone at same learning level. Being evaluated by someone who was not expert, receiving evaluations skewed by personal relationships, subjectiveness or personal comparisons, and lack of seriousness/effort by peers were negative aspects. **Conclusions:** Students perceive peer evaluations as having positive and negative aspects. Type of peer evaluation rubric made no difference in how students evaluated their peers. More research is needed to determine how the peer evaluation process can be maximized to be meaningful for physical therapy students.

# Abstract: A081 – Validation of the falls screening and referral algorithm

**Published:** 2010-07

**Authors:** S Lawson, N Zaluski, A Petrie, J Rohs, R Parker, V Dal Bello-Haas

**Affiliations:** School of Physical Therapy, University of Saskatchewan, Saskatoon, SK S7N 0W3 Canada; School of Physical Therapy, University of Saskatchewan, Saskatoon, SK S7N 0W3 Canada; School of Physical Therapy, University of Saskatchewan, Saskatoon, SK S7N 0W3 Canada; School of Physical Therapy, University of Saskatchewan, Saskatoon, SK S7N 0W3 Canada; School of Physical Therapy, University of Saskatchewan, Saskatoon, SK S7N 0W3 Canada; School of Physical Therapy, University of Saskatchewan, Saskatoon, SK S7N 0W3 Canada

**Purpose/Objectives and Rationale:** The Falls Screening and Referral Algorithm (FSRA) is currently being used to determine fall risk and service and care recommendations. We examined the validity of the FSRA. **Relevance to the Physiotherapy Profession:** One in three older adults fall. Accurate screening is a primary step in identifying fall risk. **Materials and Methods:** Subjects completed the: Mini-Mental Status Exam, Berg Balance Scale (BBS), and FSRA components—the Elderly Falls Screening Test (EFST) and the Multi-Factor Falls Questionnaire (MFSQ). An EFST score of 0 or 1 categorizes an individual as low fall risk. The MFSQ is administered if the EFST score is 2. Based on MFSQ score individuals are categorized as medium or high fall risk. **Analysis:** Probability of falling was calculated with a logistic regression equation using BBS scores and history of imbalance (Shumway-Cook 1997). FRSA fall risk categorizations, using ESFT and MFSQ scores, were plotted against probability of falling and were compared using Spearman Rho. **Results:** Nine males and 28 females (mean age = 77.7, SD = 4.0) participated. Seventy-nine percent were categorized as low fall risk, 14% as moderate risk and 7% as high risk (FSRA). Probabilities of falling ranged from 0.04 to 0.69 for subjects categorized as low fall risk (FSRA), and ranged from 0.0 to 0.63 for those categorized as moderate fall risk. Correlations between FRSA fall risk and probability of falling were not significant (rho = 0.35, *p *= 0.06). **Conclusions:** The EFST component of the FRSA over-stratifies individuals into a low risk category. The FRSA should undergo further testing to determine its clinical utility.

# Abstract: A085 – The Work Limitation Questionnaire and Work Instability Scale: Evaluation of validity and responsiveness in chronic work-related upper extremity disorders

**Published:** 2010-07

**Authors:** JS Roy, JC MacDermid, JH Roth, R Grewal

**Affiliations:** School of Rehabilitation Science, McMaster University, IAHS, 1400 Main Street West,Hamilton, Ontario, Canada, L8S 1C7

**Purpose/Objectives and Rationale:** Presenteeism reflects the phenomenon of decreased productivity when attending work when sick or working through injury. The Work Limitations Questionnaire (WLQ-25) and Work Instability Scale (WIS) are two presenteeism scales that have been used to measure disabilities in workers with musculoskeletal disorders. A systematic review has shown that important psychometric properties still needed to be established for these scales. Therefore, the objective of this study was to enhance existing data on the validity of the WLQ-25 and WIS, and to establish their responsiveness to change. **Relevance to the Physiotherapy Profession:** Presenteeism scales can give indications of patient's ability to work, help to monitor progress during rehabilitation and measure the changes in work limitation. **Materials and Methods:** One-hundred and sixty patients with work-related upper extremity disorders were evaluated using self-reported questionnaires at their initial visit and 6 months thereafter. Questionnaires completed at each evaluation included: WLQ-25, WIS, QuickDASH, and Chronic Pain Grade. At 6-months, patients completed global rating of change questions. **Analysis:** Standardized response mean and effect sizes were used to assess responsiveness; construct validity was assessed using known group differences (ANOVA) and Pearson correlations. **Results:** The WLQ-25 and WIS correlated with pain and disability scales (0.36 < *r* < 0.69) and discriminated between patients in different functional categories (*p *< 0.005). The WLQ-25 was largely responsive (effect size = 0.95) for improved patients, while the WIS was moderately responsive (effect size = 0.70). **Conclusions:** The WLQ-25 and WIS are able to discriminate between functional outcome subgroups and detect improvement or worsening over time in patients with work related upper extremity disorders.

# Abstract: A086 – Translating evidence to practice: Developing a framework for stroke rehabilitation in the home environment

**Published:** 2010-07

**Authors:** Alexis Officer, Julie Richardson, Vincent DePaul, Jackie Bosch, Lori Letts, Seanne Wilkins, Laurie Wishart

**Affiliations:** McMaster University; McMaster University; McMaster University; McMaster University; McMaster University; McMaster University; McMaster University

**Purpose/Objectives and Rationale:** To describe a knowledge translation process to adapt evidence into a framework for self-management and functional task-oriented rehabilitation for persons with stroke in the home environment. **Relevance to the Physiotherapy Profession:** It is likely that rehabilitation delivered in the home post acute stroke promotes motor recovery and community reintegration, however there is currently no explicit framework for delivering these services. **Description:** A Knowledge-to-Action Cycle (Graham 2006) was used to develop a process to translate best-available evidence and tacit knowledge into an instrumental approach for stroke rehabilitation in the home. This approach was founded on evidence-based motor learning principles and self-management strategies. Researchers, rehabilitation service providers, educators and administrators engaged in an iterative process to capture and adapt knowledge, assess barriers and supports, and develop training materials for this approach. Thirteen Physiotherapists and Occupational therapists were exposed to multiple learning opportunities including access to an online Learning Management System, face-to-face training with persons with stroke, and interdisciplinary mentoring from experts. **Observations:** Focus groups and telephone interviews provided information on perceived barriers and supports. Videotaped training sessions, website use statistics, and feedback from therapists were used to assess the response to the process and adapt further training. **Critical Assessment:** Challenges to implementing this approach were identified during the knowledge translation process, including constraints of the current health care delivery system and the level of training and support required by therapists. **Conclusions:** The Knowledge-to-Action process resulted in a framework for rehabilitation service delivery that will be used as a prototype to be tested in a randomized controlled trial in the future.

# Abstract: A088 – Transforming models of care in the assessment and management of balance, mobility and fall risk after stroke: Research in ‘everyday’ practice

**Published:** 2010-07

**Authors:** EL Inness, K Brunton, A Mansfield, L Biasin, S Prajapati, B Lakhani, R Mileris, M Bayley, WE McIlroy

**Affiliations:** (1) Toronto Rehabilitation Insititute, 550 University Ave., Toronto, ON M5G2A2; (2) University of Toronto, Department of Physical Therapy & Graduate Department of Rehabilitation Science; (3) Heart & Stroke Foundation Centre for Stroke Recovery, Sunnybrook Health Sciences Centre, 2075 Bayview Ave., Toronto, ON M4N 3M5

**Purpose/Objectives and Rationale:** To reduce fall risk and improve independent mobility post-stroke by accelerating the integration of advanced research techniques and findings into practice. **Relevance to the Physiotherapy Profession:** Stroke significantly impacts on safe and independent walking. Few individuals regain independence in the community and up to three-quarters of stroke survivors fall after discharge from rehabilitation. Advances in technology and understanding have the potential to advance clinical practice but require new models of care to facilitate knowledge translation. **Description:** An innovative Clinic integrates researchers and clinicians as a patient care team. The Clinic assessment integrates advanced technology (e.g. forceplates, accelerometers, pressure-sensitive mats) with clinical measures. Assessments are part of routine care; Stroke Service inpatients are assessed at admission, discharge and follow-up. Clinicians and researchers collaboratively review the patient assessment, develop a shared understanding of the patient-specific factors influencing balance, mobility or fall risk and identify appropriate therapeutic interventions. **Observations:** Change in clinician perceptions, clinical practices and patient balance and mobility outcomes will be evaluated. **Critical Assessment:** Thirty patients have been assessed since Clinic implementation. Clinic assessments have been informative to physiotherapist practice, specifically: i) forceplate measures provide details of patients' perturbation-evoked balance responses, and; 2) accelerometers provide remote assessments of patient quality, quantity and intensity of walking outside of formal observation. **Conclusions:** Research developments are facilitated through the Clinic, applied at the level of the individual patient and part of ‘every day’ clinical practice. The lessons learned from the Clinic will advance new models of balance and mobility assessment within our organization and potentially other organizations and patient populations.

# Abstract: A091 – The abiotic effects of UVC on an anaerobe and a yeast

**Published:** 2010-07

**Authors:** RJ Henderson, J Vanik, N Alyoshkina, C Diamond, J Lee, K Martinov

**Affiliations:** Rosalind Franklin University of Medicine and Science, North Chicago, IL 60010

**Purpose/Objectives and Rationale:** This study investigated the abiotic effect of UVC light on mature colonies of Clostridium perfringens, Candida albicans and a mixed culture of Candida Albicans and Staphylococcus aureaus in a simulated wound. **Relevance to the Physiotherapy Profession:** Increasing numbers of organisms are resistant to pharmacologic treatment, suggesting desirablility of UVC as an alternative treatment. This study is first to investigate the effect of UVC on Clostridium perfringens. In vivo conditions were simulated using virulent organisms, a mixed culture and intervening simulated biological tissue. **Materials and Methods:** Organisms were purchased and rehydrated according to the MdFarland standard and streaked onto blood agar plates. Organisms matured for 24 hours. Two conditions were used; direct exposure and exposure through a thin layer of parafilm to simulate eschar or granulation tissue. Organisms were irradiated for 30 and 60 seconds at a distance of 1.5 inches. Control plates were not irradiated. **Analysis:** Visual analysis established colony counts. Percent kill rates were calculated and Chi-square analysis was used determined statistically significant differences in kill rates. **Results:** For the direct exposure condition statistically significant differences were found between control and both exposure times (*p *< .0001) and 30 v. 60 second exposures (*p *= .0044) for Clostridium perfringens. For the parafilm condition there was no significant difference control v. 30 second exposure (*p = *.5061), however there was for control v. 60 second exposure (*p *= .0032) and 30 v. 60 seconds (*p *= .0203). Results for Candida albicans were inclusive. **Conclusions:** Results of this study demonstrated that UVC killed mature Clostridium Perfringens, at both irradation times and through simulated tissue. Kill rates increased with increased UVL exposure time.

# Abstract: A092 – Developing a new HIV Disability Questionnaire: A community-integrated approach

**Published:** 2010-07

**Authors:** KK O'Brien, R Alexander, K King, J Murray, M Tebeje, AM Bayoumi, T Bereket, M Swinton, G Norman, P Solomon

**Affiliations:** (McMaster University); (Prisoner's with HIV/AIDS Support Action Network); (Canadian Working Group on HIV and Rehabilitation); (Ontario Ministry of Health and Long Term Care); (Voices of Positive Women); (St. Michael's Hospital); (McMaster University, St. Michael's Hospital); (McMaster University); (McMaster University); (McMaster University)

**Purpose/Objectives and Rationales:** To develop a new HIV disability questionnaire (HDQ) using a community-integrated approach. **Relevance to the Physiotherapy Profession:** An HDQ may be used by health providers to describe the types of disability adults living with HIV experience. **Materials and Methods:** A group of researchers and community members developed a draft HIV Disability Questionnaire (HDQ) for adults living with HIV. We pooled items from existing instruments based on dimensions of disability in the Episodic Disability Framework. This framework was developed in an earlier phase of research from the perspective of adults living with HIV. **Analysis:** Community members met on two occasions to provide input on the HDQ development in the following areas: 1) questionnaire content (including generating new items where needed), 2) refinement of items, response sets and instructions, 3) timeframe of the questionnaire, 4) how to capture the episodic nature of disability, and 5) how the HDQ can be used by people living with HIV, clinicians, AIDS service organizations and policy makers. **Results:** The draft HDQ consisted of 69 items that describe the presence, severity and episodic nature of disability in four domains: symptoms/impairments (35 items), uncertainty (15 items), difficulties with day-to-day activities (8 items), and challenges to social inclusion (11 items). Community members were involved in all stages of the HDQ development. Next steps include administering the HDQ with adults living with HIV and HIV clinicians to assess its sensibility (face and content validity and ease of usage). **Conclusions:** We believe this community-integrated approach will enhance the content validity, feasibility, and applicability of the HDQ for use in the HIV community.

# Abstract: A093 – The effects of a group exercise and education program on physical function and health status in Canadian Armed Forces personnel with chronic low back pain (CLBP)

**Published:** 2010-07

**Authors:** S Anstey, M Bethune, A Thomas, E Wood-Salomon, S Curwin

**Affiliations:** Dalhousie University, School of Physiotherapy, Forrest Building, 5869 University Ave, Halifax, NS B3H 3J5, Canada

**Purpose/Objectives and Rationale:** To determine the efficacy of a group exercise and education intervention in a military population experiencing chronic low back pain (CLBP). **Relevance to the Physiotherapy Profession:** CLBP, one of the most common musculoskeletal conditions, is frequently treated by physiotherapists. The best interventions and outcome measures for CLBP remain uncertain. Research suggests that a combination of exercise and cognitive-behavioural therapy may be most beneficial. **Materials and Methods:** 28 subjects with CLB*P *> 6 mo. duration, after completing a course of individual physiotherapy, participated in a 6-week exercise and education program involving two 1.5 hour sessions per week taught by two physiotherapists. Back specific (stabilization) and general (aerobic, flexibility, strengthening) exercises were performed. Outcome measures included: a. physical (trunk endurance, chair stand test; b. functional (Patient Specific Functional Scale (PSFS), Back Performance Scale, work disability); c. health and quality of life (SF-36, Roland Morris Disability Questionnaire (RMDQ)); d. attitudes and beliefs (Tampa Scale of Kinesiophobia); e. pain (Visual Analogue Scale); and f. patient satisfaction. **Analysis:** Paired one-tailed t-tests at 0 and 7 weeks. **Results:** Significant improvements were seen (*p *< .001) in: trunk endurance, chair stand test, PSFS, BPS, physical component of the SF-36, TSK, and VAS. No changes were observed in the RMDQ. Fifty percent (14) of subjects were working at full duties, and additional two subjects returned to full work duties. Patient satisfaction was 77% (good to very good). **Conclusions:** Improvements in pain, function and physical health occurred after 6 weeks of twice-weekly exercise and education, in patients with longstanding low back pain and physically demanding employment.

# Abstract: A094 – Tremor during movement correlates well with disability in people who have essential tremor

**Published:** 2010-07

**Authors:** SN D'Amboise, ME Héroux, G Pari, KE Norman

**Affiliations:** School of Rehabilitation Therapy; * Dept of Neurology, Queen's University, Kingston, ON K7L 3N6

**Purpose/Objectives and Rationale:** Many individuals with essential tremor (ET) report disability due to their tremor; however, it is unclear whether disability correlates to tremor severity. The purpose was to determine which measures of tremor severity correlate to disability experienced by people with ET. **Relevance to the Physiotherapy Profession:** Identifying the clinical measures of disability that correlate to severity in ET will allow physiotherapists to assess progression and severity of ET without elaborate technology. **Materials and Methods:** Subjects with ET (*n *= 22) participated. Disability was evaluated using the Test Évaluant la Performance des Membres supérieurs des Personnes A○gées (TEMPA) and elements of the Fahn-Tolosa-Marin Rating Scale related to observation (FTM-A) or task performance (FTM-B). Tremor severity measures were calculated from recordings in which subjects exerted isometric force with wrist extensors, held a steady hand posture, or performed eccentric then concentric wrist extensor contraction. **Analysis:** Correlations among all measures were examined using Pearson's r; threshold for significance was *p *< 0.01. **Results:** All correlations of TEMPA scores to tremor measures for eccentric-concentric conditions were significant (r range: 0.539 to 0.729). All but one of the corresponding correlations of FTM-B scores were significant (r range: 0.535 to 0.661). Correlations of TEMPA and FTM-B scores to tremor measures for force and postural conditions were positive but more rarely significant. Correlations of FTM-A scores to tremor measures were weaker and even more rarely significant. **Conclusions:** Measures of tremor during movement relate well to disability and thus should be included when evaluating tremor impairment. The rehabilitation-based TEMPA appears to capture tremor-related disability.

# Abstract: A096 – En bloc control of the deep and superficial thoracic muscles in sudden loading and unloading of the trunk

**Published:** 2010-07

**Authors:** Linda-Joy Lee, Michel W. Coppieters, Paul W. Hodges

**Affiliations:** Centre for Clinical Research Excellence in Spinal Pain, Injury and Health, School of Health and Rehabilitation Sciences, The University of Queensland, Brisbane, Qld 4072 Australia

**Purpose/Objectives and Rationale:** To compare activity of the deep (multifidus/rotatores) and superficial (longissimus) thoracic paraspinal muscles in different regions of the thorax during sudden loading and unloading perturbations to the trunk, in two conditions of predictability (predictable, unpredictable), in a healthy population. **Relevance to the Physiotherapy Profession:** Understanding control of postural equilibrium and spinal stability in healthy participants is necessary to compare to populations with spinal pain and disability, and aids development of assessment and treatment modalities. **Materials and Methods:** cross-sectional study. Fine-wire electrodes recorded electromyographic (EMG) activity from the right deep (multifidus/rotatores) and superficial (longissimus) muscles at T5, T8, and T11 in ten healthy participants during predictable and unpredictable sudden loading and unloading of the trunk. EMG amplitude was calculated during 10 ms epochs for 50 ms before the onset of trunk perturbation and 150 ms after the perturbation. **Analysis:** We compared normalized EMG amplitude between conditions using a mixed design analysis of variance (ANOVA). Post-hoc testing used Duncan's multiple range test. **Results:** Deep and superficial thoracic paraspinal muscles were similarly active in both loading and unloading conditions, and similarly affected by the degree of predictability at all levels (T5, T8, T11) (*p *> 0.137). **Conclusions:** This en bloc recruitment of the thoracic paraspinal muscles contrasts previous findings of differential control between the deep and superficial muscles during rotational tasks and data for the lumbar spine, suggesting that discrete control of thoracic paraspinal muscles is related to rotational forces rather than sagittal plane forces.

# Abstract: A097 – A cueing-enriched reality obtained with the use of the Wii Fit game induced a facilitation of the corticomotor excitability and corrected the deficit in overT activities in subjects with Parkinson's Disease

**Published:** 2010-07

**Authors:** Audrey Perreault, Louis E. Tremblay

**Affiliations:** École des sciences de la réadaptation, Université d'Ottawa, Ottawa, ON K1H 8M5, Canada; Université d'Ottawa et Université du Québec à Chicoutimi (UQAC)

**Purpose/Objectives and Rationale:** This study's aim is to determine if the use of the Wii Fit's© sources of cueing has the potential to improve the modulation of motor cortex activity in patients with Parkinson's disease (PD). **Relevance to the Physiotherapy Profession:** To support the use of the Wii Fit© game as a therapeutic tool in a clinical setting with PD patients. **Materials and Methods:** 27 subjects volunteered for this study. The PD group (*n *= 14, 64.3 ± 6.8 yrs, medicated) and the healthy group (*n *= 13, 64.6 ± 7.1 yrs). The experimental protocol consisted in the measurement of corticospinal excitability with recordings of Motor Evoqued Potentials (MEP) via electrodes placed on dominant rectus femoris, tibialis anterior and soleus. MEP were recorded after Transcranial Magnetic Stimulation (TMS) (intensity at 10% over the motor threshold) with the subject executing observation of action (OBS), mental imagery (MI) and imitation tasks while watching a television screen featuring the Wii Fit© game. **Analysis:** STIMO program was used to measure the MEP amplitude facilitation peak to peak and was expressed in % of control values. One way ANOVA with repeated measures was also used. **Results:** A deficit in cortico-motor excitability facilitation with MI and OBS was noticed in the PD group. However, the combination of MI and OBS enhanced facilitation more than the simple addition of MI + OBS, but only in PD subjects. **Conclusions:** These results support the use of the Wii Fit© game as a cueing based therapeutic modality mainly in rendering the task more explicit (overt activities) for PD patients. © Wii Fit is copyrighted game by Nintendo.

# Abstract: A099 – Inter-rater reliabiltiy of three-dimensional ultrasound imaging of the pelvic floor muscles

**Published:** 2010-07

**Authors:** S Thibault-Gagnon, E Gentilcore-Saulnier, L McLean

**Affiliations:** School of Rehabilitation Therapy, Queen's University, Kingston, ON, K7L 3N6, Canada

**Purpose/Objectives and Rationale:** To evaluate inter-rater agreement in the assessment of pelvic floor muscle dimensions at the levator hiatus, using three-dimensional ultrasound imaging. **Relevance to the Physiotherapy Profession:** Recent studies have incorporated three-dimensional ultrasound imaging in the assessment of pelvic floor muscle anatomy and function in women with disorders such as urinary incontinence. However, to date the inter-rater reliability of these measures has not been reported. **Materials and Methods:** Eight healthy nulliparous women participated (mean age = 25.3). In randomized order, two investigators, each with 6 months of experience, performed ultrasound imaging of the pelvic floor muscles while each participant performed three repetitions of three tasks [rest, maximal voluntary contraction (MVC), maximal Valsalva maneuver (MVM)]. The evaluators measured the length of the pelvic floor muscles at the levator hiatus, as well as the anterior-posterior and transverse diameters, area and circumference of the hiatus. **Analysis:** Inter-rater reliability was assessed using Intra-class correlation coefficients (ICC), coefficients of variation and regression analyses. **Results:** Raters were very consistent across all tasks, with 10/15 ICC values above 0.90 (median ICC for rest, MVC and MVM = 0.95, 0.88, and 0.94 respectively). Across all tasks and both raters, the median coefficient of variation was 2.5%. Regression analyses indicated bias between the raters only on two measures: muscle length and hiatal circumference measured on MVM, and the raters showed excellent agreement (median slope = 0.955). **Conclusions:** Given the high reliability in these measures, we may proceed to investigate differences in pelvic floor muscle anatomy and function using ultrasound imaging in women with disorders including pelvic pain, incontinence, and/or pelvic organ prolapse.

# Abstract: P001 – A unique approach to the management of significant atelectasis in the ICU setting—A case study

**Published:** 2010-07

**Authors:** JL Kay

**Affiliations:** St Clare's Hospital, Physiotherapy Services, Eastern Health, St John's, NL, A1C 5B8, Canada

**Learning Objectives and Session Content:**

1. To explore the differences in ventilation patterns between negative and positive pressure breathing.
2. To provide a theoretical rationale for the basis of physiotherapy intervention to improve atelectasis in a positive pressure setting including prescriptive positioning with chest wall stretch manoeuvers.
3. To demonstrate the effectiveness of the proposed physiotherapy management approach through a case study, highlighting the continuum of care from ICU to discharge home with a home program to sustain volume recruitment.

**Relevance to the Physiotherapy Profession:** Atelectasis of varying degrees is a well known consequence of mechanical ventilation. When the exposure to this positive pressure mode of ventilation is short, such as overnight, the clinical consequences are often insignificant. When the clinical presentation necessitates more prolonged mechanical support, the volume loss may become more clinically relevant; having the potential to limit a patient's ability to wean from mechanical ventilation. We have a unique perspective to offer to facilitate and sustain volume recruitment within the interdisciplinary team.

**Target Population:** This information is of relevance to physiotherapists who work directly in intensive care settings, for those who cover critical care intermittently, such as on weekends, and for those who are involved with long term or home ventilated patient populations.

**Description of Supporting Evidence:** No formal studies have been reported to date exploring this physiotherapy approach. The rationale for the management approach of volume loss was developed by exploring the literature on positive pressure ventilation and on factors that influence alveolar expansion. Information will be presented on the differing ventilation patterns with negative and positive ventilation and on known influencing factors.

**Description of Session Format:** The presentation will involve mixed media and audience participation to experience some of the impact of compressed ventilation volumes and response to release techniques. Didactic material will cover the evidence for the influence positive pressure ventilation has on ventilation distribution patterns and the development of atelectasis. It will explore how this knowledge can be utilized to support a proposed approach to resolving the volume loss. A startling case study will be presented demonstrating the continuum of management from the ICU to discharge, including a video reinacting the PT intervention in the ICU setting while on BIpap. The patient had prolonged and profound volume loss with phrenic nerve involvement compounded by scoliosis and was being considered for withdrawal of care when a PT consult was proposed by this writer. The patient had a noticeable response in one treatment session and went on to be weaned totally from mechanical support.

**Conclusions and Implications:** Combining a knowledge of the effects of positive pressure ventilation, positioning and resistance factors has enabled a unique physiotherapy approach to resolving volume loss to be deduced and applied effectively. This has included the creation and prescriptive use of reusable wedges to sustain volume recruited during PT intervention. It is important that as physiotherapists we have a solid understanding of the factors contribuing to the development of a problem, including those attributed to areas not directly within our responsibility such as mechanical ventilation. There is a paucity of data specific to physiotherapy management in critical care. This does not mean we are limited in our ability to work effectively with complex problems around ventilation. As physiotherapists, with understanding of muscle action, ventilation patterns, tissue tension etc, we bring unique skill sets to the interdisciplinary team that can impact significantly on the immediate and future management approaches for mechanically ventilated patients.

# Abstract: P009 – Developing and implementing an exercise program for people with cancer

**Published:** 2010-07

**Authors:** O Cheifetz

**Affiliations:** Hamilton Health Sciences, Oncology Program Henderson Campus, Ward F3, 711 Concession St., Hamilton, Ontario L8V 1C3, Canada

**Learning Objectives and Session Content:**

**Learning Objectives:**

1. Participants will gain knowledge of the evidence supporting the incorporation of exercise programs for people with cancer throughout the cancer care continuum.
2. Participants will learn the importance of understanding cancer, possible cancer treatments, and how this knowledge will lead to the development of exercise safety guidelines and identifying important precautions and contraindications.
3. Participants will learn how to prescribe safe aerobic and strength exercises for people with cancer.
4. Participants will gain an understanding of potential outcome measures that may be utilized to evaluate the exercise programs.

**Session Content:** This session will provide an overview of the literature which relates to exercise and cancer. The effects of exercise on function, quality of life, survival, and cancer recurrence will be explored. The key elements for developing evidence informed, safe and effective programs will be presented along with strategies to their implementation and evaluation. Methods to prescribe safe aerobic and strength exercises will be presented. Adjustments to exercise programs, based on cancer type and stage, will be discussed and strategies for modifications will be suggested. Specific complications such as secondary lymphedema and cancer metastases will also be discussed.

**Relevance to the Physiotherapy Profession:** Along with improvements in the medical management of people with cancer, physiotherapists are more likely to encounter cancer survivors within a variety of settings. It is imperative that physiotherapists have a basic understanding of cancer and cancer treatment. It is important that therapists know how to identify “red flags” that will require modifications to the physiotherapy treatment. In addition, the ability to provide appropriate, individualized, exercise programs for people with cancer provides physiotherapists an opportunity to positively affect their clients on physical and psychological levels. Furthermore, physiotherapists have the opportunity to potentially affect cancer recurrence and survival.

**Target Population:** This presentation will appeal to, Physiotherapists who work in the public and private sector, Physiotherapist assistants, Kinesiologists, researchers and those interested in expanding their skills to an emerging field of physiotherapy.

**Description of Supporting Evidence:** Exercise has been shown to be a beneficial tool that can help people diagnosed with cancer throughout the cancer care continuum. However, the majority of survivors who are seen in the cancer centers do not receive exercise counseling. While the education on the benefits of exercise our patients receive is limited, they are motivated. Several studies have shown that individuals with cancer are interested in participating in exercise programs (Jones & Courneya, 2002) and believe they are able to participate (Jones & Courneya, 2002). It is therefore important that Physiotherapists, and other Allied Health professionals, assume an active role in educating women with breast cancer, and perhaps more importantly, their physicians. Jones et al (2004)(Jones, Courneya, Fairey, & Mackey, 2004) have demonstrated that a 30 second education session provided by Oncologists significantly improved patients' participation in physical activities. While chemotherapy has the potential to cure, or lengthen life expectancy, prescribing exercise along with chemotherapy has the potential to avoid or limit the debilitating effects of the treatment, and possibly prevent a recurrence. Furthermore, the inclusion of exercise can improve survival. The benefits to exercise include reduced pain (Alfano et al., 2007), nausea and vomiting (Lotfi-Jam et al., 2008), fatigue (Alfano, et al., 2007; Lotfi-Jam, et al., 2008; McNeely et al., 2006), improve quality of life (Lotfi-Jam, et al., 2008; McNeely, et al., 2006), blood counts (red/white blood cells and platelets) (Drouin et al., 2006; Peters, Lotzerich, Niemeir, Schule, & Uhlenbruck, 1995), bone mass density (Hadji et al., 2008), cardiac function (Knols, Aaronson, Uebelhart, Fransen, & Aufdemkampe, 2005; McNeely, et al., 2006; Wonders, Reigle, Wonders, & Reigle, 2009), and general strength (Lotfi-Jam, et al., 2008; Wonders, et al., 2009) and function (Lotfi-Jam, et al., 2008; McNeely, et al., 2006).

**Description of Session Format:** The session will consist of a presentation of relevant literature and practical steps to develop an exercise program for people with cancer. An opportunity to review and discuss variety of related forms, such as outcome measures and safety sheets, will be available. A designated time for questions and answers will be at the end of the presentation; however an ongoing interactive discussion with the audience will be encouraged.

**Conclusions and Implications:** The delivery of exercise programs for people with cancer provides physiotherapists an opportunity to demonstrate their unique ability to integrate knowledge of disease process, disease pathology, and safe exercise prescription. Physiotherapists need to understand the potential long reaching effects they can have on the life of cancer survivors. Furthermore, physiotherapists should appreciate their ability to prescribe safe, challenging, exercises for people with cancer.

# Abstract: P012 – Exercise rehabilitation in cancer: Evidence, evaluation and experiences from research in understudied cancer populations

**Published:** 2010-07

**Authors:** ML McNeely, KL Campbell, CJ Peddle, KS Courneya

**Affiliations:** Department of Physical Therapy, University of Alberta; Department of Physical Therapy, University of British Columbia; Behavioural Medicine, Faculty of Physical Education, University of Alberta; Behavioural Medicine, Faculty of Physical Education, University of Alberta

**Learning Objectives and Session Content:**

**Objectives:**

1. To provide a brief overview and update on cancer, its treatment and the evidence on the role of exercise in cancer rehabilitation.
2. To identify important considerations for safe and effective exercise testing and implementation of exercise programming for patients and survivors of cancer.
3. To illustrate, using selected case presentations of patients with lymphoma, head and neck, and lung cancer, how exercise testing and programming may differ depending on the cancer type and the individual's presenting symptoms. These cases will serve to demonstrate strategies for implementation of best practice.

**Relevance to the Physiotherapy Profession:** The growing population of cancer survivors has stimulated interest in the recovery issues of those living with and beyond a cancer diagnosis (Hewitt, 2006). Cancer and its treatment can result in impairments and disabilities ranging from visible physical effects (e.g. mastectomy, laryngectomy) to less obvious but debilitating effects such as pain and fatigue. Recent evidence suggests that survivors are unprepared to manage the chronic and often poorly understood effects that arise and persist after treatment of cancer (Rowland, 2006). Rehabilitation programs have the potential to help cancer survivors manage late and long term effects, and regain and optimize physical, psychosocial, and vocational functioning following treatment for the disease (Alfano, 2006). Physiotherapists and exercise professionals are well positioned to work collaboratively as leaders in cancer rehabilitation research and in the development of clinical exercise programs. The challenge is to develop and implement evidence-based exercise interventions that address the unique and often changing needs of the survivor through the transition from diagnosis to permanent survivorship.

**Target Population:** This session will be of interest to physiotherapists, exercise specialists, students, researchers and those interested in the rehabilitation of patients and survivors with cancer.

**Description of Supporting Evidence:** Several studies have examined physical activity and exercise behavior in cancer populations. Cancer treatment has been found to have a significantly negative effect on exercise participation that is not completely recovered post-treatment (Jones et al.,2004). Moreover, this research has shown that the percentage of cancer survivors who exercise regularly is as low as 16–20% (Jones and Courneya, 2002). Preliminary research has demonstrated that appropriately prescribed exercise training programs are associated with low complication rates and numerous beneficial effects. Exercise may provide an effective means of preventing deconditioning and improving physical functioning both during and following cancer treatment. A number of meta-analyses (Stevinson et al.,2004; Schmitz et al.,2005; McNeely et al.,2006) have summarized the effects of exercise as an intervention for patients and survivors with cancer. These reviews have concluded that there is evidence to support exercise as an intervention to improve physical fitness, physical functioning and quality of life for patients and survivors with cancer. While this evidence has been the result of trials primarily performed for patients with early stage breast cancer, given the low complication rates and numerous beneficial effects, the evidence is viewed as sufficient to support exercise as a rehabilitation intervention for individuals with cancer (Doyle et al., 2006). For long-term disease-related outcomes, observational data have shown a protective association between increased physical activity following breast cancer diagnosis and recurrence, cancer-related mortality and overall mortality (Holmes et al.,2005). A protective effect was shown for physical activity levels that met or exceeded the equivalent of four to five 30-minute sessions of brisk walking per week. Similar findings were reported in two observational studies examining physical activity and colorectal cancer (Meyerhardt et al.,2006; Meyehardt et al., 2006B). Given the rapid growth of interest in the implementation of exercise rehabilitation for patients and survivors of cancer, consideration of the most safe and effective methods for clinical exercise testing, prescription, and programming is a prominent issue (Jones et al., 2008).

**Description of Session Format:** The session will be a lecture format with an interactive component. The session will start with an overview of cancer and the treatments used to cure, control and palliate the disease (15 minutes). This will be followed by a brief review and update on the evidence from recent large scale randomized controlled trials, systematic reviews, and meta-analyses (10 minutes). The session will then focus on:

- Considerations for safe and effective exercise testing and training (15 minutes)
- Exercise guidelines for patients and survivors with cancer (10 minutes)
- Strategies to incorporate research evidence in the development of individualized exercise programs (10 minutes).

The remainder of the session will follow an interactive format using selected case presentations of patients with lymphoma, head and neck, and lung cancer (60 minutes). Presenters will share their experiences and the challenges they have faced working with patients and survivors of cancer.

**Conclusions and Implications:** Given the rapid growth of interest in the implementation of exercise rehabilitation for patients and survivors of cancer, consideration of the most safe and effective methods for clinical exercise testing, prescription, and programming is a prominent issue (Jones et al., 2008). While the majority of research to date has been done in breast cancer, our session will focus on cancer groups that are understudied in the rehabilitation field, yet are among the clinical population that physiotherapists treat in practice.

# Abstract: P014 – Feedback and motor learning of upper limb skills in neurological patients

**Published:** 2010-07

**Authors:** SK Subramanian, MF Levin

**Affiliations:** McGill University, School of Physical and Occupational Therapy, Montreal, QC H3G 1Y5, Canada; Jewish Rehabilitation Hospital site of the Centre for Interdisciplinary Research in Rehabilitation in Greater Montreal (CRIR), Laval, QC H7V 1R2

**Learning Objectives and Session Content:**

**Learning objectives:**

1. To become familiar with motor learning and associated terminology;
2. To become familiar with the basic characteristics of feedback;
3. To examine the evidence regarding the effectiveness of provision of feedback for motor learning in the upper limb in different neurological conditions.

**Session Content:** First, a general introduction will be provided on motor learning including a definition and a background perspective on factors that influence it. Different types of feedback, delivery schedules, media used to present feedback and their advantages and disadvantages will be discussed. This will be followed by a review of the studies on feedback provision in individuals with stroke, Parkinson's disease, schizophrenia and traumatic brain injury. Evidence regarding the effectiveness of feedback for improving motor learning in the upper limb and the impact of cognitive functioning on motor learning and feedback utilization will be discussed.

**Relevance to the Physiotherapy Profession:** Upper limb rehabilitation and motor learning in patients with neurological dysfunction is a primary concern in physical therapy clinical practice. The use of feedback by physical therapists needs to be standardized and outcomes considered within the wider concept of motor control and motor learning theories. A better understanding of the effectiveness of different types and delivery schedules of feedback may result in better outcomes of physical therapy interventions.

**Target Population:** The session is aimed at clinicians and researchers interested in neurological physical therapy practice.

**Description of Supporting Evidence:** The levels of evidence (PEDro scores and Sackett's levels of evidence) for the studies reviewed will be presented.

**Description of Session Format:**

1. The session will start with a presentation of the description of motor learning and motor control theories. This section of the presentation will consider various terminologies used (20 mins);
2. The second part will be a brief review of types of feedback, delivery schedules and media for feedback presentation (20 mins);
3. The third section will review the available evidence on the effectiveness of different types of feedback delivery on motor learning (30 mins);
4. Finally, a discussion of the implications and the clinical relevance of feedback as well as suggestions for directions of further research will end the session (20 min).

**Conclusions and Implications:** Feedback is an important component of motor learning. Knowledge about various aspects of feedback and the impact of cognitive functioning will help better inform clinicians about use of feedback to promote adaptive plasticity and recovery in the upper limb.

# Abstract: P019 – Objective assessment of physical performance during activities of daily living

**Published:** 2010-07

**Authors:** G Dechman

**Affiliations:** School of Physiotherapy, Dalhousie University, Halifax, NS B3H 3J5, Canada

**Learning Objectives and Session Content:**

1. To describe the 10 tasks that compose the PFP-10;
2. To discuss the validity, reliability, and sensitivity to change of the PFP-10 in healthy, older adults; and
3. To examine how the PFP-10 can be used to facilitate research and focus treatment in patients with a wide range of physical abilities.

**Relevance to the Physiotherapy Profession:** Self-reported and most currently available objective tests of physical function do not allow physiotherapists to identify the cause of functional limitations. This may decrease the effectiveness of our treatments. It may also limit a researcher's ability to detect meaningful change in response to an intervention. The PFP-10 is a simple to administer, objectively scored assessment that identifies specific areas of physical impairment relevant to the performance of ADL. This test gives physiotherapists to the ability to examine how interventions affect function and to provide focused, more efficient treatment to patients.

**Target Population:** This session will be of interest to clinicians, researchers and managers who are concerned about providing interventions that focus on specific physical impairments that limit function in daily life.

**Description of Supporting Evidence:** Clinicians and investigators use assessments of physical function to determine the effectiveness of interventions. Self-reported physical function offers a unique perspective on the patient's perception of their ability to cope in their daily lives. However, self-reported function may be inaccurate and it doesn't provide sufficient information about the type of impairment affecting performance. Thus, physical performance measures have been designed to objectively describe functional ability. Many of these measures focus on mobility or balance, or assess only a single task. Many activities of daily living (ADL) require walking only short distances and involve complex combinations of balance, strength and upper extremity movement. Therefore we require tests that are able to identify impairments that limit (ADL) and assess the effects of interventions designed to improve such function. The Physical Functional Performance Test—10 (PFP-10) was developed by Cress and colleagues and consists of a battery of 10 ADL tasks. Testing conditions and instructions for each task are standardized. Subjects are asked to perform each activity using maximal effort within the bounds of safety. The performance of each activity reflects the demands of one or more physical domains: upper body strength, lower body strength, balance and coordination, flexibility, endurance. A subject's performance is quantified using the weight that is carried, or the time or distance needed to complete the task. Each activity is scored 0-to-100 based on an empirically derived range established from data gathered on older adults with a broad range of functional abilities. Individual domain scores are added to produce a total score for the test. Domain scores can be used to direct and/or assess the effects of specific physical interventions. The PFP-10 has been shown to be a valid, and reliable measure of physical function in older adults. It is sensitive to change and has no ceiling or floor effects. The test has been used to assess physical performance in people with cardiovascular disease, Parkinson's disease, Fibromyalgia, low back pain andn total hip replacements.

**Description of Session Format:** The session will be lecture format, supplemented with video presentations that show patients performing the 10 ADL tasks in the test. There will be an opportunity for participants to discuss their views on using the PFP-10 in their clinical practice and research programs.

**Conclusions and Implications:** The PFP-10 is an easy to administer, objective and reliable measure of physical performance that is specifically relevant to ADL. Its validity has been assessed in a large group of older adults with a broad range of abilities. The total and domain scores identify the causes of poor performance and can be used to direct treatment. The PFP-10 can be used to discriminate treatment effects and advance evidence-based treatment in a wide variety of physiotherapy practice areas.

# Abstract: P021 – Pediatric pain: measurement and procedural pain management

**Published:** 2010-07

**Authors:** SM Tupper

**Affiliations:** University of Saskatchewan, Department of Community Health and Epidemiology, Saskatoon, SK, S7N 5E5, Canada

**Learning Objectives and Session Content:**

1. Discuss common biases, misconceptions and barriers to recognition and management of pediatric pain.
2. Learn about the biological, psychological and social factors that influence pain and pain expression in children.
3. Discuss challenges for pain measurement in children and review age appropriate measures of pain, including self-report, behavioural and physiologic measures.
4. Learn about methods for procedural pain management that can be incorporated into clinical practice with children.

**Relevance to the Physiotherapy Profession:** Physiotherapists approach pain from three distinct perspectives. First, pain is a clinical symptom resulting from disease or injury or, in some cases of chronic pain, it is an impairment in and of itself. This pain is measured as an outcome and monitored over the course of treatment. Measurement of pain in children is challenging due to the influence of factors such as the social context, cognitive and emotional development and motor abilities of the child. In this session we will review developmentally appropriate pain measurement tools, and discuss their use and limitations. Second, children report pain with many routine physiotherapy treatment procedures. Procedural pain in children is particularly important for physiotherapists to understand and manage because it can lead to distress and fear as well as avoidance of necessary therapeutic care. This session will review clinically appropriate methods for minimizing procedural pain in children, such as preparation of the child for an uncomfortable procedure, distraction, and a variety of physical methods. A case study will be used to stimulate discussion regarding some of the challenges of procedural pain management. Third, pain is useful as a diagnostic sign during assessment; for example, the location of discomfort during a joint assessment can indicate the likely source of pain, or guide the treatment approach for the therapist. Physiotherapists are generally well versed in pain as a diagnostic tool and while this will be acknowledged, it will not be a major component of this session.

**Target Population:** This session is relevant to all physiotherapists working with children from birth to 16 years of age. The content is appropriate for therapists working with children in all practice settings, including: general pediatrics, orthopaedics, burns, rheumatology, amputation, spinal cord injury, NICU and PICU. Physiotherapists in hospital, private practice and community settings will benefit from this session.

**Description of Supporting Evidence:** Pain in children is frequently under-recognized and under-treated. This, in part, stems from common biases and misconceptions about pediatric pain. However, there is a growing body of evidence showing that children feel pain, remember pain and can have serious long-term consequences from frequent exposure to painful procedures in childhood. The last decade has seen an increase in the literature on pediatric pain, and several organizations (American Academy of Pediatrics, Canadian Pediatric Society, Royal College of Paediatrics and Child Health, The Royal Australasian College of Physicians) have published guidelines or position statements on management of procedural pain in children. Although the physiotherapy literature is largely silent on the topic of procedural pain, there is much that can be done to reduce pain and distress from physiotherapy procedures. This session will draw on the supporting literature from medicine, nursing, psychology and dentistry to present procedural pain management methods that can be adapted to multiple physiotherapy settings. Pain in children is difficult to measure, particularly in pre-school aged children, or children who communicate only non-verbally. Although numerous unidimensional and multi-dimensional tools for measurement of pain in children exist, there are limitations to their use and interpretation. Selection of an appropriate pain measurement tool will depend on the age and cognitive and motor development of the child. In this session, the Sociocommunications Model of Pain will be discussed to highlight that pain measurement is a reflection of pain perception, pain expression, and the care-giver's interpretation of and response to the pain score.

**Description of Session Format:** This session will begin with a lecture format to give an overview of the biases and misconceptions surrounding pediatric pain, and the factors influencing pain, pain expression, and pain measurement in children. The lecture will be interspersed with large group discussion questions. Pain measurement tools will be reviewed. Management of procedural pain in children will be discussed and examples given. A case study will be presented that illustrates the challenges of procedural pain management in children. Case questions will be used to facilitate small group discussion that will then be reviewed by the whole group.

**Conclusions and Implications:** It is important for physiotherapists to understand issues related to the recognition and measurement of pediatric pain, so that pain can be adequately monitored and managed in young clients. Physiotherapy procedures can be painful for children, and this can be distressing for the child, the care-givers and the therapist. Poorly managed procedural pain can have long term consequences for the child including altered pain perception and later avoidance of necessary health care. Much can be done to minimize procedural pain and to reduce fear and distress related to pain in children during physiotherapy treatment.

# Abstract: P024 – Prognosis following acute WAD: Development, predictive validity, and clinical application of a new clinical tool

**Published:** 2010-07

**Authors:** L Levesque, H Reese, T Nailer, D Walton

**Affiliations:** Clinical Whiplash Intervention and Prognosis Research Group (c-WhIP) University of Western Ontario

**Learning Objectives and Session Content:**

1. To discuss the current conceptual model of chronic Whiplash Associated Disorder (WAD) and review prognostic literature incorporating both physical and psychosocial variables.
2. To describe the development of a comprehensive screening tool for prognostic risk factors in developing chronic WAD.
3. To report the preliminary psychometric properties of the tool.
4. To provide examples via case presentation of how such a tool may be implemented and translated into clinical practice.

**Relevance to the Physiotherapy Profession:** Chronic WAD is a widespread and costly problem. It tends to be resistant to treatment, and frequently leads to long-term disability. The Bone and Joint Task Force (Holm LW, 2008) found that the annual incidence of reported WAD in North America is likely to be at least 300 per 100,000. Cost of WAD in Canada is estimated to be in excess of $3billion annually. Whiplash type injury places a significant amount of financial burden on an already challenged funding system. This workshop will provide clinicians with the best available evidence for predicting the level and nature of the risk of non-recovery following acute WAD. Early identification of those at risk of slow or incomplete recovery would allow for diversion of resources towards more efficient interventions which should reduce the rate of non-recovery and thereby mitigate the costs of rehabilitation.

**Target Population:** This session will be of interest to a broad range of healthcare professionals involved in all aspects of Whiplash Associated Disorder.

**Description of Supporting Evidence:** Models for the development of chronic pain or disability after acute whiplash or other musculoskeletal trauma range from biological (Barnsley L, 1995; Ishikawa S, 2007) to immunological (McLean SA, 2005) through psychological (Leeuw M, 2007) and even genetic (Diatchenko L, 2005). From a risk-management perspective, physical therapists as primary care providers, must be aware of and recognize the myriad of influences on recovery after acute injury. This is especially true following an injury with symptoms as nebulous as whiplash. The evidence for this workshop will come from existing peer-reviewed scientific literature in addition to original data collected by the Clinical Whiplash Intervention and Prognosis research group (c-WhIP). This workshop will represent the first time much of this data has been presented to a large clinical group.

**Description of Session Format:** This session will be a 60 minute mixed format, part lecture and part group discussion, with opportunities for greater interaction during the case presentation. We will additionally be showing a video presentation and demonstrating the various aspects of a standardized physical assessment developed specifically for use in the assessment of acute whiplash. A clinical reasoning model will be applied to the case presentation encouraging an interactive learning experience for participants. Participants will be given a subjective examination of a whiplash patient and presenters will facilitate group discussion on relevant prognostic factors and how they may guide further evaluation and/or treatment. There will be an opportunity for “hands-on” application of the various examination tools used in the standardized physical assessment (digital algometers, sensory testing with monofilaments, cervical range of motion, key muscle testing and supine neck flexion test). The session will allow for interpretation and clinical use of relevant outcome measures that capture the psychosocial variables as represented in a biopsychosocial model of barriers to recovery following acute WAD.

**Conclusions and Implications:** Whiplash is a multi-factorial problem which requires a multi-factorial approach to assessment and treatment. As primary care providers, community-based physiotherapists should appreciate the various influences on recovery from whiplash-associated disorder, and support their own interpretations with the best available scientific evidence. This knowledge will not only lead to more efficient and effective use of rehabilitation dollars, but will also facilitate discussion with other key stakeholders, including physicians and third-party payers. The results of our research to date suggest that the reliability of many common clinical assessment procedures ranges from slight to excellent and that outcome can be predicted using simple screening tools.

# Abstract: P025 – Sensitive practice: Clinical guidelines for working with adults survivors of childhood violence

**Published:** 2010-07

**Authors:** CL Schachter, CA Stalker, E Teram, G Lasiuk

**Affiliations:** School of Physical Therapy, University of Saskatchewan; Faculty of Social Work, Wilfrid Laurier University; Faculty of Nursing, University of Alberta

**Learning Objectives and Session Content:** This session is designed for all physical therapists and students who work with adults and who have limited experience working with survivors of violence. The focus is on the content of the Handbook on Sensitive Practice for Heath Care Practitioners: Lessons from Adult Survivors of Childhood Sexual Abuse, published in mid 2009 by the Public Health Agency of Canada through the National Clearinghouse on Family Violence (and is available in English and French, free of charge to all.) Upon completion of this session, participants will be able to:

- Describe the dynamics of childhood abuse and ways that the therapeutic relationship and physical therapy practice can mimic these dynamics;
- Describe effects that childhood sexual abuse can have on health and on interactions with the physical therapist, highlighting specific difficulties of male vs female survivors;
- Apply the principles and guidelines of Sensitive Practice in participants' own clinical practice;
- Describe the basic components of an appropriate response to a client's disclosure of childhood abuse;
- Describe the application of task-specific inquiry and subsequent task-specific disclosure in their clinical practice;
- Describe the basic components of an effective response to difficult situations such as when a survivor client has experienced a flashback or reminiscence of past abuse during physical therapy assessment and treatment;
- Describe components of self-care for physical therapists when working with survivors;
- Describe ways to use the Handbook on Sensitive Practice as a reference tool for clinical practice.

**Relevance to the Physiotherapy Profession:** As many as one third of women and 14% of men are survivors of childhood sexual abuse (Bolen & Scannapieco, 1999, Briere & Elliott 2003; Finkelhor 1994). Childhood adversity “including sexual, physical, and emotional abuse” is associated with a greater risk of a wide variety of health conditions (Dallam 2009; Sachs-Ericsson et al, 2009) that are within the scope of physical therapy practice. Childhood sexual abuse remains a powerful predictor of health problems in adulthood as well as difficulties with pregnancy and childbirth even after controlling for other types of childhood interpersonal violence (Briere & Runtz 1990; Briere & Elliott 1994; Schnurr & Green 2004; Whealin 2003). Studies have demonstrated increased rates of many chronic pain syndromes (including pelvic pain, low back pain, headache) as well as other conditions (see summary Schachter et al 2009). This strongly suggests that all physical therapists who work with adults “whether they know it or not” encounter adult survivors of childhood sexual abuse and other forms of interpersonal violence on a frequent basis. Since physical therapists often do not know they are working with survivors, Sensitive Practice should be used routinely with all clients, at all times.

**Target Population:** All adult clients. The high prevalence rates of childhood violence stated above suggests that physiotherapists (working with adults) work, often unknowingly, with adult survivors on a frequent basis.

**Description of Supporting Evidence:** The principles and guidelines of Sensitive Practice, detailed in the 2009 publication of the Handbook on Sensitive Practice for Health Care Practitioners: Lessons from Adult Survivors of Childhood Sexual Abuse, were developed through a multidisciplinary collaborative research project that included over 100 male and female survivors of childhood sexual abuse, and over 300 health care practitioners from more than ten health disciplines, and counsellors/psychotherapists who work with adult survivors. The Handbook reflects the culmination of this multi-phased qualitative study. Combining grounded theory and actions research, Sensitive Practice reflects a research process that has brought survivors and clinicians together. The Handbook incorporates survivor-identified difficulties when seeing health care practitioners, and their suggestions for practice that is sensitive to their needs as survivors, with health care practitioners' clinical experience and wisdom as well as their knowledge of the health care system. To our knowledge, it is the only study of its kind to date. Sensitive Practice describes client-centered care as seen through a lens of interpersonal violence. It stresses the importance of the feeling of safety for the survivor-client, introduces nine principles that help to create the feeling of safety and offers guidelines to further assist the clinician working with survivors. Sensitive Practice: a) makes visible some commonly unnamed/invisible, yet pervasive, influences of interpersonal violence on many adult clients of physical therapists, b) assists clinicians to make sense of survivor-client difficulties during health care encounters, c) helps clinicians facilitate feelings of safety for survivors so that survivors can participate in and benefit more fully from physical therapy treatment and d) offers physical therapists guidance in addressing problems that survivors said often interfere with or complicating interactions with clinicians and thus physical therapy care.

**Description of Session Format:** This session will combine didactic presentation with large and small group discussions and reflective questions. Emphasis will be placed on active learning strategies (individual and in small groups of 3–5) to enable participants to reflect on their current practice and explore clinical applications for their individual practice. In addition, the presenter will solicit and address audience-generated scenarios that are identified as problematic in clinical practice.

**Conclusions and Implications:** Childhood violence affects health and health care encounters. Sensitive Practice describes a refinement of client-centered care for adult survivors of childhood violence that is research-based. Since physical therapists often do not know they are working with survivors of childhood violence, Sensitive Practice should be used routinely with all clients, at all times.

# Abstract: P028 – Supporting early motor development, self-care and participation of young children with Cerebral Palsy: Implications for best practice

**Published:** 2010-07

**Authors:** Doreen Bartlett, Lisa Chiarello

**Affiliations:** School of Physical Therapy, Faculty of Health Sciences, The University of Western Ontario, London, Ontario, N6G 1H1; Programs in Physical Therapy and Rehabilitation Sciences, Drexel University, Philadelphia, PA, USA 19102

**Learning Objectives and Session Content:** By the end of this 90 minute educational session, participants will be able to:

1. describe how Comprehensive Rehabilitation Outcomes Research, which is informed by the World Health Organization's International Classification of Functioning, Disability and Health, provides high-level evidence for complex, chronic conditions, in which a holistic understanding of the fixed and modifiable prognostic determinants of the outcomes of interest are necessary. Participants will also be able to describe how this approach can contribute to the development of clinical expertise.
2. use the results of our research to assist with intervention planning to support early motor development, self-care and participation of young children with cerebral palsy.

Introduction:

- description of Comprehensive Rehabilitation Outcomes Research and its utility in planning research studies and in supporting clinical decision making,
- description of the conceptual model of determinants of early motor development and participation of young children with cerebral palsy.

Supporting Early Motor Development:

- description of the variety of child factors associated with early motor development of children in different Gross Motor Function Classification System (GMFCS) levels
- determining the primary and secondary impairments that are reasonable targets for intervention to support early motor development
- determining the child factors that might assist with realistic decision making about interventions to support early motor development
- discussion of the results of our study with literature about “child readiness” and “activity focused intervention” to propose effective and efficient interventions to support early motor development.

Supporting the Development of Self-care and Participation:

- description of the variety of child factors associated with development of self-care and participation of children in different GMFCS levels
- recognition of the family, environmental, and service factors that may influence self-care participation
- discussion of the results of our study with literature about “routines-based interventions” to propose effective and efficient interventions to support self-care and participation ○ consideration of fixed factors that influence decision making
- consideration of modifiable factors that influence intervention strategies.

Discussion:

- consider how to balance the focus of intervention so that children are supported in terms of development of basic motor abilities, prevention of secondary impairments, development of self-care abilities, and participation in life's activities.

**Relevance to the Physiotherapy Profession:** Practicing pediatric physical therapists require an understanding of the prognostic determinants of outcomes of interest to children and families in order to offer the best care to optimize motor outcomes, prevent the development of secondary impairments and enhance participation of young children with cerebral palsy.

**Target Population:** Physical therapists working in the area of pediatrics.

**Description of Supporting Evidence:** The content of this session is supported by research of a longitudinal, international, multi-site study of 430 young children with cerebral palsy and their families over a period of one year. We also rely on methods inherent in Comprehensive Rehabilitation Outcomes Research as a conceptual framework to support the approach used in our study, but also as a framework for clinical decision-making and the development of expertise. Our work has its foundation in research conducted by the Ontario Motor Growth Group through CanChild Centre for Childhood Disability Research. This work includes the Gross Motor Function Classification System, The Gross Motor Function Measure, and the Ontario Motor Growth Curves. In addition, this session is informed by the research conducted by the Children's Activity and Participation Study team involving 600 children and young persons with cerebral palsy, 2 to 21 years of age, and their families.

**Description of Session Format:** We will share the preliminary results of our investigation with a primary focus on the clinical implications for practice. Accordingly, we will primarily rely on a didactic lecture format, however we will have many occasions in which we will ask for thoughts from the audience (e.g. engaging participants in proposing which of the significant child factors are reasonable targets for intervention, versus those that are better suited to assist with clinical decision making). One of our objectives is to encourage practicing physical therapists to “think about their thinking”, using the World Health Organization's International Classification of Functioning, Disability and Health as a framework. So, we will aim for engagement throughout the session.

**Conclusions and Implications:** Although our focus will be on a research project specific to young children with cerebral palsy, we believe our approach has relevance to other areas of pediatric physical therapy, and indeed to all of physical therapy practice. Based on empirical evidence, prognostic determinants that are modifiable can be targets for intervention, thus enhancing the effectiveness of services. Determinants that are fixed can assist with clinical decision-making, thus enhancing the efficiency of services. Offering effective and efficient services is expected to enhance outcomes for all clients receiving physical therapy services. We suggest that the framework suggested in Comprehensive Rehabilitation Outcomes Research can be used to organize clinical decision making and the development of expertise, even in the absence of objective research evidence.

# Abstract: P032 – Theory to practice: Developing a unique physiotherapy approach to assessment of diaphragm function

**Published:** 2010-07

**Authors:** JL Kay

**Affiliations:** St Clare's Hospital, Eastern Health, St John's,NL A1C 5B8, Canada

**Learning Objectives and Session Content:**

1. To review elements of diaphragm anatomy and physiology; laying the foundation for a unique physiotherapy perspective on assessing diaphragm mechanics
2. To introduce a modified sniff test and demonstrate how it can be applied in a clinical bedside assessment.
3. To explore two case studies to demonstrate how a more detailed clinical assessment of diaphragm function guides the development of tailored physiotherapy interventions.

The presentation will explore a means to evaluate the diaphragm including its response to loading and offloading, using a variety of outcome tools. These will include targeted observations, palpation, pulse oximetry and auscultation. Verification of the effectiveness of this bedside appraisal will be demonstrated with a videotape of a patient assessment as well as a modified sniff test under fluoroscopy.

**Relevance to the Physiotherapy Profession:** Effective ventilation is a basic requirement of human activity; the diaphragm being the prime muscle of inspiration plays a major role. Improving our ability to appraise the level of a patient's diaphragm performance has an impact on our ability to optimise their overall function. This has implications for a broad spectrum of patient populations.

**Target Population:** This material is applicable to a very wide range of clinicians; from those involved with the more traditionally defined cardiorespiratory areas of practice such as post-operative patients, to those managing patients with neurological impairments such as spinal cord injuries, to physiotherapists addressing client needs in the ambulatory setting.

**Description of Supporting Evidence:** The rationale underlying the development of this assessment approach has been extrapolated from a variety of theoretical bases; no direct physiotherapy literature exploring this area of assessment has been published. Literature will be presented on diaphragm mechanics, how this is impacted by elements such as position changes and ultimately, how this influences ventilation. The various data will be connected to clinical findings to support inferences about diaphragm performance.

**Description of Session Format:** Material will be presented in a mixed media format:

1. Audience participation: using an illustrated worksheet on the effects of positioning and determining volume of ventilation, as well as trying the sniff test manoeuver.
2. Didactic presentation of supporting data and rationale.
3. Videotaped segments including a sample of clinical assessment manoeuvers and a fluoroscopy sniff test that was modified by physiotherapy directions during the testing in radiology.

**Conclusions and Implications:** A unique approach to diaphragm appraisal is possible, enabling inferences as to the spectrum of both diaphragm activity and impact on ventilation. It can guide the direction and progression of physiotherapy intervention that improves outcomes. As physiotherapists we can define whether the diaphragm requires more tailored programs such as prescriptive positioning to off load or load the muscle, to improve its performance on a continuum of recovery. As breathing is an essential function, having effective bedside assessment tools has the potential to impact the recovery of patients in a broad spectrum of practice areas.

# Abstract: P034 – Where patient and therapist physically meet: Have we overlooked skin in manual therapy?

**Published:** 2010-07

**Authors:** Diane Jacobs, Angela Busch

**Learning Objectives and Session Content:**

**Session Content:**

1. Present an evolutionary and developmental biology context for framing pain as a means by which the nervous system protects its “organism”.
2. Integrate the neuromatrix model of pain and the biopsychosocial model with manual treatment, stressing the importance of context, and the importance of the patient retaining locus of control.
3. Introduce dermoneuromodulation (DNM):
  - Introduce the anatomy of cutaneous nervous system (slides of an anatomical dissection, use of a volunteer model)
  - Explain DNM technique of physical contact as a way of accessing the nervous system via skin receptors to downregulate pain
  - Demonstrate DMN—video, interactive session including balloons and Chinese finger trap puzzles.
4. Describe research results of a single system study exploring the effects of DNM.
5. Q&A to follow.

**Learning Objectives:**

1. Participants will gain a deeper understanding of normal human nervous system function, cutis/subcutis innervation, and the neuromatrix model in which pain is an output rather than an input.
2. Participants will understand how the skin functions as an interface between ‘organism’ and environment.
3. Participants will integrate this information as manual therapists, and appreciate the role of manual therapy as interaction with, not operation upon, another person's awake and cognizing nervous system.

**Relevance to the Physiotherapy Profession:** Use of manual therapy to treat people in pain appears to have increased in Canada over the last three decades. This workshop highlights the science base lying behind and beneath the evidence base of manual therapy. Any lasting change, e.g., downregulation of pain into the future after treatment is over, will be wrought by the patient and the patient's own nervous system/brain with whatever assistance it was able to extract from treatment. The physical therapist is a consultant, contracted by the patient, to provide safe interpersonal context and support for change, usually of a pain problem to a non- or less-pain state. Manual therapy could be viewed from a neuromatrix perspective as adding novel sensory-discriminative input to a nervous system that needs more information before it can change itself to successfully downregulate pain output. In this case, educational and kinesthetic inputs are combined. The active uptake of the new input is done by the patient and the patient's nervous system. Psychologically, it is important that the patient be made aware that he or she has the right to comment on handling or ask that mechanical forces be more comfortable. It is important that the therapist explain what the brain is trying to do with manual therapy input, and ask for feedback about the comfort level of handling. Reducing threat level from the outset helps a patient to relax, and be able focus better on the new kinesthesis to help his/her nervous system successfully change itself. The importance of skin: Skin is perhaps the most important yet most often overlooked neurological portal between a therapist applying manual treatment, and a patient with persisting pain. It is important to consider what may transpire in the brain, both consciously and non-consciously, of someone whose kinesthetic, sensorimotor body representations are being stirred by an outside agent during any type of manual therapy. Skin receives mechanical forces in manual therapy: improved understanding of the cutaneous nervous system, its neurological role both locally and within the brain representations, and its likely neurodynamic mechanics, may help manual PTs to understand it as a busy and useful nervous system interface, conveniently located, which can be kinesthetically recruited.

**Target Population:** The presentation is targeted to all physiotherapists. Anyone researching or providing manual therapy will gain a fuller understanding of pain and of how knowledgeable touch can affect treatment outcomes. It will be of particular interest to physical therapists who treat individuals with persisting pain using manual therapy.

**Description of Supporting Evidence:**

1. Where nervous systems come from: basic biological evolution science, including the concept of exaptation, exemplified by “nesting doll” framework, and considered in the context of life as “living systems aggregates,” synaptic evolution (Seth Grant).
2. Developmental biology, with emphasis on essential functional differences between ectodermal and mesodermal derivatives (from Larsen, Netter).
3. Pain science (as developed by Melzack, Wall, Moseley), neurodynamics (Butler, Shaklock), neuromatrix model (Melzack).
4. Research into skin cell capacity to transduce (Boulais & Misery), movement illusions created by skin stretch (Gandevia), tactile research (Gallace), virtual body research (Moseley and many others), research on unmyelinated afferents from skin (Olausson, Craig, Hendrickson), from subcutis structures (Bowsher).
5. Neuroscience of placebo response (Benedetti).
6. Original development and research of a system of physical contact, “dermoneuromodulation”, as a means of providing novel sensory-discriminative input to a human nervous system with the goal of helping his or her neuromatrix decrease its pain output.

**Description of Session Format:** The 90-minute session will be in lecture format with slides, a video, and interactive demonstrations. There will be time allotted for questions and answers.

**Outline:**

1. Present the evolutionary and developmental biology context for framing pain as a means by which the nervous system protects its “organism” (15 minutes).
2. Integrate the neuromatrix model of pain and the biopsychosocial model with manual treatment, including importance of context. (10 minutes).
3. Introduce dermoneuromodulation (DNM), not as just another system of manual treatment in and of itself but also as something that occurs inextricably with every other form of manual therapy:
  - Provide a visual orientation to the cutaneous nervous system by showing slides of an original anatomical dissection and some on-the-spot drawing on a volunteer model; relating neurodynamic considerations using diagrams to explain and Chinese finger trap puzzles to demonstrate. (10 minutes),
  - Explain the DNM technique as a way of accessing the nervous system via skin receptors to the goal of helping the nervous system re-learn to downregulate its pain output (10 minutes),
  - Demonstrate DMN through a video, allow some hands-on practice using simple balloon models (10 minutes).
4. Describe research results of a single system study exploring the effects of DNM (15 minutes).
5. Questions and answers (20 minutes).

**Conclusions and Implications:** Physiotherapy practitioners will compete favorably in the marketplace as manual therapists by continually upgrading and refreshing our science base to make sense of what we see clinically. As a profession we need to actively include fundamental nervous system knowledge in the hypotheses upon which we base our evidence and outcomes studies. Understanding the neurophysiology of skin and the implications of nervous system response to its handling are key to achieving not just evidence-based but also science-informed practice.

# Abstract: A001 – Biomedical and psychosocial factors associated with disability after peripheral nerve injury

**Published:** 2010-07

**Authors:** Christine B. Novak, Dimitri J. Anastakis, Dorcas E. Beaton, Susan E. Mackinnon, Joel Katz

**Affiliations:** (1) University of Toronto; (2) York University, Toronto, Ontario; (3) Washington University School of Medicine, St. Louis, Missouri

**Purpose/Objectives and Rationale:** This cross sectional study evaluated biomedical and psychosocial factors associated with disability in patients following peripheral nerve injury (PNI). **Relevance to the Physiotherapy Profession:** Recognition of the multiple factors associated with disability and pain may provide more efficacious treatment strategies and improved outcome. **Materials and Methods:** Following Ethics Board approval, adults at least 6 months following PNI were invited to participate. Assessment included questionnaires to measure: symptoms of depression, pain, cold sensitivity, pain catastrophizing and disability. **Analysis:** Disability scores and independent variables were compared with statistical analyses. Multivariate linear regression was used to evaluate the predictors of disability. **Results:** There were 158 patients (mean age 41 ± 14 years). The mean DASH score was 44 ± 22 and there was significantly higher disability in unemployed patients (*p *< 0.001) and brachial plexus injuries (*p *< 0.001). The final regression model explained 53.3% of the variance with 11 predictor variables; nerve injured (*p *= 0.001), age (*p *= 0.002), time since injury (*p *= 0.002), pain (*p *= 0.005), work status (*p *= 0.007), cold sensitivity (*p *= 0.012), pain catastrophizing (*p *= 0.022), workers' compensation/litigation (*p *= 0.040), gender (*p *= 0.086), depression (*p *= 0.089), dominant hand affected (*p *= 0.09). As single predictors, the most variance was explained by pain (26% variance, *p *<  0.001), pain catastrophizing (22% variance, *p *<  0.001) and cold sensitivity (14% variance, *p *<  0.001). **Conclusions:** Following PNI, disability was predicted by biomedical and psychosocial factors including brachial plexus injury, older age, pain, work status, time since injury, cold sensitivity and pain catastrophizing.

# Abstract: A003 – Nordic walking for geriatric rehabilitation: A randomized pilot trial

**Published:** 2010-07

**Authors:** S Figueiredo, L Finch, M Jiali, S Ahmed, A Huang, NE Mayo

**Affiliations:** 1) Faculty of Medicine, School of Physical and Occupational Therapy, McGill University, Montreal, Qc, Canada; 2) Division of Clinical Epidemiology, McGill University, Montreal, Qc, Canada; 3) Faculty of Medicine, Department of Geriatrics, McGill University, Montreal, Qc, Canada

**Purpose/Objectives and Rationale:** There is a need to identify effective interventions to promote walking capacity in seniors. This study will be the first to directly compare, using a randomized trial, Nordic Walking, a new technique, with traditional walking. The objective was to estimate the relative efficacy in improving walking capacity of Nordic walking and Overground walking for the elderly. **Relevance to the Physiotherapy Profession:** Rehabilitation professionals are charged to use evidence-based practices, and because walking independence is of key importance, strategies to promote walking capacity would be of great benefit to elders and the health care system. **Materials and Methods:** Participants randomized to Nordic Walking group (*n *= 14) were instructed on the use of the poles and used them with a therapist for 20 minutes, twice a week for six weeks; participants in the control group (*n *= 16) walked with the therapist without poles for the same amount of time. Outcomes were 6-minute walk test, gait speed, balance, pain, and leg function. **Analysis:** Baseline and follow-up values on gait speed and distance walked were compared between the two groups using effect size; relative efficacy was calculated as the ratio from it was obtained. **Results:** Nordic and Overground Walking participants improved 41 meters on the 6MWT and increased their gait speed by 0.21 m/s and 0.08 m/s, respectively. Only improvement in gait speed in the Nordic Walking group reached statistical significance, with a large effect size of 0.9. **Conclusions:** Nordic Walking is 125% more effective in improving gait speed among a rehabilitation population than Overground Walking.

# Abstract: A005 – A new look at some old friends: Rasch analysis of the Oswestry Disability Index, Dallas Pain Questionnaire, Neck Disability Index and Spinal Function Sort

**Published:** 2010-07

**Authors:** Lois Lochhead, Peter MacMillan

**Affiliations:** Rehabilitation Sciences, University of British Columbia, T325—2211 Wesbrook Mall, Vancouver, BC, V6T 2B5; University of Northern British Columbia, 3333 University Way, Prince George, BC, V2N 4Z9

**Purpose/Objectives and Rationale:** To evaluate the psychometric properties of four commonly used self-report perceived disability questionnaires using Rasch Modelling techniques. Specifically, we assessed the Oswestry Disability Index, Neck Disability Index, Dallas Pain Questionnaire and the Spinal Function Sort. **Relevance to the Physiotherapy Profession:** Physiotherapists assess the reliability of clients' subjective reports compared to demonstrated abilities with these instruments. Efficacy of treatment is often established using a change score (pre-post treatment). Measurement precision is essential to ensure accurate results. **Materials and Methods:** We analyzed an intact data set collected from 300 adults presenting for functional capacity evaluation who had completed at least one of the questionnaires. These four measures have been extensively researched in terms of reliability and validity using classical statistical analyses. Rasch analysis evaluates whether each item on the questionnaire contributes to the measurement of the desired latent trait (perceived disability) and, within each item, if all distractors contribute equally to the total score. **Analysis:** Rasch Partial Credit and Rating Scale models were used. **Results:** Rasch Analysis identified that these questionnaires do not measure a single latent trait (perceived disability). Some response levels within each item do not contribute to the overall measure. Rescaling improved the Oswestry Disability Index and the Dallas Pain Questionnaire but the Neck Disability Index and the Spinal Function Sort would need significant modification to have either perform effectively. **Conclusions:** Physiotherapists need to understand the limitations of these instruments. Rasch methods improved the measurement properties of commonly used questionnaires. Rescaled versions of the Oswestry Disability Index and Dallas Pain Questionnaire will be presented.

# Abstract: A007 – Quantification of walking specific practice in patients on an inpatient stroke unit using step activity monitors

**Published:** 2010-07

**Authors:** V DePaul, H Burridge, C Kwok, J Rosario, C Stogios, E McKay, E Moyer, L Wishart

**Affiliations:** * School of Rehabilitation Science, McMaster University, 1400 Main St W, Hamilton, ON, L8S 1C7, Canada; # St. Joseph's Healthcare Hamilton, ON; ± Hamilton Health Sciences, Hamilton, ON

**Purpose/Objectives and Rationale:** Intensive task specific walking retraining has been recommended in recent stroke rehabilitation best practice guidelines. The primary purpose of this study was to quantify the walking activity of individuals undergoing rehabilitation on an inpatient stroke unit. **Relevance to the Physiotherapy Profession:** Documentation of current walking activity patterns of patients within a stroke unit will help clinicians and researchers develop and test task specific training strategies. **Materials and Methods:** A cross-sectional observational study was conducted. A convenient sample of 15 participants with a diagnosis of a stroke were recruited from patients on an inpatient stroke unit. Demographics and functional status were collected. Single leg step activity was recorded on two weekdays and one weekend day using a StepWatch 3 step activity monitor. **Analysis:** Step activity values were doubled and presented using descriptive statistics. Non-parametric statistics were used to compare weekday and weekend step activity values, and to test associations between functional status and walking activity. A *p*-value of < 0.05 was set for statistical significance. Proportion of concurrence between time of maximal sustained activity and physiotherapy session was calculated. **Results:** Weekday daily step activity (median 1064) was significantly greater than weekend values(median 210)(*p *= 0.04). Admission FIM values were moderately correlated with weekday step activity (*r* = 0.62, *p *= 0.01). Highest sustained walking values coincided with physiotherapy sessions for 68% of weekday measures. **Conclusions:** While walking activity in a stroke unit setting was low for all measurement days, participants took significanlty more steps on weekdays versus weekends. These findings support the development and evaluation of strategies to increase walking specific practice inside and outside of supervised physiotherapy time.

# Abstract: A016 – Is daily passive muscle stretching effective at preventing losses of range of motion in adult spinal cord injured patients? A systematic review

**Published:** 2010-07

**Authors:** J Holly

**Affiliations:** The Ottawa Hospital Rehabiliation Centre, Physiotherapy Discipline, Ottawa, On K1H 8M4

**Purpose/Objectives and Rationale:** The purpose of this review is to determine current best evidence for the use of passive stretching for preventing losses of range of motion in adult spinal cord injured (SCI) patients within a rehabilitation environment. It will present a review and critique of the literature relevant to: Is daily passive muscle stretching effective at preventing losses of range of motion in adult SCI patients? **Relevance to the Physiotherapy Profession:** SCI patients are at high risk for contractures secondary to immobility and spasticity. In rehabilitation, stretching is used as a treatment to prevent decreases in range of motion and as such contracture formation. This form of treatment is very resource intense and as such, the current rehabilitation environment is starting to question its efficacy. **Materials and Methods:** The PICO strategy was used to develop the question. A systematic literature review was done to identify studies. MEDLINE, PUBMED, CINAHL, PEDro, OT seeker, Cochrane and other databases were searched. Strict inclusion and exclusion criteria were established. **Analysis:** The search revealed 16 citations. After inclusion and exclusion criteria were applied 5 randomized controlled trials were accepted. These were evaluated using the guidelines for review of quantitative studies (Law et al, 2008). **Results:** Grade D evidence exists for the use of passive stretch to prevent conractures in the adult SCI population. The reviewed studies do not suport the use of passive stretching. **Conclusions:** Clinically, this means the potential of alternative methods of maintaining range of motion such as regular frequent change in position, use of anti-spasticity drugs and electrical stimulation should be explored.

# Abstract: A026 – Changes in intersegmental spinal pathways are related to motor deficits after stroke

**Published:** 2010-07

**Authors:** Joseph-Omer DYER, Éric MAUPAS, Sibele de ANDRADE MELO, Daniel BOURBONNAIS, Robert FORGET

**Affiliations:** (1) Centre de recherche interdisciplinaire en réadaptation, Institut de réadaptation de Montréal; (2) École de réadaptation, Faculté de médecine, Université de Montréal, Québec, Canada; (3) Centre Mutualiste de Rééducation Fonctionnelle, Albi, France; (4) Université Paul Sabatier, Toulouse III, France

**Purpose/Objectives and Rationale:** To investigate whether the alterations in the modulation of soleus activity by intersegmental projections from quadriceps are related to motor deficits in hemiparesis. **Relevance to the Physiotherapy Profession:** Changes in these pathways may contribute to the motor deficits of the paretic leg. **Materials and Methods:** The modulation of soleus activity evoked by the stimulation of femoral nerve (2 x MT of quadriceps) was assessed in 14 healthy participants and in 14 stroke participants. **Analysis:** The levels of the early facilitation and of the later inhibition were assessed on soleus H reflex and volontary EMG activities. **Results:** There was an increase (Mann-Whitney U; *p *< 0.05) of the heteronymous facilitation of both soleus reflex (mean ± SEM: 78 ± 23% of control H reflex) and voluntary (212 ± 45% of control EMG) activities on the paretic side of the stroke participants compared to the control participants (30 ± 3% of control H reflex and 67 ± 15 % of control EMG. The later strong inhibition of soleus activity observed in all of the control participants was decreased in those with stroke. The facilitations of soleus reflex and voluntary activities were both inversely correlated with the levels of coordination (LEMOCOT) (Spearman *r* = -0.58; *p *= 0.03 and *r* = -0.60; *p *= 0.02, respectively) and of motor recovery (CMSA) (*r* = -0.80, *p *= 0.01; *r* = -0.55, *p *= 0.04, respectively) of the paretic leg. **Conclusions:** Changes in the heteronymous spinal modulation of soleus activity could participate to the motor deficits after stroke.

# Abstract: A028 – Clinical signs used to determine return to activity in children: An inter-disciplinary study

**Published:** 2010-07

**Authors:** I Shrier, M Boudier-Reveret, D Feldman, B Mazer

**Purpose/Objectives and Rationale:** Examine criteria that expert sport medicine clinicians use for return to activity decisions in children with musculoskeletal injuries. **Relevance to the Physiotherapy Profession:** There are no guidelines to help clinicians make return to activity decisions following injury. **Materials and Methods:** We conducted an online cross-sectional survey of certified Canadian sport medicine physicians (MDs) and rehabilitation specialists [physiotherapists (PTs) or athletic therapists (ATs)]. We asked how they would measure each of the following signs in the context of a knee injury: sport-specific skills, pain, swelling, strength, range of motion (ROM), balance. In addition, clinicians ranked the importance of each sign with respect to influencing their return to activity recommendations for each of five brief clinical vignettes. **Analysis:** Descriptive. **Results:** The overall response rate was 33.6% (464/1380) with similar rates for each profession (33.2% ATs, 31.5% MDs, 39.8% PTs). For each clinical sign, all three professions preferred the same measure to determine readiness to return to play: standardized testing for sport-specific skills, impact on function for pain, palpation for swelling, manual muscle testing for strength, visual inspection for ROM, and standing on one-leg with eyes closed for balance. In one vignette, all professions had similar responses. For the remaining four vignettes, PTs and ATs generally had similar ranking patterns, but MDs were different. Finally, pain was ranked as the #1 or #2 most important sign in all five vignettes by 41.0% of MDs, 18.1% of ATs, and 11.3% of PTs whereas sport-specific skills was chosen by 9.6% MDs, 12.0% ATs and 16.1% PTs. **Conclusions:** Our results provide the foundation for future work leading towards the development of inter-disciplinary consensus guidelines.

# Abstract: A030 – Return to work outcomes for individuals with and without objective neurological findings

**Published:** 2010-07

**Authors:** SS Rege

**Affiliations:** Moncton Sports and Orthopaedic Physiotherapy, Moncton, NB E1C9Y9, Canada

**Purpose/Objectives and Rationale:** There is a common misconception that early return to work harms the back, particularly for individuals with neurological deficits. This study compares the rehabilitation outcomes of two distinct groups of low back pain (LBP) patients, those with objective neurological findings and those with normal neurology. **Relevance to the Physiotherapy Profession:** If the neurological deficit does not directly interfere with job demands, return to work can be an achievable goal even for those with objective neurological findings. **Materials and Methods:** This was a prospective observational cohort study of acute and chronic LBP cases (*n *= 3115; objective findings group *n *= 144, normal neurology group *n *= 2971) treated non-operatively at 49 spine-care rehabilitation clinics. Outcomes assessed were: change in perceived function using the Modified Low Back Outcome Score, change in Visual Analogue Scale pain rating, change in medication usage, total treatment days and return to work rates. **Analysis:** Independent samples t-tests (alpha level of 0.05) were used to assess statistical differences between groups. **Results:** At treatment discharge, the objective findings group averaged more time in treatment (*p *< 0.017) and still had significantly fewer patients who were medication free (*p *< 0.05) or symptom free (*p *< 0.05) as compared to those with normal neurology. However, at the three month follow-up, there were no statistically significant differences in perceived function (% change 22.6 v. 21.7) or return to work rates (79.3% v. 83.7%) between groups. **Conclusions:** In spite of slower treatment response, higher medication use and less pain reduction in those with objective findings, at three months the two groups had comparable functional improvements and durable return to work rates.

# Abstract: A043 – Advanced practice physiotherapy in an urban, academic rheumatology setting: A model

**Published:** 2010-07

**Authors:** LJ Soever

**Affiliations:** Mount Sinai Hospital, Toronto, ON, M5G 1X5 Canada; University of Toronto, Faculty of Medicine, Department of Rehabilitation Science and McDonald-Blumer HM, Mount Sinai Hospital, Toronto, ON, M5G 1X5 Canada; University of Toronto, Faculty of Medicine

**Purpose/Objectives and Rationale:** The purpose of this presentation is to present a model where an advanced practice physiotherapist (APP) is utilized in a large, urban, academic, interdisciplinary rheumatology department. Relevance to the Profession: Physiotherapists have a solid knowledge base of the musculoskeletal system. With extra training, such as the Advanced Clinician Practitioner in Arthritis Care program, physiotherapists can contribute to improved management of a variety of rheumatological disorders. As new models of care emerge in our constantly changing healthcare environment, the information presented can be used to inform development of future models. **Description:** In tandem with rheumatologists, the APP provides independent assessment of new patients and follow-ups with chronic inflammatory and degenerative conditions presenting with peripheral and axial disease. In addition, the APP facilitates integrated access to care with orthopaedic surgery and rehabilitation to foster continuity of care, share knowledge, and promote an interprofessional approach to patient care. Linkages are also made with various community agencies such as The Arthritis Society. **Observations:** Utilizing the services of an APP contributes to more timely access to rheumatological care, continuity of care with the orthopaedic surgery total joint replacement team and overall patient satisfaction. **Critical Assessment:** In addition to a detailed history and physical examination, the APP provides triage, disease activity monitoring, monitoring of medication side effects, education regarding self-management and exercise, exercise prescription and recommendations and referral to community agencies and programs. **Conclusions:** The APP role has proven to add value to the care of patients with rheumatological conditions.

# Abstract: A044 – A focus group study of education for injured workers with sub-acute low back pain—More than just teaching

**Published:** 2010-07

**Authors:** Anne Fenety, Alison Hoens, Katherine Harman, Raewyn Bassett

**Affiliations:** School of Physiotherapy, Dalhousie University; Dept of Physical Therapy, University of British Columbia;; Providence Health Care, Vancouver, British Columbia, Canada; School of Physiotherapy, Dalhousie University; Faculty of Health Professions, Dalhousie University

**Purpose/Objectives and Rationale:** With the current focus on client-centered care that de-emphasizes compliance with conservative treatment (e.g., bed rest and analgesics), educational approaches that embrace an understanding of risk factors for chronic low back pain and the importance of early, active intervention have become increasingly important. We explored client education by physiotherapists in private practice who treat injured workers with subacute low back pain (SA-LBP). **Relevance to the Physiotherapy Profession:** Client education is a common physiotherapy intervention. Little has been published about the content and methods of delivery physiotherapists use to educate clients with SA-LBP. **Materials and Methods:** Seven focus group interviews were held with 49 physiotherapists. Semi-structured questions and follow-up probing questions explored treatment practices in SA-LBP. **Analysis:** Transcribed focus group data were imported into the qualitative software, ATLAS/ti. An iterative, inductive analysis was conducted, involving an in-depth examination of participants' words and phrases for patterns which were labelled, sorted and clustered into distinct and comprehensive themes. **Results:** We found that education provides continuity by tying together the separate tasks undertaken during a treatment session. Participants described this seamless education process is the most efficient type of delivery when time is valuable in the private practice setting. Education was individualized by the therapist to the client's needs as tactile and visual information was gathered throughout the treatment and/or assessment process. **Conclusions:** Client education is a powerful bridge between the injured client and the physiotherapist, built from the combination of knowledge obtained from and provided to clients.

# Abstract: A047 – Does quadriceps avoidance explain reduced knee flexion during gait in those who have undergone total knee arthroplasty?

**Published:** 2010-07

**Authors:** Jeannette M. Byrne, Stephen D. Prentice

**Affiliations:** School of Human Kinetics and Recreation, Memorial University of Newfoundland, St. John's NL; Gait and Posture Lab; Department of Kinesiology, University of Waterloo, Waterloo, ON

**Purpose/Objectives and Rationale:** Reduced knee flexion during gait has been observed following total knee arthroplasty (TKA). This altered gait pattern has been attributed to reduced quadriceps activation, but this hypothesis has yet to be proven. The aim of the current research was to examine whether reduced quadriceps activation is observable in those who walk with reduced knee flexion following TKA. **Relevance to the Physiotherapy Profession:** This research will help improve understanding of why patients exhibit altered stance phase knee motion following TKA. Such information will be of benefit to clinicians when designing treatment programs for this population. **Materials and Methods:** We examined six individuals who were on average 32 months post-TKA (the “patients”), and six healthy controls. Both patients and controls performed ten walking trials. Muscle activity from all major knee muscles, as well as ankle, knee and hip motion, was recorded. Data was examined during weight acceptance, midstance and late stance phases of gait only. **Analysis:** We used a two-way repeated measures analysis of variance (factors: condition, trial) to assess between group differences in joint motion and muscle activation. **Results:** Patients exhibited significantly reduced maximum knee flexion during weight acceptance (*p *= 0.04) and reduced total knee extension during midstance (*p *= 0.008). Quadriceps muscle activation did not differ between groups. **Conclusions:** Although patients walked with less knee flexion, on average their quadriceps activation did not differ from that of controls. These findings contradict the quadriceps avoidance hypothesis often used to explain reduced stance phase knee flexion following knee replacement. Further research is needed to better understand what alternative factors may be limiting knee motion during gait.

# Abstract: A054 – Defining adverse events in orthopaedic manual therapy: The patient perspective

**Published:** 2010-07

**Authors:** Lisa Carlesso, John Cairney, Jennifer Hoogenes

**Affiliations:** Dept. of Clinical Epidemiology and Biostatistics, McMaster University; Departments of Family Medicine & Psychiatry and Behavioral Neurosciences, McMaster University; Dept. of Clinical Epidemiology and Biostatistics, McMaster University

**Purpose/Objectives and Rationale:** To describe how patients define and interpret adverse events (AE) associated with orthopaedic manual therapy (OMT) techniques. **Relevance to the Physiotherapy Profession:** No standard definition currently exists for AE in OMT practice. Both rare, serious, and common, benign, AE are associated with OMT. A systematic review has indicated a lack of standard definitions in studies collecting AE data. Research comparing clinician reporting of AE to patients demonstrates that several differences exist. **Materials and Methods:** A qualitative descriptive design was used. We conducted interviews with patients (*n *= 12) receiving OMT. Purposive sampling occurred from physiotherapy, chiropractic and osteopathic practices. The interview guide was developed from the literature and in consultation with content and methodological expertise. Basic demographic and treatment data was collected. **Analysis:** Interviews were audiotaped and verbatim transcriptions created. Two team members analyzed the data for emerging themes using the method of content analysis. Data was coded into an operational codebook. Member checking of transcripts, referential adequacy, verification and an audit trail, were used to maintain rigor. **Results:** How patients define AE is a multifactor process. Factors include pain/symptom severity, duration, functional impact, patient expectations of the clinician and treatment, education about potential AE and the weighing of benefits and harms. These concepts differ from a previously proposed framework for defining AE that did not include the patient perspective. **Conclusions:** The patient perspective is important to consider in OMT where AE are largely subjective. Including the patient viewpoint in future processes to create standard definitions will provide a broader, client-centered foundation.

# Abstract: A056 – Photodocumentation compared with tracing as a measure of wound healing

**Published:** 2010-07

**Authors:** S Gabison, EL Nussbaum

**Affiliations:** Department of Physical Therapy, University of Toronto; Department of Physical Therapy, University of Toronto and Department Rehabilitation, Mount Sinai Hospital, Toronto, Canada

**Purpose/Objectives and Rationale:** To examine differences between two methods of measuring wounds. **Relevance to the Physiotherapy Profession:** Photodocumentation and wound tracing are commonly used to evaluate treatment effectiveness. Clinical decision-making is based on the premise that wound area reduction of 30% or more over a 4-week period is indicative of wound healing. Measurement error may improperly influence clinical decision-making. **Materials and Methods:** Two observers measured 23 ulcers on the pelvis or lower extremity using different methods. Observer A traced wounds on an acetate sheet; Observer B traced wound photographs. Wounds were measured weekly until wound closure or hospital discharge. Length of stay in the study varied from 3 to 35 weeks. Wound areas were calculated digitally using ImageJ software from digital images of tracings and from wound photographs. Observers were blinded to each other's measurements. **Analysis:** Paired t-tests were used to examine differences between absolute measures; linear regression was applied to investigate consistency of difference over time. A mixed model design was used to examine differences between methods in wound size reduction with initial wound size included as a factor. **Results:** Absolute wound size was marginally different between the two methods (*p *= 0.05); the differences were consistent over time (*p *= 0.09). Reduction of wound size was similar using the two methods (*p*-value: 0.58). **Conclusions:** Although absolute wound size is not identical, reduction in wound size is similar whether wounds are traced or photographed. Therefore, clinical decision-making should not depend on the method used. Tracing is a simple and inexpensive outcome measure for monitoring wound healing.

# Abstract: A060 – Abdominal muscle activity in adults with and without Cystic Fibrosis

**Published:** 2010-07

**Authors:** A. Taillon-Hobson, L. McLean, S. Aaron, M. Bilodeau, Anne Taillon-Hobson, Linda McLean, Shawn Aaron, Martin Bilodeau

**Affiliations:** University of Ottawa, Faculty of Human Kinetics, Ottawa, Ontario, Canada; University of Ottawa, School of Human Kinetics, Faculty of Health Sciences, Ottawa, Ontario, Canada; Queen's University, School of Rehabilitation Therapy, Faculty of Health Sciences, Kingston, Ontario, Canada; Respiratory Medecine, Ottawa Hospital, Ottawa, Ontario, Canada; University of Ottawa, School of Rehabilitation Sciences, Faculty of Health Sciences, Ottawa, Ontario, Canada

**Purpose/Objectives and Rationale:** The repetitive use of the superficial abdominal muscles required for coughing may predispose individuals with cystic fibrosis (CF) to alterations in abdominal muscle recruitment relative to individuals without CF. Our purpose was to compare abdominal muscle recruitment patterns during abdominal hollowing exercises between adults with stable CF and age and gender matched control subjects. **Relevance to the Physiotherapy Profession:** Altered abdominal muscle recruitment patterns have been associated with musculo-skeletal problems, and may be of clinical importance as the literature suggests that individuals with CF may have a higher incidence of low back pain than the general population. **Materials and Methods:** Eighteen subjects (nine CF and nine controls) performed three different abdominal hollowing exercises using a pressure biofeedback unit (low, moderate and high pressure). Using surface electromyography, normalized EMG RMS amplitudes of the rectus abdominus, external obliquus, internal obliquus/transversus abdominis (IO/TrA), and multifidus were recorded during the exercises. **Analysis:** Data analysis was performed using Spike 2(5.06) software. Statistical analysis between groups, muscles and exercises were calculated using a mixed model analysis of variance (ANOVA). **Results:** EMG amplitudes increased from the low to high pressure tasks for all muscles (*p *< 0.001). EMG amplitude was higher for the IO/TrA (*p *< 0.001) than the other muscles, mostly during the low pressure task (muscle by exercise interaction, *p *= 0.02). However, our current data does not show any differences in muscle activation amplitudes between individuals with CF and controls (*p *= 0.95). **Conclusions:** Our initial analysis suggests that individuals with stable CF may not present with an altered ability to independently recruit superficial and deep abdominal muscles during abdominal hollowing exercises.

# Abstract: A065 – Community Balance & Mobility Scale (CB&M): Age-related reference values

**Published:** 2010-07

**Authors:** Kathryn Zbarsky, Dana Parsley, Heather Clegg, Tyler Welch, Catherine Fernandes, Susan Jaglal, Liz Inness, Josh Williams, William McIlroy, Jo-Anne Howe

**Affiliations:** 1 Department of Physical Therapy, University of Toronto, Toronto, Ontario, Canada, Department of Physical Therapy, University of Toronto, Toronto, Ontario M5G 1V7; 2 Toronto Rehabilitation Institute, Toronto, Ontario, M5G 2A2, Canada

**Purpose/Objectives and Rationale:** Purpose: To establish age-related reference values for the Community Balance and Mobility Scale (CB&M) and explore the relationship between CB&M Scores and Body Mass Index (BMI), strength, gait velocity and physical activity. Objectives: (1) Establish age-specific CB&M reference values among healthy adults aged 20–79 and examine the age-related variability. (2) Explore the relationship between CB&M scores with BMI, strength, gait velocity and physical activity measures. **Relevance to the Physiotherapy Profession:** Findings will aid clinicians in interpreting CB&M scores through comparison with healthy age-matched individuals. This may assist in early identification and treatment of balance deficits. **Materials and Methods:** 54 healthy participants aged 20–79 completed the CB&M, BMI, 10m Walk Test, grip strength, 30-sec chair stand and International Physical Activity Questionnaire(IPAQ). **Analysis:** Means, standard deviations, 95% confidence intervals were calculated for all variables. ANOVA and subsequent t-tests were then performed on CB&M data. Correlation coefficients were calculated between CB&M and each variable. A threshold where CB&M scores began to decline was identified and used to dichotomize data into two age categories. T-tests were used to determine between group differences in the variables. **Results:** CB&M scores decline after age 50 and a moderate correlation was identified between CB&M and BMI, 30-second chair stand and gait speed. Additionally, significant differences exist between dichotomized groups in strength, 30-second chair stand and gait speed. **Conclusions:** This study contributes to establishing age-related reference values for the CB&M and identified a decline in balance after age 50. BMI, strength and gait velocity were shown to effect balance abilities.

# Abstract: A079 – The clinically important improvement for the New Upper Extremity Functional Index

**Published:** 2010-07

**Authors:** D Yardley, M Benoit, T Blake, G Gillies, R Ho, M MacKinnon, PW Stratford, BM Chesworth

**Affiliations:** School of Rehabilitation Science and Department of Clinical Epidemiology and Biostatistics, McMaster University, Hamilton, ON L8S 1C7, Canada; (1) School of Physical Therapy, University of Western Ontario, London, ON N6G 1H1, Canada; (2) School of Physical Therapy and Department of Epidemiology and Biostatistics, University of Western Ontario, London, ON N6G 1H1, Canada

**Purpose/Objectives and Rationale:** The purpose was to determine the clinically important improvement for the revised version of the Upper Extremity Functional Index (UEFIv2). **Relevance to the Physiotherapy Profession:** Knowing outcome-measure change-score values that define clinically important improvement assists clinical decision-making. The clinically important difference for improvement in the UEFIv2 has not been established. **Materials and Methods:** We prospectively collected demographic and outcomes data for clients (*n *= 167) treated for musculoskeletal upper extremity (UE) dysfunction. At the initial visit and then three weeks later or discharge, whichever came first, participants completed the UEFIv2. At the second time-point, we also asked participants to rate their change in UE function compared to the first visit. Response options could be as small as ‘a tiny bit better/worse’ and as large as ‘a very great deal better/worse’. ‘No change’ was the midpoint. **Analysis:** We defined clinically important improvement three ways: ‘somewhat’, ‘moderately’ or ‘quite a bit’ better, and then used receiver-operator characteristic (ROC) curves to identify the UEFIv2 change-score that best defined those who achieved the threshold rating of improvement. **Results:** Clinically important improvement was 7/80 UEFIv2 scale points regardless of the definition of important change. The area under the ROC curve (95% confidence interval) varied from 0.82 (0.75, 0.87) for ‘somewhat better’ to 0.85 (0.79, 0.90) for ‘moderately better’ (*p *< 0.0001). For clients with musculoskeletal UE dysfunction, a change of at least 7 points in the UEFIv2 questionnaire represents clinically important improvement whether this is defined as “somewhat,” “moderately” or “quite a bit” better.

# Abstract: A080 – Physiotherapy Intervention in Occipital Neuralgia—A case-study

**Published:** 2010-07

**Authors:** SS Rege

**Affiliations:** Moncton Sports and Orthopaedic Physiotherapy, Moncton, NB, E1C9Y9, Canada

**Purpose/Objectives and Rationale:** Occipital neuralgia is a distinct type of headache caused by the irritation of the occipital nerve. Since occipital neuralgia is a rare occurrence, the primary treating physiotherapist can easily overlook the diagnosis. **Relevance to the Physiotherapy Profession:** Individuals with cervical-origin headaches are often referred to a physiotherapist for treatment. Inability to recognize the signs and symptoms of occipital neuralgia can adversely affect the prognosis of the individual. **Materials and Methods:** This paper highlights the case-study of a 48-year old female who presented to physiotherapy with complaints of sharp, shooting pain and extreme sensitivity to touch in the right occipital region, dizziness and blurry vision following a traumatic work injury. Initial investigations at the emergency department were negative for fractures of the skull. **Analysis:** Pain ratings via the Visual Analogue Scale, subjective reports of sensitivity in the occipital region and active range of motion measurements of the cervical spine were obtained regularly. **Results:** Physiotherapy intervention immediately post-injury was limited to pain control, postural education and restoring active range of motion of the cervical spine. A desensitization and cervical stabilization program was undertaken after achieving pain control. 3-months post-injury, the client had regained full active range of motion of the cervical spine and was able to tolerate light touch to the right occipital region. The client underwent an occipital nerve block 5-months post injury subsequent to which she was able to resume her pre-injury work. **Conclusions:** Although occipital neuralgia is rare, knowledge of the pathology, etiology, signs and symptoms will ensure appropriate referral and treatment interventions.

# Abstract: A082 – The role of PT and OT in the Post-Biologic Era—A survey of patients

**Published:** 2010-07

**Authors:** AJ Busch, A Dooley, S Coons, K Crockett, J Mucha

**Affiliations:** School of Physical Therapy, University of Saskatchewan, Saskatoon, SK S7N 0W3

**Purpose/Objectives and Rationale:** To describe opinions regarding access to, effects, and relevance of physical therapy (PT) and occupational therapy (OT) for individuals with arthritis in the post-biologic era. **Relevance to the Physiotherapy Profession:** ‘Biologic response modifiers’ have revolutionized the medical management of rheumatoid arthritis (RA). Biologics are used for patients whose arthritis does not respond to disease-modifying anti-rheumatic drugs (DMARDs). Influences of biologics on pathological processes associated with rheumatic arthritis (RA) are greatly diminished for many individuals. Increasing prevalence of biologics demands re-evaluation of the role of OT and PT in the management of RA. **Materials and Methods:** A cross-section survey design was used. Survey questions were developed and emailed to members of Canadian Arthritis Patient Alliance. **Analysis:** Thematic analysis of responses to six open-ended questions was conducted. **Results:** There was an enthusiastic response to the survey; within 3 weeks, 26 responses were received (58% response rate). Diseases represented: OA, RA, AS, Lupus, Polymyalgia rheumatica, PsA and Spondylitis. Provinces represented: BC, AB, SK, MB, ON, PQ, NB, and NS. Common themes such as the need for an interdisciplinary team, PT as a vital role, and accessibility problems, presented throughout the surveys, demonstrating PT/OT as key components in the management of rheumatic diseases, with limitations in access and delivery of these services.**Conclusions:** Responses indicate PT and OT as integral parts of successful management and treatment regimen. Clients who receive PT/OT seem to have better control of their arthritis, ultimately leading to improved quality of life.

# Abstract: A087 – Effect of preoperative resistance training for patients undergoing high tibial osteotomy: A prospective cohort study with historical controls

**Published:** 2010-07

**Authors:** CO Kean, TB Birmingham, LK King, JR Giffin

**Affiliations:** * Wolf Orthopaedic Biomechanics Laboratory, Fowler Kennedy Sport Medicine Clinic, University of Western Ontario, London, ON N6A 3K7; ^ School of Physical Therapy, University of Western Ontario, London, ON N6A 3K7

**Purpose/Objectives and Rationale:** An important goal of high tibial osteotomy (HTO) is to increase physical activity and function in relatively young, active patients with knee osteoarthritis. However, post-operative recovery requires prolonged protected-weight-bearing and activity limitations that result in substantial deficits in muscular strength. The purpose of this study was to examine the effects of pre-operative high intensity resistance training on post-operative outcomes after HTO. **Relevance to the Physiotherapy Profession:** Prehabilitation that focuses on strength training may help achieve greater improvements in physical activity and function after HTO. **Materials and Methods:** Fourteen patients (48.1±7.2 yrs; 13M, 1F) scheduled for medial opening wedge HTO completed a 12-week pre-operative high intensity resistance training program focusing on isokinetic quadriceps and hamstrings strength. These patients were matched to baseline clinical and demographic characteristics of 14 patients who previously received an HTO without pre-operative training and were participants in a larger prospective cohort study. All outcomes were measured before and 6 months after surgery. The Sport and Recreation subscale of the Knee injury and Osteoarthritis Outcome Score (KOOS) was the primary outcome. Secondary outcomes included other KOOS subscales and the Lower Extremity Functional Scale. **Analysis:** We compared 6-month outcomes using independent *t*-tests. **Results:** The pre-operative training group achieved greater scores in Sport and Recreation [mean difference = 16.29 (95% CI: 1.97, 30.89)] and Activities of Daily Living [mean difference = 8.40 (95% CI: 0.53, 16.75)]. There were no significant differences between groups on other outcomes. **Conclusions:** Pre-operative resistance training before medial opening wedge HTO improves post-operative physical activity and function.

# Abstract: A089 – Motivation and practice environments for upper limb motor recovery in stroke

**Published:** 2010-07

**Authors:** Christiane B. Lourenço, Sandeep Subramanian, Heidi Sveistrup, Mindy F. Levin

**Affiliations:** (1) School of Physical and Occupational Therapy, McGill University & CRIR; (2) Faculty of Health Sciences, University of Ottawa

**Purpose/Objectives and Rationale:** Better rehabilitation outcomes are linked to higher motivation in animal models of stroke recovery. Virtual reality environments (VE) have been proposed to increase participant's level of engagement and motivation in performing motor tasks. Our aim was to estimate the effect of different training environments and motivation on motor recovery in the upper limb in stroke patients. **Relevance to the Physiotherapy Profession:** This study will inform neurological physiotherapy practice on the effectiveness of different training environments. **Materials and Methods:** Participants with stroke were randomly allocated to practice reaching tasks in two training environments (G1: physical, G2: virtual) over 4wks (12 sessions). Kinematic and clinical evaluations were done before, after and 3mos after training. Level of motivation and self-efficacy were also assessed. **Analysis:** Kinematic and clinical outcomes were compared using non-parametric statistics. Spearmen correlations determined relationships between variables. **Results:** There was significant improvement in elbow extension for G1 (*p *< 0.05) on the reaching performance scale. Both groups improved but greater improvements in shoulder flexion, shoulder horizontal adduction, endpoint speed and precision occurred in G2. Improvements in shoulder flexion and speed were retained at follow-up. Subscales of Intrinsic Motivation Inventory for both groups occurred between Interest/Enjoyment and Value/Usefulness (G1 r = 0.98, *p *< 0.001; G2 r = 0.98, *p *< 0.001) and between Interest/Enjoyment and Perceived Competence (G1 r = 0.83, *p *< 0.05, G2 r = 0.87, *p *< 0.05). There was only correlation between Interest/Enjoyment and Effort/Importance for G1 (r = 0.91, *p *< 0.005). **Conclusions:** Results indicate that patients could use feedback when delivered in a virtual environment and that this type of feedback delivery may lead to better motor outcomes.

# Abstract: A095 – Deleterious effects of pre-surgery wait on health-related quality of life and contralateral knee pain six months after primary total knee replacement

**Published:** 2010-07

**Authors:** FRANÇOIS DESMEULES, CLERMONT E. DIONNE, ÉTIENNE L. BELZILE, RENÉE BOURBONNAIS, PIERRE FRÉMONT

**Affiliations:** 1 Population Health research unit (URESP), FRSQ Research Centre of Laval University Affiliated Hospital (CHA), Quebec, QC, Canada; 2 Department of Social and Preventive Medicine, Faculty of Medicine, Laval University, Quebec, QC, Canada; 3 Department of Rehabilitation, Faculty of Medicine, Laval University, Quebec, QC, Canada; 4 Laval University Hospital (CHUQ), Quebec, QC, Canada; 5 Community Health Care Centre (CSSS) de la Vieille-Capitale, Quebec, QC, Canada

**Purpose/Objectives and Rationale:** To assess, in patients scheduled for total knee replacement (TKR), the effects of pre-surgery wait on pain and functional limitations related to the knee joint undergoing surgery, on overall health-related quality of life (HRQoL) and on contralateral knee pain six months after surgery. **Relevance to the Physiotherapy Profession:** Prehabilitation programs for TKR have positive effects on the patients' health status. A better understanding of the effects of pre-surgery wait on TKR patients would help optimize these programs. **Materials and Methods:** 143 patients scheduled for TKR were recruited and followed up for 6 months after surgery. Pre-surgery wait was defined as the time between enrolment on the waiting list and surgery. Pain and functional limitations were measured with the Western Ontario and McMaster Arthritis Index (WOMAC). HRQoL was measured with the SF-36. **Analysis:** An analysis of covariance was conducted to compare outcomes 6 months after surgery between categories of pre-surgery wait (≤3, > 3–6, > 6–9, > 9–12, > 12 months), while adjusting for confounders.

**Results:** Six months after TKR, there was a significant difference in HRQoL SF-36 role physical (*p *= 0.012) and contralateral knee WOMAC pain (*p *= 0.002) scores, participants having waited > 9–12 and > 12 months showing the worst condition. **Conclusions:** Longer pre-surgery wait has a negative impact on HRQoL and contralateral knee pain six months after TKR. This study emphasizes the importance of wait time reduction and suggests that further research should evaluate the effect of prehabilitation programs that include treatment of the contralateral knee.

# Abstract: P004 – Canadian best practice recommendations for the rehabilitation of persons after stroke

**Published:** 2010-07

**Authors:** CL Richards, M MacKay-Lyons

**Affiliations:** Department of Rehabilitation, Laval University and CIRRIS Research Center, Quebec, Qc, G1K7P4 and School of Physiotherapy, Dalhousie University, Halifax, NS, B3H4R2, Canada; SCORE Research Team

**Learning Objectives and Session Content:** The objectives of this proposal are:

1. to describe the methodology and the process that led to the development of the Canadian recommendations for the rehabilitation of persons after stroke,
2. to present the recommendations for the rehabilitation of the arm, the leg and walking,
3. to suggest treatment protocols to accompany these recommendations, and
4. to place these recommendations within the context of the Canadian Stroke Strategy that covers the continuum of care from stroke onset to community integration.

The session will include Powerpoint presentations by the two presenters to deliver the salient information. The delivery of these presentations will be interspersed with periods of discussion with the participants at the end of each section. When presenting the recommendations for rehabilitation of the arm, leg and walking, certain aspects will be emphasized and lead to theoretical and practical discussions.

**Relevance to the Physiotherapy Profession:** This is a knowledge translation proposal that will present and explain how a team of researchers, clinicians, funding agencies and patients worked together over a seven year period to contribute to the Canadian Stroke Strategy and, more specifically, to develop recommendations and protocols for the rehabilitation of persons after stroke. Those attending this session will learn how such recommendations and protocols were derived (i.e., key published articles, literature searches, methods used to analyse and score the literature, group discussions, analysis and scoring of existing treatment guidelines, choice of protocols, etc.). Because the recommendations will be presented along with the strength of supporting evidence from basic and clinical studies, participants will have the opportunity to better understand the state of current evidence and the need for further studies in certain areas. Participants will also be provided with examples of treatment protocols to meet the recommendations. Finally, it is imperative that physiotherapists be informed of the Canadian Stroke Strategy, the availability of key articles and booklets describing different aspects of this strategy and the important role of rehabilitation within this strategy.

**Target Population:** The target population can be seen in two ways: the recommendation and protocols to be presented relate to the rehabilitation of persons after stroke, but the target population can be interpreted as physiotherapists who are motivated to uptake these best practice recommendations and modify their treatment approaches for the rehabilitation of persons after stroke.

**Description of Supporting Evidence:** Participants will be provided with:

1. The Canadian Stroke Network's Recommendations for Rehabilitation of the Arm, the Leg and Walking in both English and French.
2. Selected protocols from the literature.
3. Reference lists to delve further into the source of the recommendations and the protocols.
4. The Canadian Stroke Strategy Canadian Best Practice Recommendations for Stroke Care (updated 2008) [Patrice Lindsay, BScN PhD, Mark Bayley, MD, Chelsea Hellings, BScH, Michael Hill, MSc MD, Elizabeth Woodbury, BCom MHA, Stephen Phillips, MBBS. Canadian Stroke Strategy Best Practices and Standards Writing Group on behalf of the Canadian Stroke Strategy, a joint initiative of the Canadian Stroke Network and the Heart and Stroke Foundation of Canada*CMAJ 2008 179: S1-S25].
5. Several booklets published by the CSN, including “A Patient's Guide to Canadian Best Practice Recommendations for Stroke Care and Getting on with the Rest of your Life.”
6. List of websites such as the Evidence-Based Review of Stroke Rehabilitation, StrokEngine and StrokeAssess to help clinicians keep up to date with the evolving evidence in the literature.

**Description of Session Format:** The session will include Powerpoint presentations interspersed with discussions at the end of each section. In addition, some aspects of the recommended treatment approach such as task oriented training and cardiovascular fitness training will be emphasized with the use of case histories and videos. The two speakers will develop the presentation material according to their individual expertise. Participants will be encouraged to ask questions and enter into discussions with the presenters.

**Conclusions and Implications:** Seven years have gone into the creation of the Canadian Best Practice Recommendations for Stroke Care. It is important that Canadian physiotherapists be introduced to these Best Practice Recommendations and in particular those that pertain to rehabilitation. The co-presenters were members of the team that created the recommendations for rehabilitation and they will be able to provide first-hand knowledge of the process and encourage clinicians to incorporate them in their daily practice.

# Abstract: P006 – Causation, “the chicken or the egg”: Epidemiological aspects of whiplash associated disorders for physiotherapists in the medical legal setting?

**Published:** 2010-07

**Authors:** GM Schneider, AD Smith

**Affiliations:** University of Calgary, Faculty of Medicine, Calgary. Alberta, Canada; Advanced Spinal Care Centre (EFW Radiology), Calgary, Alberta, Canada; LifeMark Health, Calgary, Alberta, Canada; University of Queensland, Brisbane, Australia; Advanced Spinal Care Centre (EFW Radiology), Calgary, Alberta, Canada

**Learning Objectives and Session Content:** Our presentation will include an overview of epidemiology as it pertains to causation (determination of cause and effect). Concepts related to causation derived from the well established Bradford-Hill criteria as well as those from a leading modern epidemiologist, Dr. Kenneth Rothman, will be presented. The education session will discuss and apply principles of causation as they relate to patients with whiplash injury. Common clinical examples will be discussed to aid the physiotherapist in a primary care setting in determining causation in a medical legal context. The objectives of our education session are as follows:

1. To aide the participant in understanding the elements of causation as related to the determination of injury following a motor vehicle collision.
2. To provide the participant with necessary information to be able to apply the elements of causation in an epidemiological framework to determine cause and effect between their patients' whiplash injury and motor vehicle collision.
3. To allow the participants to work through specific case examples so that they can apply epidemiological methods in disputed matters.

**Relevance to the Physiotherapy Profession:** As primary care providers in an evolving health care system, Physiotherapist's are relied upon as experts in musculoskeletal health. Patient's suffering from whiplash injury commonly present to physiotherapy clinics across Canada. As insurance company directives are evolving, Physiotherapists are now performing independent examinations in order to provide expert opinions as to the optimal management of patients with Whiplash Associated Disorders (WAD). As a result, Physiotherapist's are being called into the medical legal environment in the domain of an expert witness. Up to 60% of individuals following whiplash injury will suffer persistent pain and disability at six months post collision. Although many factors contribute to persistent pain and disability, cause and effect is less clear to many practitioners. Therefore, it is valuable for Physiotherapist's to understand the epidemiological framework surrounding causation so that they can effectively represent themselves as an expert witness in the medical legal setting.

**Target Population:** This presentation will be ideally suited for Physiotherapists, Physicians, and other health care professionals working in a primary care setting.

**Description of Supporting Evidence:** Modern epidemiological constructs as described above will be utilized in conjunction with validated outcome measures performed in chronic WAD patients. The reliability, sensitivity and specificity of these measures will be discussed to allow attendees to make informed decisions regarding their practice requirements. These measures have been validated and utilized by various international authors in both randomized controlled trials and observational research in chronic WAD patients.

**Description of Session Format:** This presentation will accompany a lecture format. Group discussion will occur at the end of the presentation to facilitate transfer of knowledge and clarity of the concepts. Practical examples will be incorporated throughout the presentation to facilitate transfer of knowledge into clinical practice.

**Conclusions and Implications:** Appropriate use of epidemiological methods by Physiotherapist's and other primary care providers can facilitate suitable opinions surrounding causation in cases of whiplash injury in a medical legal context. As primary health care providers involved in the day-to-day management of patients with WAD, Physiotherapist's are in an ideal position to provide expert opinion in cases involving causation. This is not typical practice in our current medical legal system, but there exists an emerging role for Physiotherapists' in this regard secondary to their expertise in musculoskeletal medicine.

# Abstract: P013 – Exploring the development, impact and relevance of a Spinal Triage Assessment Program provided by physiotherapists in collaboration with orthopaedic surgeons

**Published:** 2010-07

**Authors:** B Bath, R Bourassa, S Lovo Grona

**Affiliations:** Bourassa and Associates Rehabilitation Centre; University of Saskatchewan, School of Physical Therapy, Saskatoon, SK Canada

**Learning Objectives and Session Content:** The learning objectives of this session are:

1. to describe the factors which lead to the development of a successful spinal triage program provided by physiotherapists in collaboration with orthopaedic surgeons,
2. to identify and discuss the essential components of a clinical examination, including screening for serious pathology, for patients presenting with lumbar spine-related problems and
3. to apply a clinical reasoning approach to the diagnosis and management of spinal problems using three case study examples.

*Section 1:* Description of the Service. The Wall Street Spinal Assessment Service (WSSAS) is a collaborative effort between a group of three orthopaedic surgeons and a private rehabilitation clinic that has been operating since 2003. This section will review the context of how and why this service was initiated, the referral and clinical care pathways, the assessment process, initial program evaluation results, further ongoing research initiatives, and how the service is evolving to meet the needs of its users and the growing complexity of clinical cases.

*Section 2:* Spinal Clinical Examination.This section will review the essential components of a spinal examination focusing on the lower quadrant. An electronic database assessment system will be introduced and explored and those sections pertaining to screening for serious pathology will be highlighted.

*Section 3:* Case Studies.In this section three case studies will be presented to reflect the diversity of the clinical problems that can be encountered in a spinal triage program. The cases will range from mechanical back pain requiring mechanically-based treatment to serious spinal/medical pathologies requiring medical and/or surgical interventions. The history and physical exam findings,the recommended treatment pathways, diagnostic tests and/ or follow-up as well as any relevant outcomes will be presented and discussed for each case.

**Relevance to the Physiotherapy Profession:** Low back pain and low back-related disorders are significant population health problems which consume a large amount of health care resources. Primary care reform entails improving efficiency, coordination, and continuity to ensure health needs are met in the right place at the right time by the appropriate health care provider(s). Physiotherapists with advanced orthopaedic training have the necessary skills and knowledge to effectively and efficiently assess and help to manage people with spinal problems. The use of physiotherapists in a spinal triage setting, such as the WSSAS, has the potential to streamline primary care management of low back-related disorders and reduce wait times for secondary and tertiary care. Additionally, the model of health care delivery presented in this session reflects a growing trend in the Physiotherapy profession of expanding roles and scope that is applicable in a number of different practice settings.

**Target Population:** This session is relevant to a wide ranging audience including: experienced and novice physiotherapy clinicians,students, clinical managers and other health care providers. Participants with an orthopaedic background and interest would likely be most interested in the clinical content delivered in this session; however, the collaborative model of health care presented would be of interest to physiotherapists working in other practice areas.

**Description of Supporting Evidence:** People with a variety of low back-related complaints comprise a large proportion of referrals made to orthopaedic surgeons. Many of these patients are not considered to be surgical candidates and may simply require reassurance that they do not have serious spine pathology. However, those people that do have serious pathology need to be identified and appropriately managed. The use of other primary health care providers that have expertise in assessment and evaluation of musculoskeletal disorders is an alternative approach to this problem. Physiotherapists with advanced orthopaedic training, often practicing with an extended scope, have been shown to be equally as effective as orthopaedic surgeons for the diagnosis and non-surgical management of many musculoskeletal conditions. Physiotherapists performing this role have also demonstrated significant positive impacts on wait times and referral practices.

**Description of Session Format:** The format of this presentation will be a combination of lecture, small and large group discussion. Section 1 & 2 will primarily be lecture (facilitated by power point slides) and large group discussion. A summary of the clinical findings of each of the case studies (Section 3) will be presented as handouts to the participants augmented by any relevant de-identified video and/or pictures. Small group discussion of the clinical presentation regarding diagnosis and treatment recommendations will ensue followed by a large group discussion. Finally, the clinical outcomes of each case will be presented with opportunities for large group discussion.

**Conclusions and Implications:** Primary health care providers assessing people with spinal problems need to differentiate between the small number of serious pathologies and the vast majority of benign conditions. Therefore, the confidence and ability to apply appropriate clinical reasoning when assessing people with back pain and related symptoms is an essential skill for primary care providers, including physiotherapists, to have. Increasing the efficiency of an orthopedic surgeon's caseload by reducing the number of non-surgical consultations is one potential means to decrease wait times. Since its inception, the WSSAS has dramatically reduced the participating surgeons' case loads for low back-related conditions. If a similar model of triage assessment were adopted by other orthopaedic surgeons with high case-load proportions of non-surgical low back related conditions, then reduction in wait times would inevitably occur. Finally, further system-wide improvements would be possible if physiotherapists were afforded opportunities of triage assessment and primary care management that expanded to other types of health conditions.

# Abstract: P018 – Is there a role for physiotherapists in the management of chronic wounds?

**Published:** 2010-07

**Authors:** EL Nussbaum, S Gabison

**Affiliations:** Department of Physical Therapy, University of Toronto and Department Rehabilitation, Mount Sinai Hospital, Toronto, Canada; Department of Physical Therapy, University of Toronto

**Learning Objectives and Session Content:** Participants will be able to:

1. assess and stage wounds,
2. understand risk factors for wound development and delayed wound healing,
3. explain the role and limitations of physical agents in management of wounds,
4. describe ultraviolet-C radiation (UV-C), ultrasound, and electrical stimulation procedures for wounds, and
5. appreciate the importance of a multidisciplinary approach to the whole patient with a wound.

**Content includes:**

1. wound pathophysiology,
2. risk factors (intrinsic and environmental),
3. wound assessment tools,
4. cleansing and selecting the appropriate product to dress wounds,
5. introduction to compression bandaging,
6. discussion of ultrasound, UV-C and electrical stimulation (ES) effects on wound healing,
7. indications and contraindications, and
8. demonstration of UV-C and ES treatment techniques using wound models.

**Relevance to the Physiotherapy Profession:** Chronic wounds are a significant cause of morbidity and mortality and for many people pressure ulcers are a recurring problem. In certain populations, e.g., persons with spinal cord injury, the incidence of pressure ulcers may be up to 95% over the course of a lifetime. Chronic wounds impact on the individual's finances, time, and overall well-being as they may interfere with ability to sustain high levels of independence and/or participate in the community. Wounds increase hospital length of stay and prescribed bed rest may prevent participation in rehabilitation. Activity restriction can give rise to respiratory complications, increase the risk of thrombotic events and lead to declining strength. In 2006, a nursing practice audit in the Niagara Region of Ontario showed that care for patients with wounds cost on average $2326 per patient; ulcer healing averaged 21 weeks. In the Ottawa Region a nursing practice survey covering a 1-month period found 531 clients with active below-knee ulcers, of which about 33% had persisted for longer than 12 months and about 60% for longer than 6 months. Osteoarthritis (51%), cardiac disease (47%), and diabetes (46%) were the most common co-morbid conditions. Nurses had made 2270 visits to 192 of the clients with wounds that month. This data suggests that nursing intervention alone is not enough. A treatment that is non-invasive, painless and promotes tissue healing without risk to the patient and that reduces the burden of wounds on individuals and healthcare systems would be a huge advantage. Physical agents such as ultrasound, ultraviolet-C irradiation (UV-C), and electrical stimulation (ES) might provide such an opportunity.

**Target Population:** Physiotherapists and physiotherapy assistants who treat adult clients with wounds, whether hospitalized or living in long term care institutions or independently in the community with conditions affecting the cardio-respiratory, neurological and musculoskeletal systems.

**Description of Supporting Evidence:** There is extensive literature covering normal tissue repair and delayed wound healing. Management of wounds from nursing (dressings primarily) and pharmacological/medical (topical preparations, growth factors, human skin equivalents, etc,) perspectives have been extensively studied. In spite of some advanced therapies meta-analysis has failed to demonstrate superiority of any one treatment to accelerate healing. Best-practice in wound assessment has been established. Photodocumentation and digital measurement of wound area are commonly used. The reliability and accuracy of different measures are reported in the literature. Reduction of wound area is commonly used as a basis for treatment decisions. Systematic reviews on effects of physical agents on chronic wounds are mostly inconclusive because many of the individual studies suffer from methodological limitations, small number of participants in individual trials and heterogeneity among trials in methods of treatment and measurement. For example, a 2006 Cochrane review of ultrasound effects on pressure ulcers included three randomized controlled trials (RCTs). The conclusion was that there was no evidence of benefit; however, ‘the possibility of benefit or harmful effect could not be ruled out due to the small numbers of trials, some with methodological limitations, and small numbers of participants.’ Similarly, a 2008 Cochrane review of ultrasound on venous ulcers, comprising eight RCTs, concluded that ultrasound treatment healed more ulcers and reduced the percentage ulcer area remaining; however, the conclusions were compromised due to methodological limitations in the individual RCTs. There are no large RCTs of UV-C effects on wounds. Small controlled trials, numerous case studies, animal and cellular studies suggest that UV-C has potential to accelerate healing and to clear wounds of bacteria. Kill rates have been documented up to 99.9% in vitro for vancomycin-resistant Enterococcus (VRE), Group A ß St.

**Description of Session Format:** The session includes:

1. a formal slide presentation with a paper copy of slides given to participants,
2. video-demonstration of application of ultrasound, UV-C and electrical stimulation to wounds,
3. a hand-out of color photographs of actual wounds for participants to engage in an exercise in staging wounds and prescribing dose of ultrasound, UV-C and ES treatment,
4. question and discussion opportunities, 5. wound models and equipment will be made available for participants to practice application of UV-C and/or ES with the presenter's feedback, but not necessarily within the time frame of the session.

**Conclusions and Implications:** Physiotherapists are becoming increasingly involved in wound care management. Ontario legislation will soon increase physiotherapists' scope of practice in care of wounds. Effective physiotherapy for wounds requires more than just the knowledge of how electrophysical agents interact with biological tissues. This session aims to give physiotherapists a broad understanding of factors that impact on wound healing. The use of electrophysical agents for wound healing is mostly dependent on Level II evidence because of a lack of large randomized placebo-controlled efficacy trials. This session will present a critique of systematic reviews and individual trials in an attempt to elucidate the most beneficial dosing parameters when using ultrasound, UV-C and ES. Following this session, and clinical skills practice for those who choose to avail themselves of the opportunity, participants will be able to apply the new knowledge immediately in clinical practice.

# Abstract: P033 – When the room is spinning what do you do? An overview of vestibular assessment for the physiotherapist

**Published:** 2010-07

**Authors:** KJ Schneider

**Affiliations:** University of Calgary, Faculty of Kinesiology, Calgary, AB, Canada

**Learning Objectives and Session Content:** This session will provide a general overview of vestibular dizziness and include a practical component to learn/perform specific tests to assess the peripheral vestibular system. Learning objectives:

1. Provide an overview of the anatomy and physiology of the vestibular system.
2. Outline the signs and symptoms of common vestibular problems, specifically Benign Paroxysmal Positional Vertigo and peripheral vestibular hypofunction.
3. Discuss and practice commonly used evidence-based clinical tests for vestibular involvement.

**Relevance to the Physiotherapy Profession:** Dizziness is a commonly encountered problem in the physiotherapy clinic that may lead to decreased self-health ratings, decreased mobility, decreased social activity and secondary health problems. Vestibular dysfunction may be reported or present with many orthopaedic and neurological conditions. Identification of vestibular involvement will allow for timely treatment and/or further referral for such problems and facilitate treatment and return to health.

**Target Population:** This session is relevant to a wide range of physiotherapists.

**Description of Supporting Evidence:** Vestibular related dizziness is a commonly occurring problem in the general population. Studies have shown that individuals with peripheral vestibular dysfunction benefit from vestibular rehabilitation. Early identification of individuals with vestibular involvement using currently available, validated clinical tests will facilitate treatment of the affected area and maximize recovery.

**Description of Session Format:** This session will be an interactive lecture that includes description and practice of clinical tests for vestibular involvement and case studies to demonstrate a typical patient with peripheral vestibular dysfunction.

**Conclusions and Implications:** Dizziness is commonly reported in the physiotherapy clinic but may be difficult to differentially diagnose. An understanding of the history and physical exam findings that suggest vestibular involvement will facilitate timely identification and treatment of this commonly occuring problem.

# Abstract: S004 – Patient safety—Where do we draw the lines in clinical practice? (An evidence-based inter-divisional knowledge translation symposium)

**Published:** 2010-07

**Authors:** Carolyn Emery, Kathleen Norman

**Background:** The most current interdivisional journal of the CPA was titled “Safety in Practice”. Safety is clearly a focus in many areas of practice as we challenge our patient populations to maximize function and independence. In many areas of practice, physiotherapists use techniques and modalities in practice wherein there is a tension between safety and therapeutic benefit. This symposium will highlight some examples of areas of practice in different patient populations to which therapeutic techniques pose known risks and those risks must be evaluated against the need for safety in order to maximize patient outcomes. Our presenters include leading clinicians and researchers who work across a diversity of populations that will share their expertise in the field and provide an evidence-based approach to safety aspects of practice. This workshop is intended to cross the interests of multiple CPA Divisions of practice.

**Overall Objective:** 1. To be exposed to examples of safety balanced against risk in a variety of patient populations from an evidence-based physiotherapy perspective.

**Target Population:** This workshop will be of interest to a broad range of physiotherapists including clinicians, managers, professional leaders, educators, and researchers interested in maximizing patient outcomes in rehabilitation settings where safety is clearly a priority.

**Speakers and Topics:**

Description of Symposium—Kathleen Norman
Topic A. Safety of Manual Therapy in Osteoporotic Spine—Meena Sran
Topic B. Return to play guidelines following a concussion: The ‘developmental’ dilemma in paediatric sport—Kathryn Schneider
Topic C. Balancing patient risk-taking and safety among patients, families, and clinicians during transitions in care from brain injury rehabilitation—Angie Andreoli
Topic D. Metamorphosis from fearful to adventurous after you have been told your heart is at risk—Diana Hopkins-Rosseel
Summary—Dr. Kathleen Norman

# Abstract: S011 – Increasing physical activity and exercise tolerance in people with compromised health—An Evidence-based Physiotherapy Roundtable

**Published:** 2010-07

**Authors:** Linda McLean, Neil Pearson, Linda Woodhouse, Diana Hopkins-Rosseel

**Background:** Primary Health Care initiatives are often structured within a chronic disease management model where health care providers act as facilitators. Individuals are educated and encouraged to self manage a variety of conditions, and actively participate in a variety of initiatives (including increasing their physical activity levels) to help them feel better and minimize any complications arising from their problem. Physiotherapists are key players within this model, as their practice focuses on improving function and preventing negative consequences of a variety of chronic diseases and conditions. We can apply our expertise in exercise prescription.

This roundtable will explore the limitations of various health problems and their impact on individuals' abilities to participate in exercies and activity, consider the role of the physiotherapist in the management of these clients, and consider the evidence regarding the efficacy of various approaches.

Roundtable panelists include both clinicians and researchers, and discussion will reflect the role of physiotherapists working in a variety of clinical settings, practice areas and patient populations.

**Overall Objectives:**

1. To explore the limitations of different health problems, and their impact on the individuals' abilities to participate in exercise and activity,
2. To consider to the role of the physiotherapist in the management of these clients from an evidence-based physiotherapy perspective,
3. To consider the evidence regarding the effectiveness of various approaches.

**Target Population:** This workshop will be of interest to a broad range of physiotherapists including clinicians, managers, professional leaders, educators, and researchers interested in the implementation of best practices in programs to increase the activity levels and exercise tolerance of individuals in these groups.

**Speakers and Topics:**

Introduction—Carolyn Emery
Topic A. Activity and Exercise Tolerance and Chronic Neuromuscular Pain—Neil Pearson
Topic B. Activity and Exercise Tolerance and Cardiovascular Disease—Diana Hopkins-Rosseel
Topic C. Activity and Exercise Tolerance and Incontinence—Linda McLean
Topic D. Activity and Exercise Tolerance in individuals who are Arthritic or Obese—Linda Woodhouse
Moderator Summery—Carolyn Emery

# Abstract: S015 – Paediatric Concussion—A continuum of evidence

**Published:** 2010-07

**Authors:** Carolyn Emery, Lucie Pelland, Rob Werstine, Kathryn Schneider

**Background:** Participation of youth in collision and high impact sports is significant in Canada. The incidence of concussion in pediatric sport is high and the public health impact is significant. This symposium will provide participants with evidence related to pediatric concussion across a continuum of research from basic science, through clinical and population health. The symposium is intended to inform physiotherapy practice related the prevention, recognition and treatment of concussion in child and adolescent sport.

**Overall Objectives:**

1. To be exposed to a continuum of evidence regarding concussion in pediatric sport from basic science, through clinical and population health.
2. To examine evidenced-based perspectives regarding the prevention, recognition and treatment of pediatric concussion

**Target Population:** This workshop will be of interest to a broad range of physiotherapists who work in public or private practice settings and in pediatrics, neurology, orthopaedics and/or sport.

**Speakers and Topics:**

Topic A. Epidemiology of concussion in youth sport—Impact, risk and prevention—Carolyn Emery
Topic B. Recognition and neurodevelopmental implications of concussion in pediatric sport—Lucie Pelland
Topic C. What is the role of neurocognitive testing in pediatric concussion in sport?—Rob Werstine
Topic D. Is there a role for imaging in pediatric concussion?—Lara Boyd
Topic E. The role of vestibular rehabilitation in the treatment of concussion in youth—Kathryn Schneider
